# Ivermectin inhibits ER, HER2, and TGF-β pathways in ER-positive and endocrine-resistant breast cancer cells

**DOI:** 10.1371/journal.pone.0348260

**Published:** 2026-04-30

**Authors:** Kitiya Rujimongkon, Patthamapon Adchariyasakulchai, Chanoknun Boonyaratsewee, Kantasorn Horpratraporn, Wannarasmi Ketchart

**Affiliations:** 1 Department of Pharmacology, Faculty of Medicine, Chulalongkorn University, Bangkok, Thailand; 2 Department of Chemistry, Faculty of Science, Chulalongkorn University, Bangkok, Thailand; University of Wisconsin-Madison, UNITED STATES OF AMERICA

## Abstract

Ivermectin (IVM), initially developed as an antiparasitic drug, has recently demonstrated notable anticancer activity in several malignancies, including breast cancer. Our previous work showed that IVM inhibited cell proliferation and invasion in breast cancer cells, primarily through modulation of the Wnt signaling pathway. Preliminary results further indicated that combining IVM with tamoxifen enhanced its antiproliferative effects in endocrine-resistant breast cancer cells, suggesting a potential role in overcoming drug resistance. To further clarify IVM’s mechanism in the context of endocrine resistance, this study investigated its effects on estrogen receptor-positive (ER⁺) and endocrine-resistant breast cancer cells. IVM significantly suppressed estrogen-induced proliferation and downregulated key resistance-associated markers, including ER and HER2. Additionally, IVM treatment markedly reduced ERK, a downstream effector linked to HER2 and TGF-β signaling. This inhibition was accompanied by a decrease in phosphorylated SMAD2 (pSMAD2) within the TGF-β pathway, while levels of SMAD4—a factor associated with favorable prognosis in endocrine resistance—were maintained. Collectively, these findings highlight IVM’s potential as a repurposed therapeutic agent, with the dual capacity to prevent endocrine resistance in ER⁺ breast cancer and to enhance anti-hormonal therapies in resistant cases.

## Introduction

Ivermectin (IVM), a derivative of avermectin produced by *Streptomyces avermitilis*, has been clinically used as an antiparasitic agent for over four decades, particularly in the treatment of onchocerciasis and intestinal strongyloidiasis in humans for over four decades. [1]. Beyond its antiparasitic role, IVM has demonstrated anticancer activity in multiple malignancies, including breast cancer [2]. Previous studies have shown that IVM inhibits several key signaling pathways, such as AKT/mTOR, Wnt/β-catenin, Heat Shock Protein 27 (HSP27), and P21-activated kinase 1 (PAK-1) in breast cancer [3–6], as well as and mitogen-activated protein kinase (MAPK) signaling in melanoma and nasopharyngeal carcinoma [7,8]. Our earlier work also reported that IVM suppressed the growth of ER-positive and endocrine-resistant breast cancer cells through modulation of Wnt pathway, with potential to reverse endocrine resistance [9]. Endocrine-resistant cells are known to exhibit upregulated human epidermal growth factor receptor 2 (HER2) signaling, which in turn activates downstream pathways including AKT, Extracellular Signal-Regulated Kinase (ERK), and MAPK [10–13].

Studies investigating the role of IVM in reversing tumor multidrug resistance (MDR) have shown that it suppresses MDR-associated proteins and P-glycoprotein (P-gp), both of which are key contributors to chemotherapy resistance. IVM was found to overcome MDR in vincristine-resistant colon cancer cells, breast cancer (MCF-7), and leukemia (K562) cells by inhibiting Epidermal Growth Factor Receptor (EGFR) signaling and its downstream effectors ERK, Akt, and Nuclear Factor kappa-light-chain-enhancer of activated B cells (NF-κB), ultimately leading to reduced P-gp expression [14]. In addition, IVM inhibited phosphorylation of HSP27, a chaperone protein commonly overexpressed in drug-resistant cancers and associated with poor prognosis. By blocking HSP27 phosphorylation, IVM enhanced the efficacy of anti-EGFR therapies in cancers expressing EGFR/HER2 [6].

Estrogen plays a central role in driving the growth and progression of estrogen receptor (ER)-positive (ER⁺) breast cancer cells. Selective estrogen receptor modulators (SERMs), such as tamoxifen, are widely used as systemic adjuvant therapy following surgery [15]. However, resistance to tamoxifen can arise during treatment or after the standard five-year course [16]. In such cases, selective estrogen receptor degraders (SERDs), like fulvestrant, are employed to target and degrade ER. Recent evidence indicates that endocrine therapies—including SERMs and SERDs—may downregulate SMAD4, a key mediator of the TGF-β pathway, thereby contributing to endocrine resistance and poorer prognosis [17]. SMAD proteins are critical mediators of TGF-β signaling, and prior studies have shown cross-talk between the Wnt and TGF-β pathways in breast cancer [18]. Our previous work demonstrated that IVM inhibits Wnt pathway mediators [9], prompting further investigation into its potential role in modulating TGF-β signaling. Mechanisms of endocrine resistance include loss of ER expression, observed in approximately 20% of cases where initially ER-positive tumors become ER-negative, rendering them unresponsive to ER-targeted therapies [19]. Another key mechanism, seen in approximately 50% of ER-positive tumors, is overexpression of HER2 [20]. In SMAD4-deficient breast cancer cells, combined inhibition of ER and HER2 has been shown to significantly enhance autophagy, potentially improving endocrine resistance [21]. Although IVM has been reported to influence PAK-1, a factor implicated in tamoxifen resistance [4], its effects on other mechanisms of endocrine resistance remain unclear. The present study, therefore aims to further elucidate the role of IVM in overcoming endocrine resistance.

## Materials and methods

### Cell lines and cultures

The ER-positive breast cancer cell lines: MCF-7 and T-47D, along with the tamoxifen-resistant T-47D Tam1, were obtained from the American Type Culture Collection: ATCC (Virginia, USA). The tamoxifen-resistant MCF-7/LCC2 and the tamoxifen and fulvestrant-resistant MCF-7/LCC9 cell lines were generously provided by Dr. Robert Clarke (Lombardi Cancer Center, Georgetown University, Washington, DC, USA). The fulvestrant-resistant T47D-182R1 cells were obtained from the ECACC General Cell Collection (Salisbury, UK; ECACC 16022507). The normal skin fibroblast CRL-1474 cell line was provided by Department of Research and Development, Queen Saovabha Memorial Institute. MCF-7/LCC2 and MCF-7/LCC9 cells were maintained in MEM supplemented with 5% FBS. T-47D cells were cultured in RPMI 1640 supplemented with 10% FBS and 10 μg/mL human insulin. T-47D Tam1 cells were maintained in RPMI 1640 containing 10% FBS, 10 μg/mL human insulin, and 1 μM 4-hydroxytamoxifen (4-OHT). T47D-182R1 cells were maintained in phenol red-free RPMI 1640 supplemented with 5% FBS, GlutaMAX, 8 μg/mL insulin, and 100 nM fulvestrant. CRL-1474 cells were maintained in DMEM supplemented with 10% FBS. All cell lines were cultured at 37°C in a humidified incubator with 5% CO₂. To ensure phenotype stability, MCF-7/LCC2, MCF-7/LCC9, T-47D Tam1, and T47D-182R1 cells were routinely tested for resistance to tamoxifen or fulvestrant using 3-(4,5-dimethyl-2-thiazolyl)-2,5-diphenyl-2H-tetrazolium bromide (MTT) assays (S1 Table).

### Reagents

IVM, MTT, 4-hydroxytamoxifen (4-OHT), and 17β-estradiol (E_2_) were obtained from Sigma-Aldrich (Missouri, USA). Primer pairs for GAPDH and ESR1 were synthesized by Integrated DNA Technologies (IDT, USA). Antibodies against AKT, Cyclin D1, ERK, ESR1, HER2, pHER2, mTOR, PAK-1, PI3K, SMAD2, SMAD3, SMAD4, their respective phosphorylated forms, and GAPDH were from Cell Signaling Technology (Massachusetts, USA).

### MTT assays

MCF-7, MCF-7/LCC2, MCF-7/LCC9, T-47D, T-47D Tam1, and T47D-182R1 cells were seeded in 96-well plates at a density of 5 × 10^3^ cells per well and cultured overnight. To determine IC₅₀ values, cells were treated with IVM at concentrations ranging from 0.15 to 50 µM (twofold serial dilutions) for 24 h. For time-dependent assay, the cells were treated with IVM for 24, 48 and 72 h. A 0.01% dimethyl sulfoxide (DMSO) solution served as the vehicle control. Following treatment, an MTT assay was performed by adding 0.5 mg/mL MTT solution in phosphate-buffered saline to each well and incubating for 4 h. Formazan crystals were solubilized in DMSO, and absorbance was measured at 570 nm using a microplate reader. Cell viability was expressed as a percentage relative to the control, calculated using the formula: (OD sample/OD control) × 100.

### Selectivity index

CRL-1474 cells were placed in 96-well plates and exposed to varying concentrations of IVM alone or in combination with 4-OHT. After 24 hours of treatment, MTT assays were performed as previously described. The IC_50_ values of IVM in CRL-1474 cells were determined and compared to the IC_50_ values in MCF-7 cells to assess the selectivity index.

### Estrogen-induced cell proliferation

ER-positive (MCF-7 and T-47D) cells were cultured in phenol red-free media before experiments. MCF-7 cells were cultured in DMEM and phenol red-free IMEM supplemented with 5% charcoal dextran-treated FBS for at least 4 days, whereas T-47D cells were maintained in phenol red-free RPMI 1640 with 5% charcoal dextran-treated FBS overnight before experiments. For MTT assays, cells were seeded in 96-well plates at a density of 5 × 10³ cells/well in phenol red–free medium overnight. Cells were then treated with IVM (1, 3, or 5 µM) in the presence or absence of 10-nM estradiol (E_2_) for 5 days. 4-OHT (5 or 10 µM) served as a positive control. Media containing the respective drug concentrations were refreshed 3 days after treatment initiation. Following incubation, MTT assays were performed, and cell viability was calculated as a percentage relative to the control.

### Combined treatment of IVM and 4-OHT

MCF-7, MCF-7/LCC2, and MCF-7/LCC9 cells were cultured in phenol red-free media supplemented with 5% charcoal dextran-treated FBS prior to experimentation. CRL-1474 and MCF-7 cells were used to assess the safety of combined treatment. For MTT assays, cells were seeded into 96-well plates and incubated overnight, while for protein analysis, they were plated into 24-well plates. Cells were then treated with 4-OHT and/or IVM at the IC₂₅ concentration of each drug for 24 h before performing MTT assays or Western blot analysis.

### Drug combination analysis

Raw data from Rujimongkon et al. (2025) [9] were analyzed under the Creative Commons Attribution 4.0 International (CC BY) license. In the study, 4-OHT was used at concentrations of 0, 2.5, 5, 7.5, and 10 µM. MCF-7 cells were treated with IVM at 0.5, 2, and 8 µM, MCF-7/LCC2 cells at 4, 7, and 9 µM, and MCF-7/LCC9 cells at 3, 5, and 7 µM for 72 h. Following treatment, MTT assays were conducted to assess % cell viability under single or combined drug conditions. The interaction between IVM and 4-OHT was evaluated using the Chou–Talalay method, with isobologram and combination index (CI) analyses performed via CompuSyn software (CompuSyn, Inc., Paramus, NJ). CI values were interpreted as follows: CI < 1, synergistic effect; CI = 1, additive effect; CI > 1, antagonistic effect. The CI was calculated using the formula: CI = [(D)_1_/(IC_50_)_1_] + [(D)_2_/(IC_50_)_2_], where (IC50)_1_ and (IC50)_2_ represent the concentrations of IVM and 4-OHT that inhibit 50% of cell survival (Fa = 0.5), and (D)_1_ and (D)_2_ are the concentrations of IVM and 4-OHT in combination that achieve the same effect. The dose-reduction index (DRI) was also calculated to compare the inhibitory effects of IVM and 4-OHT individually versus in combination, using: (DRI)_1_ = (IC_50_)_1_/(D)_1_ and (DRI)_2_ = (IC_50_)_2_/(D)_2_.

### Reverse transcription polymerase chain reaction

MCF-7 cells were treated with IVM at concentrations of 0, 8, 10, and 13 µM, with or without 10 nM E_2_, for 24 h before collection for RNA isolation. Total RNA was extracted using GENEzol™ Reagent (Geneaid Biotech, Taiwan), and RNA quantity and purity were assessed with a NanoDrop-One spectrophotometer (Thermo Scientific, US). Approximately 0.5 µg of total RNA was reverse-transcribed into cDNA using the ImProm-II™ Reverse Transcription System (Promega, U.S.) and subsequently amplified with primers specific for *estrogen receptor 1* (*ESR1*), *pS2* and *GAPDH*. Real-time PCR was performed on the Azure Cielo Real-time PCR System (Azure Biosystems, US), and relative gene expression was analyzed by the ΔΔCt method, with GAPDH as the internal control.

### Western blot analysis

The treatments of IVM at 3, 6, and 9 µM were added to MCF-7, MCF-7/LCC2, and MCF-7/LCC9 cells for 24 h, with 0.01% DMSO serving as the non-treatment control. To evaluate the impact of IVM on estrogen-stimulated cells, MCF-7 cells were exposed to IVM at 0, 8, 10, and 13 µM with or without 10 nM E_2_ for 24 h. For combination studies, cells were treated with IC25 concentrations of IVM and/or 4-OHT for 24 h. After treatment, cells were lysed as previously described [22]. Equal amounts of protein were separated on 8% SDS-PAGE and transferred onto nitrocellulose membranes. 5% nonfat milk was added to membranes for 1 h at ambient temperature, followed by overnight incubation at 4°C with primary antibodies diluted in 5% bovine serum albumin in TBS-T (0.1% Tween 20). After three washes with TBS-T, membranes were incubated with HRP-conjugated anti-rabbit secondary antibody in blocking solution. Protein bands were visualized, and relative intensities were quantified against GAPDH using Image Studio 5.2 software (LICOR, Lincoln, USA).

### Ethics approval

This study was exempted by the Institutional Review Board of the Faculty of Medicine, Chulalongkorn University (IRB Number: 0530/65, COE No.039/2022).

### Statistical analysis

Data are presented as mean ± SEM from at least three independent experiments. Statistical analysis between the nontreatment control and experimental groups were calculated using one-way ANOVA followed by Dunnett’s post hoc test. For E_2_-induced cell experiments, two-way ANOVA was applied to assess the effects of IVM at various concentrations in the presence or absence of E_2_. Comparisons between two groups were performed using Student’s t-test. A *p-*value <0.05 was considered statistically significant. All analyses were conducted by Prism 10 software (Chicago, IL, USA).

## Results

### Ivermectin exhibited anti-cancer effects in ER⁺ and endocrine-resistant breast cancer cells, showing selective anti-cancer activity over normal cells

Our previous study demonstrated the anticancer activity of IVM in wild-type MCF-7 cells and their resistant counterparts, including tamoxifen-resistant MCF-7/LCC2 and fulvestrant-resistant MCF-7/LCC9 cells [9]. In the present study, we extended this investigation to T-47D ER-positive cells and their resistant derivatives, tamoxifen-resistant T-47D Tam1 and fulvestrant-resistant T47D-182R1 cells. The findings revealed that the IC_50_ values of MCF-7 and T-47D cells were within a comparable range (Table 1). Moreover, no significant differences in IC_50_ values were observed between the parental cell lines and their resistant variants. These results indicate that IVM exerts potent inhibitory effects in ER-positive and endocrine-resistant breast cancer cells. The inhibitory effect of IVM was investigated across different treatment durations. The result indicated that IVM has time-dependent effects in all MCF-7 cell lines (S1 Fig).

**Table 1:** Half inhibitory concentration (IC_50_) values of IVM in breast cancer cell lines at 24-h treatment.

Cell line	IVM IC_50_ (µM)	Cell line	IVM IC_50_ (µM)
	24 h		24 h
MCF-7	11.44 ± 0.25	T-47D	10.29 ± 0.20
MCF-7/LCC2	9.62 ± 0.42	T-47D Tam1	10.27 ± 0.44
MCF-7/LCC9	9.28 ± 0.18	T47D-182R1	10.32 ± 0.14
Mean (µM) ± SEM			

The safety of IVM was first assessed using normal fibroblast CRL-1474 cells. IVM had an IC_50_ value of 41.67 µM after 24 hours of treatment. The selectivity indexes for IVM, compared with MCF-7, MCF-7/LCC2, and MCF-7/LCC9 cells, were greater than 3 (S2 Table). Additionally, combining IVM with 4-OHT, the standard treatment for ER^+^ breast cancer cells, reduced cell viability in MCF-7 cells compared with CRL-1474 cells (S2 Fig). These results indicate that IVM could have an acceptable safety profile *in vitro*.

### IVM lowered ERα and HER2 without affecting SMAD4 in ER⁺ and endocrine-resistant breast cancer cells, and reduced ER-target genes only in ER⁺ cell lines

**Fig 1:**
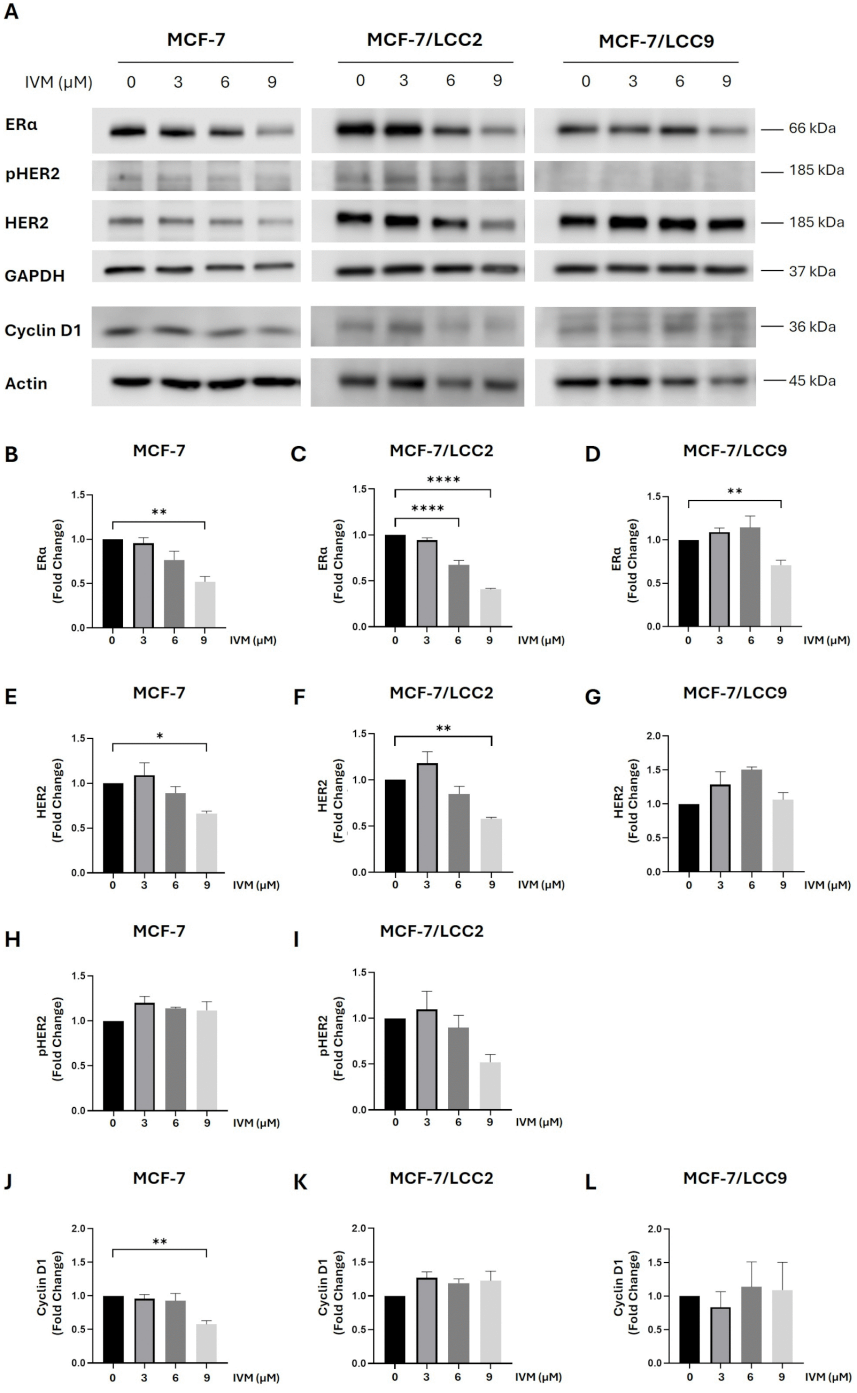
IVM reduced ERα and HER2 in ER⁺ and endocrine-resistant cell lines. **Additionally, IVM decreased cyclin D1 levels in ER**^**+**^ **cells: MCF-7, MCF-7/LCC2, MCF-7/LCC9. (A)** Protein levels were evaluated by Western blotting after treating cells with various concentrations of IVM for 24 **h.** The graphs demonstrated the inhibitory effect of IVM on the protein levels of **(B–D)** ERα, **(E–G)** HER2, **(H-I)** pHER2, and **(K-L)** Cyclin D1. The data were shown as mean ± SEM. *p < 0.05, **p < 0.01, ***p < 0.001, ***p < 0.0001 compared with the nontreatment control (n = 3)..

Because IVM has demonstrated strong activity in cells resistant to endocrine therapy, its impact on ERα, HER2, and SMAD4, which are crucial mediators linked to endocrine resistance [21], and Cyclin D1, which is an ER-target gene, were examined. IVM significantly suppressed ERα and HER2 protein levels at 9 µM in ER-positive MCF-7 cells (Figs 1A-1B, and 1E). In tamoxifen-resistant MCF-7/LCC2 cells, ERα was markedly reduced at 6 and 9 µM, while HER2 was significantly decreased at 9 µM (Figs 1A, 1C, and 1F). In fulvestrant-resistant MCF-7/LCC9 cells, IVM significantly lowered ERα at 9 µM and did not change HER2 level (Figs 1A, 1D, and 1G). IVM did not change pHER2 levels in MCF-7 and MCF-7/LCC2 (Figs 1A, 1H, and 1I), and the pHER2 bands in MCF-7/LCC9 could not be detected (Figs 1A). In addition, IVM significantly decreased Cyclin D1 at 9 µM only in MCF-7 cells. (Figs 1A, and 1J) and did not change Cyclin D1 in MCF-7/LCC2 and MCF-7/LCC9 (Figs 1A, 1K and 1L). IVM also significantly decreased *pS2* mRNA, another ER-target gene in MCF-7 cells (S3 Fig). Notably, IVM did not alter SMAD4 expression across MCF-7 cell lines (S4A–4D Figs). Furthermore, IVM significantly inhibited pPAK-1 and PAK1—previously implicated in tamoxifen resistance [23]—but only in tamoxifen-resistant MCF-7/LCC2 cells at 9 µM (S4H–4J Figs). Collectively, these findings suggest that IVM reduces ERα and HER2 expression, supports the maintenance of SMAD4 in both wild-type and resistant cells, and specifically targets pPAK-1/PAK1 signaling in tamoxifen-resistant cells. This indicates that IVM modulates key mediators involved in tamoxifen resistance. Moreover, IVM inhibited ER and ER-target genes only in ER-positive breast cancer cells, suggesting the inhibitory mechanism of IVM in ER signaling.

### IVM inhibited estrogen-induced cell proliferation and ERα and HER2 levels in ER-positive breast cancer cell lines

**Fig 2:**
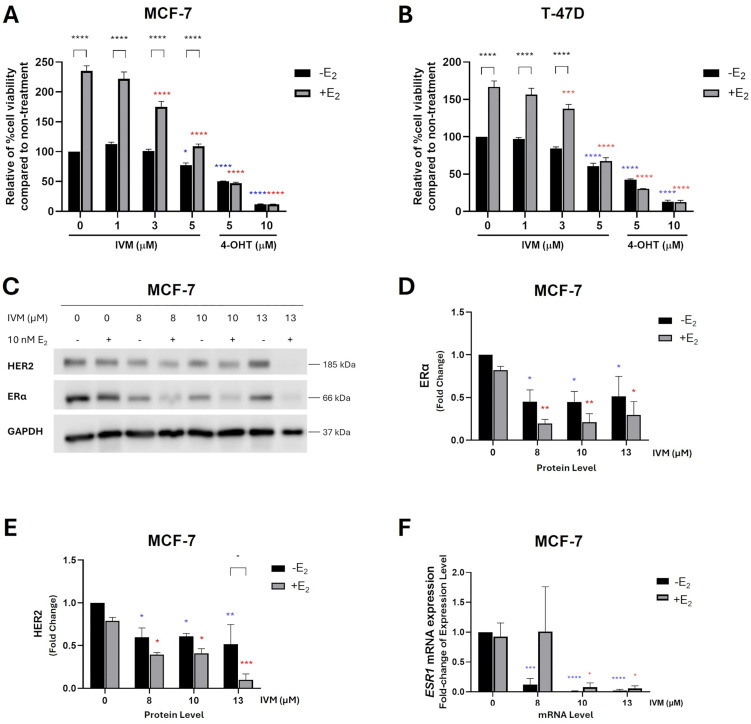
IVM inhibited estrogen-induced cell proliferation in ER⁺ breast cancer cell lines: MCF-7 and T-47D. MTT assay measured cell viability of ER-positive breast cancer cells: **(A)** MCF-7 and **(B)** T-47D, were treated with (+E_2_) or without (−E_2_) 17β-estradiol at physiologic level (10 nM E_2_) in the presence of nontoxic concentrations of IVM (1, 3, 5 μM) or 4-OHT (5, 10 μM) as a positive control for 5 days. **(C–E)** The inhibitory effect of IVM on ERα and HER2 protein levels of ER-positive breast cancer was performed after the treatments with various concentrations of IVM with (+E_2_) or without (−E_2_) 17β-estradiol (10 nM E_2_) for 24 h in MCF-7. **(F)** mRNA expression of the *ESR1* gene (ERα) was determined by qPCR analysis in MCF-7 cells. The graphs showed the mean ± SEM. The blue and red asterisks highlight the significant differences in comparison to non-treatment without (−E_2_) and with (+E_2_), respectively. *p < 0.05, **p < 0.01, ***p < 0.001, ****p < 0.0001 *versus* the non-treatment control (n = 3).

Considering that our study demonstrated IVM’s inhibitory effects on ERα and ER-targeted genes in ER-positive breast cancer cells, we next examined its impact in the presence of the potent endogenous estrogen, 17β-estradiol (E_2_), to better mimic physiological conditions. E_2_ significantly promoted cell proliferation in ER-positive breast cancer cells, including MCF-7 and T-47D (Figs 2A and 2B). Treatment with IVM at 3 and 5 µM markedly suppressed E_2_-induced proliferation in both cell lines (Figs 2A and 2B). The inhibitory effect of IVM on E_2_-induced cell proliferation was in a concentration-dependent manner, as shown in S5A-5B Figs. Moreover, IVM in combination with E_2_ further reduced ERα and HER2 protein levels (Figs 2C–2E). To clarify whether this inhibitory effect was linked to ERα regulation, we assessed *ESR1* mRNA expression, which encodes ERα, and found it was also reduced by IVM in MCF-7 cells (Fig 2F). Collectively, these findings indicate that IVM strongly suppresses E_2_-induced cell proliferation while downregulating ERα in ER-positive breast cancer cells.

However, E_2_ failed to induce cell proliferation in MCF-7/LCC2 and MCF-7/LCC9 cells. IVM inhibited cell growth equally with or without E_2_ in these resistant lines (S5C-5D Figs). This aligns with prior findings that IVM does not affect ER-target genes in endocrine-resistant cells (Figs 1A, 1K and 1L), indicating that basal ER levels mediate IVM’s effects on estradiol–ER signaling.

### Synergistic effect of IVM and 4-OHT on restoring tamoxifen sensitivity in ER⁺ and endocrine-resistant cell lines

**Fig 3:**
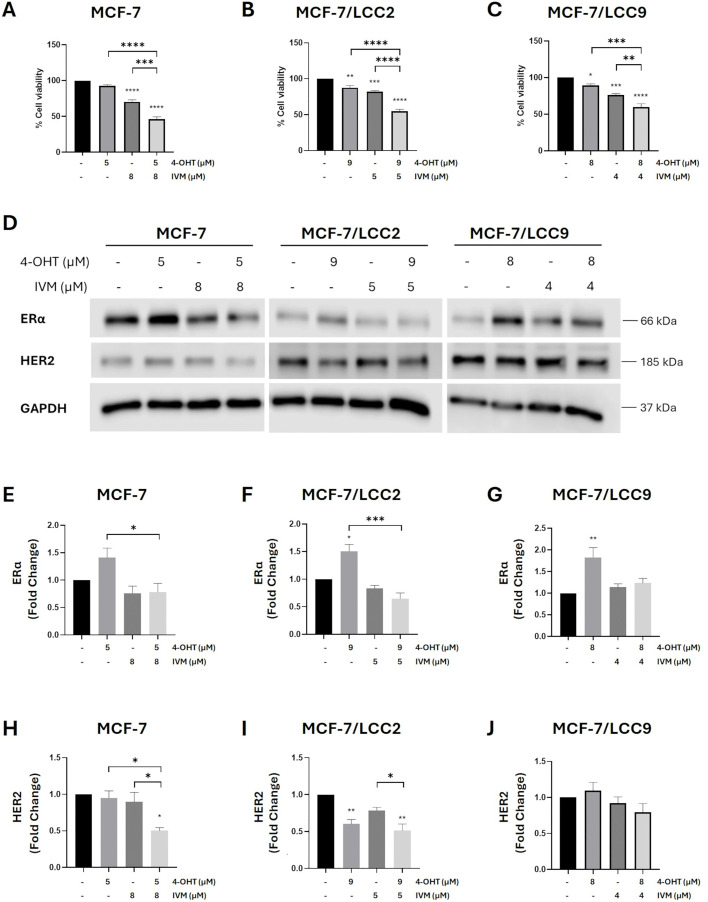
Combined treatment of IVM and 4-OHT further reduced cell viability and ERα and HER2 levels in ER⁺ and endocrine-resistant breast cancer cell lines. IC_25_ values of IVM and/or 4-OHT-treated concentrations were obtained from MTT assays. Cell viability was evaluated in three MCF-7 breast cancer cell lines: **(A)** MCF-7, **(B)** MCF-7/LCC2, and **(C)** MCF-7/LCC9 at 24 **h.** The graphs showed the mean ± SEM at each treated concentration. **p < 0.01, ***p < 0.001, ****p < 0.0001 compared with nontreatment control by one-way ANOVA (n = 3). **(D)** Western blots were performed in three MCF-7 breast cancer cell lines after the treatments with concentrations at IC_25_ values of IVM and/or 4-OHT for 24 **h.** The fold change on the protein level of ERα was assessed in **(E)** MCF-7, **(F)** MCF-7/LCC2, and **(G)** MCF-7/LCC9 cells and HER2 in **(H)** MCF-7, **(I)** MCF-7/LCC2, and **(J)** MCF-7/LCC9 cells. The graphs displayed the mean ± SEM. *p < 0.05, **p < 0.01, and ***p < 0.001, comparing between two groups (n = 3).

Our previous study reported that combined IVM with 4-OHT significantly decreased ce

ll viability in endocrine-resistant cells [9]. In this study, we further evaluated the combination index of the combined treatment to determine whether it provides a synergistic or additive effect. The approximate IC_25_ concentrations of IVM (S3 Table) were selected for combination with 4-OHT for 24 hours. The combined treatment significantly suppressed cell proliferation compared to monotherapy across all cell lines (Figs 3A–3C). In MCF-7 and MCF-7/LCC2 cells, the combination also produced a greater reduction in ERα levels than 4-OHT alone (Figs 3D–3F). Similarly, HER2 expression was markedly decreased with the combined treatment compared to both the non-treatment and monotherapy groups (Figs 3H and 3I). In MCF-7/LCC9 cells, however, the combination showed only a trend toward reducing ERα and HER2, likely due to ERα upregulation induced by 4-OHT (Figs 3G and 3J). Consistent findings were observed in T-47D cell lines, where IVM and 4-OHT together further decreased ERα in T-47D and T-47D Tam1 cells (S6A–S6C Figs). ERα could not be detected in T47D-182R1 cells due to the presence of fulvestrant in the culture medium. Nevertheless, the combination treatment further reduced HER2 levels in T-47D, T-47D Tam1, and T47D-182R1 cells (S6A, S6D–F Figs).

Furthermore, reanalysis of supplementary data from our previous study [9], conducted under the PLOS One Creative Commons Attribution 4.0 International (CC BY) license, confirmed that IVM acts synergistically with 4-OHT at various concentrations (CI < 1) (S7 Fig). These results suggest that IVM enhances the efficacy of 4-OHT and may allow for dose reduction in ER-positive and tamoxifen-resistant breast cancer cells.

### Inhibitory effects of IVM on the TGF-β signaling pathway in ER⁺ and endocrine-resistant breast cancer cell lines

**Fig 4:**
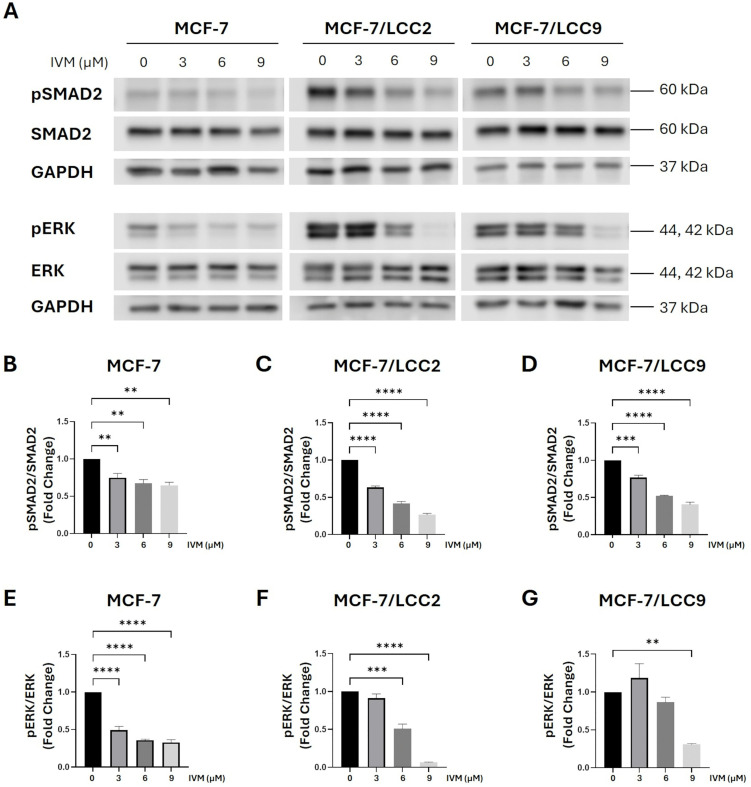
IVM inhibited pSMAD2 and pERK involved in the TGF-β signaling pathway in ER-positive and endocrine-resistant breast cancer cell lines: MCF-7, MCF-7/LCC2, MCF-7/LCC9. **(A)** The protein levels were determined by Western blot after the treatment with various concentrations of IVM for 24 **h.** The graphs demonstrated the inhibitory effect of IVM as the fold change in protein levels of **(B–D)** pSMAD2/SMAD2 and **(E–G)** pERK/ERK in MCF-7, MCF-7/LCC2, and MCF-7/LCC9 cells, respectively. The data were shown as mean ± SEM. *p < 0.05, **p < 0.01, ***p < 0.001, ***p < 0.0001 compared with the nontreatment control (n = 3).

Our previous experiment indicated that IVM inhibits HER2. Consequently, the downstream ERK signaling pathway, which regulates TGF-β by phosphorylating SMAD2 and SMAD3 [24] was examined. Additionally, our earlier study revealed that IVM suppressed Wnt signaling and reduced epithelial–mesenchymal transition (EMT)-associated markers [9]. Since Wnt and TGF-β signaling synergistically regulate EMT [25], we therefore examined the impact of IVM on the TGF-β pathway. IVM significantly inhibited pSMAD2 across all tested concentrations and in all cell lines (Figs 4A–4D). In MCF-7 cells, pERK was markedly reduced at all concentrations of IVM (Figs 4A and 4E), while in MCF-7/LCC2 cells, significant inhibition was observed at 6 and 9 µM (Figs 4A and 4F), and in MCF-7/LCC9 cells at 9 µM (Figs 4A and 4G). IVM also significantly suppressed pSMAD3 levels in MCF-7 and MCF-7/LCC2 cells at 6 and 9 µM. Conversely, in MCF-7/LCC9 cells, pSMAD3 levels increased following 3 µM treatment but showed a downward trend at 6 and 9 µM (S4A and S4E-S4G Figs). By contrast, IVM did not significantly alter other key mediators associated with endocrine resistance pathways, including PI3K, AKT, and mTOR (S8A–S8J Figs).

## Discussion

Estrogen, particularly 17β-estradiol (E_2_), the most potent endogenous form, plays a critical role in breast cancer tumorigenesis by binding to the ER and forming an estrogen–ER complex. This complex binds to promoter regions of ER-responsive genes, driving their transcription. The resulting gene activation promotes cell proliferation and progression in ER-positive breast cancer, the most common subtype [15]. However, approximately half of advanced-stage ER-positive patients fail to respond to tamoxifen and eventually develop recurrence or metastasis [26–28]. Our study observed the increased ER level after 4-OHT treatment in MCF-7 cells. This finding is consistent with Fan et al. and is described as a biological response [29]. The increase of ER reflects how cells adapt to selective estrogen receptor modulators. This finding is explained by compensatory upregulation that occurs when ER signaling is inhibited. 4‑OHT stabilizes ERα, reduces its degradation, and relieves estrogen‑mediated negative feedback, leading to higher ER expression despite reduced transcriptional activity [29]. Fulvestrant, another treatment option, acts as a pure ER antagonist across hormone-sensitive tissues and is often used for tamoxifen-resistant or advanced metastatic breast cancer [30]. Nonetheless, prolonged fulvestrant therapy also leads to acquired resistance in most patients [31]. Collectively, patients resistant to these therapies are categorized as endocrine-resistant. Considering the limited treatment options for ER-positive breast cancer due to drug resistance and adverse effects, novel therapeutic targets are urgently needed to overcome endocrine resistance and reduce recurrence and metastasis.

IVM, an antiparasitic agent derived from *Streptomyces avermitilis*, has gained attention for its potential anticancer effects across multiple cancer types, including breast cancer [2]. Our previous study demonstrated that IVM suppresses cell proliferation, migration, and invasion through inhibition of the Wnt signaling pathway. Furthermore, combining IVM with tamoxifen enhanced the antiproliferative effect in endocrine-resistant cells, indicating a possible reversal of tamoxifen resistance. In the present study, IVM was shown to downregulate *ESR1* mRNA expression, which encodes ER, and reduce ER protein levels. IVM also decreased the expression of ER-targeted genes, including mRNA of *pS2* and Cyclin D1 protein*.* Its inhibitory effects on both proliferation and gene expression were more pronounced in estrogen-stimulated cells compared with non-estrogen-treated cells. However, E_2_-induced cell proliferation and the inhibitory effect of IVM on the ER-target gene were absent in the resistant cell lines. Thus, IVM’s effect relies on the baseline ER level expressed in breast cancer cells, given that resistant cell lines show reduced ER expression. Consistent with these findings, IVM has been reported to disrupt reproductive functions in animals by lowering serum estradiol levels or preventing pregnancy [32–34], supporting its involvement in the estradiol–ER signaling pathway. Additionally, combination studies revealed a synergistic effect between IVM and tamoxifen, further supporting its potential to overcome endocrine resistance. Beyond breast cancer, IVM has been shown to suppress MDR in vincristine-resistant colon cancer cells, MCF-7 breast cancer cells, and K562 leukemic cells by targeting EGFR and inhibiting downstream pathways such as ERK, Akt, and NF-κB, ultimately reducing P-glycoprotein (P-gp) expression [14].

Alterations in growth factor signaling can drive cancer progression through both genomic and non-genomic mechanisms, ultimately contributing to tamoxifen resistance. This includes the activation and overexpression of tyrosine kinase receptors, such as HER2, as well as pathways like MAPK, PI3K/AKT, stress signaling, and insulin-like growth factor signaling [26]. Activation of these pathways enhances ER activity and elevates the levels of ER coactivators and downstream effectors—including cyclins, cyclin-dependent kinases, and their inhibitors—that regulate the cell cycle and promote breast cancer cell survival and proliferation. Clinical evidence further demonstrates associations between ER, HER2, p38, and ERK (extracellular signal-regulated kinases) in tamoxifen-resistant tumor samples, supporting the idea that crosstalk between ER and growth factor signaling serves as an adaptive mechanism to circumvent tamoxifen-mediated inhibition of ER activity [35]. In our study, IVM significantly inhibited HER2 in wild-type ER-positive and tamoxifen-resistant breast cancer cell lines. In these two cell lines with detectable pHER2 bands, IVM did not noticeably alter pHER levels. This outcome could be due to a decrease in total HER2 protein within these cells. IVM did not affect HER2 level in fulvestrant-resistant cells. Interestingly, the effects of IVM were more pronounced in tamoxifen-resistant cells compared with fulvestrant-resistant cells, despite similar IC_50_ values. This observation is consistent with our previous findings using zoledronic acid, where differential mechanisms appeared between the two resistant subtypes [13]. Ongoing studies will further explore these distinct mechanisms in tamoxifen- versus fulvestrant-resistant breast cancer cells. Collectively, these findings showed that IVM significantly reduces both ER and HER2, two key drivers of endocrine resistance that engage in reciprocal crosstalk to reinforce each other’s signaling [16]. However, IVM did not alter several key signaling molecules within the HER2 pathway, including PI3K, p‑AKT, and p‑mTOR, which play important roles in endocrine resistance. This suggests that the anti‑tumor effects of IVM may instead involve other HER2‑related or downstream mechanisms.

ERK, a downstream effector of HER2 signaling, regulates TGF-β signaling by phosphorylating the linker regions of SMAD2 and SMAD3 proteins [36]. This phosphorylation modulates their activity, subcellular localization, and transcriptional functions, thereby shaping the cellular outcomes of TGF-β signaling. ERK-mediated phosphorylation represents a key crosstalk mechanism between the MAPK and SMAD pathways, fine-tuning processes such as proliferation, differentiation, and apoptosis [24]. TGF‑β was shown to regulate ERK1/2 activation in ER-negative MDA‑MB‑231 breast cancer cells, as TGF‑β knockdown suppressed pERK1/2, while exogenous TGF‑β restored it [37]. Overall, the data support an interplay between TGF‑β and ERK signaling. Moreover, ERK was reported to phosphorylate ERα in breast cancer cells [38]. Therefore, ERK is a key crosstalk in the signaling pathways involved in endocrine resistance.

The SMAD family proteins are the canonical intracellular mediators of TGF‑β signaling, forming the core pathway through which TGF‑β exerts its transcriptional and phenotypic effects. Upon ligand binding, TGF‑β receptors phosphorylate the receptor‑regulated SMADs (SMAD2/3), which subsequently form heteromeric complexes with SMAD4 and translocate into the nucleus to regulate target gene expression [39]. SMAD2 and SMAD3, central mediators of TGF-β signaling, exert complex—and sometimes opposing—roles in breast cancer progression, including the suppression of metastasis [40]. Through this SMAD‑dependent axis, TGF‑β orchestrates key cellular processes such as proliferation, differentiation, apoptosis, and extracellular matrix remodeling. Dysregulation of TGF‑β/SMAD signaling is a well‑established driver of EMT, enhanced cell migration and invasion, and tissue fibrosis, largely through SMAD2/3‑dependent transcriptional programs that upregulate mesenchymal markers and promote cytoskeletal reorganization and motility [39]. Consistent with these functional roles, our previous work demonstrated that IVM suppressed EMT markers and reduced cell invasion in endocrine-resistant breast cancer cells [9]. In this study, IVM not only inhibited ERK but also significantly reduced SMAD2 phosphorylation. While inhibition of pSMAD2, which has been linked to tumorigenesis, may appear counterintuitive, IVM concurrently suppressed other critical growth signaling pathways, including ER and HER2. This broad inhibitory effect contributes to its potent anti-proliferative activity in both ER-positive and endocrine-resistant cells. Moreover, the reduction of pSMAD2 may provide additional benefits in limiting metastasis, consistent with our earlier findings that IVM significantly suppressed cell invasion [9].

The role of SMAD4 in endocrine resistance, particularly in ER⁺ breast cancer, has gained increasing attention. Both clinical and preclinical studies indicate that SMAD4 downregulation is frequently linked to tamoxifen resistance, suggesting its broader contribution to reduced treatment efficacy across ER⁺ subtypes [21]. Mechanistically, loss of SMAD4 impairs TGF-β–mediated growth suppression, enabling estrogen-independent proliferation, epithelial-to-mesenchymal transition, and the acquisition of stem-like phenotypes [21,41]. These alterations contribute to more aggressive and therapy-resistant tumor characteristics. Furthermore, SMAD4 modulates ERα transcriptional activity in a context-dependent manner—acting either as a repressor or co-activator of estrogen-responsive genes—highlighting its complex interplay with endocrine signaling. In advanced ER⁺ breast cancers, persistent TGF-β signaling coupled with dysregulated SMAD4 expression is strongly associated with invasiveness and metastasis, reinforcing its role in endocrine therapy resistance [21]. In our study, IVM did not alter SMAD4 protein levels, indicating that SMAD4 expression was preserved during treatment. This suggests that therapeutic strategies aimed at restoring SMAD4 function while simultaneously inhibiting TGF-β signaling may provide a promising avenue to overcome resistance and improve clinical outcomes. Although the present study could not directly test whether IVM inhibits TGF‑β–induced EMT, we plan to investigate the effects of IVM on TGF‑β–driven EMT and related SMAD‑dependent functional outcomes in future work.

Taken together, IVM appears to act at critical nodes where ER, HER2, and TGF‑β pathways converge. By suppressing ER and HER2 expression, IVM reduces proliferative signaling. Concurrently, IVM inhibits pERK and pSMAD2—key downstream mediators that integrate signals from ER, HER2, and TGF‑β networks. Because ERK and SMAD function as shared hubs that coordinate transcriptional programs associated with endocrine resistance, our finding suggests that IVM disrupts not only individual pathways but also the crosstalk that sustains resistant phenotypes.

Regarding the safety profile of IVM, our findings report that IVM has an IC_50_ value of about 11 µM in MCF-7 cells, consistent with earlier reports for this cell line [4]. Additionally, Dou et al observed an IC_50_ value of around 36 µM in MCF-10A, a non-tumorigenic cell line [4]. As a result, the selectivity index for IVM is approximately more than threefold, falling within an acceptable range for anti-cancer agents [42], and aligns with our study showing IVM’s selectivity indexes in normal human fibroblasts versus breast cancer cells were also greater than threefold. The combination treatment of IVM and 4-OHT (active metabolite of tamoxifen) also exhibited the selective anti-proliferation effect in breast cancer over normal cells. Micromolar-range IC₅₀ values are commonly observed during the preliminary study of anticancer lead identification before medicinal chemistry optimization. The important toxicity of ivermectin is neurotoxicity, which has rarely occurred in approved doses but may be observed with overdose [43]. Ivermectin exhibits poor penetration across the vertebrate blood–brain barrier due to active efflux by P‑glycoprotein transporters [43]. No central nervous system toxicity has been observed at doses up to ten times the highest FDA‑approved dose of 200 µg/kg. However, these safety data were derived from a relatively small cohort of 68 participants [44]. Although IC_50_ values in the micromolar range pose certain challenges and limitations, this study is intended to provide a mechanistic foundation for future research, including combination therapies, targeted drug delivery, or structural modifications to enhance potency within clinically relevant exposure levels.

## Conclusion

IVM suppresses ER and HER2 signaling, as well as pSMAD2 and pERK, which are key mediators of the ER, HER2, and TGF-β pathways. By targeting these molecules, IVM disrupts the crosstalk between signaling networks implicated in endocrine resistance. These findings suggest that IVM may play a role in both preventing the onset of endocrine resistance in ER⁺ breast cancer and improving therapeutic outcomes in antihormonal-resistant breast cancer.

## Supporting information

S1 Fig IVM decreased the viability of breast cancer cells in a time-dependent manner: MCF-7, MCF-7/LCC2, MCF-7/LCC9. MCF-7, MCF-7/LCC2, and MCF-7/LCC9 cells were treated with 3.12, 6.25, and 12.5 µM of IVM for 24, 48, and 72 h. MTT assays were performed to measure cell viability. The graphs displayed the mean ± SEM. **p < 0.01, **p < 0.001 and ***p < 0.0001 compared with cell viability at 24 h.
(TIF)

S2 Fig The combined treatment of IVM and 4-OHT killed breast cancer cells more than normal cells: MCF-7, CRL-1474. MCF-7 and CRL-1474 cells were treated with IVM at 3 and 9 µM and 4-OHT at 2.5–10 µM for 24 h. MTT assays were performed to measure cell viability. The graphs displayed the mean ± SEM. **p < 0.01 and ***p < 0.0001 compared with cell viability of CRL-1474 cells at the same concentration.
(TIF)

S3 Fig IVM decreased *pS2* mRNA expression in ER⁺ breast cancer cell lines: MCF-7. MCF-7 cells were treated with IVM at 8, 10, and 13 µM for 24 h. mRNA expression of the *pS2* gene (ER-target gene) was determined by qPCR analysis. The graphs showed the mean ± SEM. *p < 0.05 and **p < 0.01 *versus* the non-treatment control (n = 3).
(TIF)

S4 Fig Effect of IVM on SMAD4, SMAD3, and PAK-1 in ER-positive and endocrine-resistant cell lines: MCF-7, MCF-7/LCC2, MCF-7/LCC9. **(A)** SMAD4, SMAD3, and PAK-1 protein levels were measured by Western blot after treating cells with various concentrations of IVM for 24 h. The graphs depicted the inhibitory effect of IVM on the protein levels of **(B-D)** SMAD4, **(E–G)** pSMAD3/SMAD3, and **(H–J)** pPAK-1/PAK-1 in MCF-7, MCF-7/LCC2, and MCF-7/LCC9 cells, respectively. The data were presented as mean ± SEM. *p < 0.05 and **p < 0.01 when compared to the non-treatment control (n = 3).
(TIF)

S5 Fig IVM inhibited estrogen-induced cell proliferation only in ER⁺ breast cancer cell lines: MCF-7, T-47D, MCF-7/LCC2, MCF-7/LCC9. MTT assay measured cell viability of breast cancer cells: All cells were treated with (+E_2_) or without (−E_2_) 17β-estradiol at physiologic level (10 nM E_2_) in the presence of nontoxic concentrations of IVM (1, 3, 5 μM) for 5 days. The results for **(A)** MCF-7 and **(B)** T-47D cells are presented as concentration-response curves showing mean ± SEM. Grey asterisks indicate significant differences compared to the group at the same IVM’s concentration without E_2_ treatment. The results for **(C)** MCF-7/LCC2 and **(D)** MCF-7/LCC9 cells are presented as graphs showing the mean ± SEM. Blue and red asterisks indicate significant differences compared to non-treatment groups without (−E2) and with (+E2), respectively. *p < 0.05, **p < 0.01, ***p < 0.001, ****p < 0.0001 *versus* the non-treatment control (n = 3).
(TIF)

S6 Fig The combined treatment of IVM and 4-OHT further reduced ERα and HER2 levels in ER-positive and endocrine-resistant breast cancer cell lines: T-47D, T-47D Tam1, T47D-182R1. **(A)** Western blots were performed in three T-47D breast cancer cell lines after the treatments with concentrations at IC_25_ values of IVM and/or 4-OHT for 24 h. The fold change in the protein level of ERα was assessed in **(B)** T-47D, **(C)** T-47D Tam1 cells, and HER2 in **(D)** T-47D, **(E)** T-47D Tam1, and **(F)** T47D-182R1 cells. The graphs displayed the mean ± SEM. *p < 0.05 and **p < 0.01 compared with non-treatment.
(TIF)

S7 Fig Combination effect of 4-OHT and IVM in ER-positive and endocrine-resistant breast cancer cell lines: MCF-7, MCF-7/LCC2, MCF-7/LCC9. **(A)** MCF-7, **(B)** MCF-7/LCC2, and **(C)** MCF-7/LCC9 cells were treated with 2.5, 5, 7.5, and 10 µM of 4-OHT and/or 0.05–9 µM of IVM for 72 h. The combination index (CI) and normalized isobologram analysis were obtained using the CompuSyn program and the Chou–Talalay method. The combination analysis showed synergism (CI < 1), additive (CI = 1), and antagonism (CI > 1). The normalized isobologram demonstrated a drug interaction between 4-OHT and IVM. The raw data were obtained from Rujimongkon et al (2025), PLoS One 20(6): e0326742, according to PLOS One Creative Commons Attribution 4.0 International (CC BY) license.
(TIF)

S8 Fig Effect of IVM on other pathways involved in tamoxifen resistance in ER-positive and endocrine-resistant cell lines: MCF-7, MCF-7/LCC2, MCF-7/LCC9. **(A)** Protein levels were measured by Western blot after treating cells with various concentrations of IVM for 24 h. The graphs depicted the inhibitory effect of IVM on the protein levels of **(B–D)** PI3K, **(E–G)** pAKT/AKT, and **(H–J)** p-mTOR/mTOR in MCF-7, MCF-7/LCC2, and MCF-7/LCC9 cells, respectively. The data were presented as mean ± SEM. *p < 0.05 and ****p < 0.0001 when compared to the non-treatment control (n = 3).
(TIF)

S9 Fig Graphical Abstract. (TIF)

S1 Table Resistant tests-Cell viability (%) of breast cancer cell lines exposed to 6.25 µM drug for 24 hours. (DOCX)

S2 Table Selective index (SI) of IVM and 4-OHT in breast cancer cell lines after 24 hours of exposure. (DOCX)

S3 Table Inhibitory Concentration at 25 percent (IC_25_) of IVM in breast cancer cell lines after 24 hours of exposure. (DOCX)

S1 File Original uncropped images of all blots. (DOCX)

S2 File Raw data and statistical analysis of all figures. (DOCX)
