# Supplementary material for: Ivermectin inhibits ER, HER2, and TGF-β pathways in ER-positive and endocrine-resistant breast cancer cells
Source: PLoS One. 2026 Apr 30;21(4):e0348260. doi: 10.1371/journal.pone.0348260 (PMC13132456; doi:10.1371/journal.pone.0348260)
Supplement: S1 Table — (DOCX) [file pone.0348260.s010.docx]

**Supplementary Tables**

**S2 Table**: Selective index (SI) of IVM and 4-OHT in breast cancer cell lines after 24 hours of exposure

| **Type of cell line** | **Cell line** | **IVM**  IC_50_ (µM) ± SEM | **Selective Index**  (IVM) | **4-OHT**  IC_50_ (µM) ± SEM | **Selective Index**  4-OHT |
| --- | --- | --- | --- | --- | --- |
| Endocrine-sensitive | MCF-7 | 11.44 ± 0.25 | 3.64 | 6.31 ± 0.53 * | 5.14 |
| Tamoxifen-resistance | MCF-7/LCC2 | 9.62 ± 0.42 | 4.33 | 11.62 ± 0.12 * | 2.79 |
| Fulvestrant-resistance | MCF-7/LCC9 | 9.28 ± 0.18 | 4.49 | 10.65 ± 0.91 * | 3.05 |
| Endocrine-sensitive | T-47D | 10.29 ± 0.20 | 4.05 | 8.89 ± 0.23 | 3.65 |
| Tamoxifen-resistance | T-47D Tam1 | 10.27 ± 0.44 | 4.06 | 9.82 ± 0.11 | 3.30 |
| Fulvestrant-resistance | T47D-182R1 | 10.32 ± 0.14 | 4.04 | 10.34 ± 0.09 | 3.14 |
| Human normal skin fibroblast | CRL-1474 | 41.67 ± 0.35 | - | 32.45 ± 0.71 | - |

*Reference data obtained from Rujimonkon et al (2025), PLoS One 20(6): e0326742
