# Supplementary material for: Ivermectin inhibits ER, HER2, and TGF-β pathways in ER-positive and endocrine-resistant breast cancer cells
Source: PLoS One. 2026 Apr 30;21(4):e0348260. doi: 10.1371/journal.pone.0348260 (PMC13132456; doi:10.1371/journal.pone.0348260)
Supplement: S3 Table — (DOCX) [file pone.0348260.s012.docx]

**Supplementary Data**

**Western blot detection**

**Figure 1A, 1B, 1E, 1K**: MCF-7

**
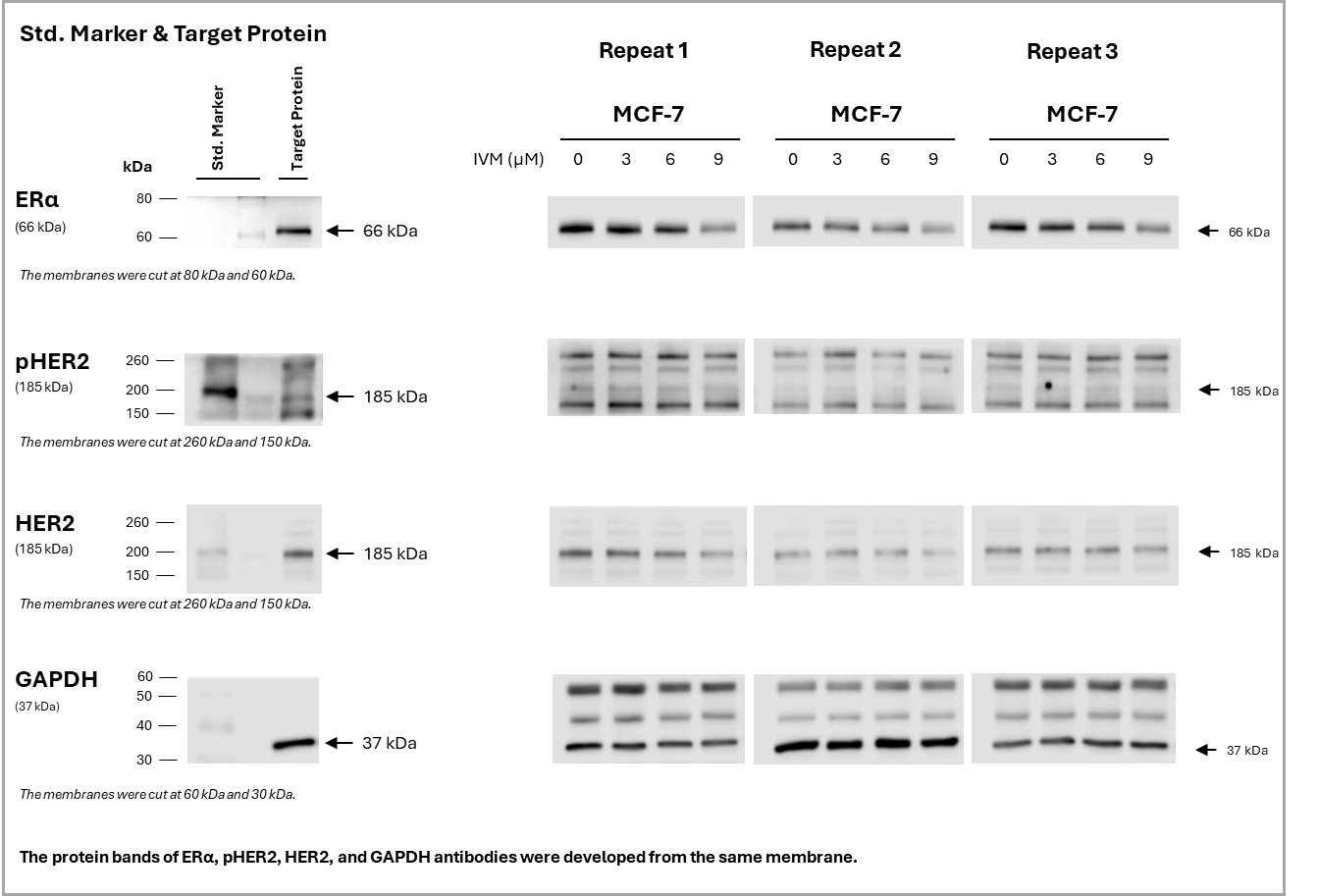
**

Note: Repeat 1, Repeat 2, and Repeat 3 used the same membrane as shown in the supplementary material for Figures S4A and S4H: MCF-7.

**Figure 1A, 1H**: MCF-7

**
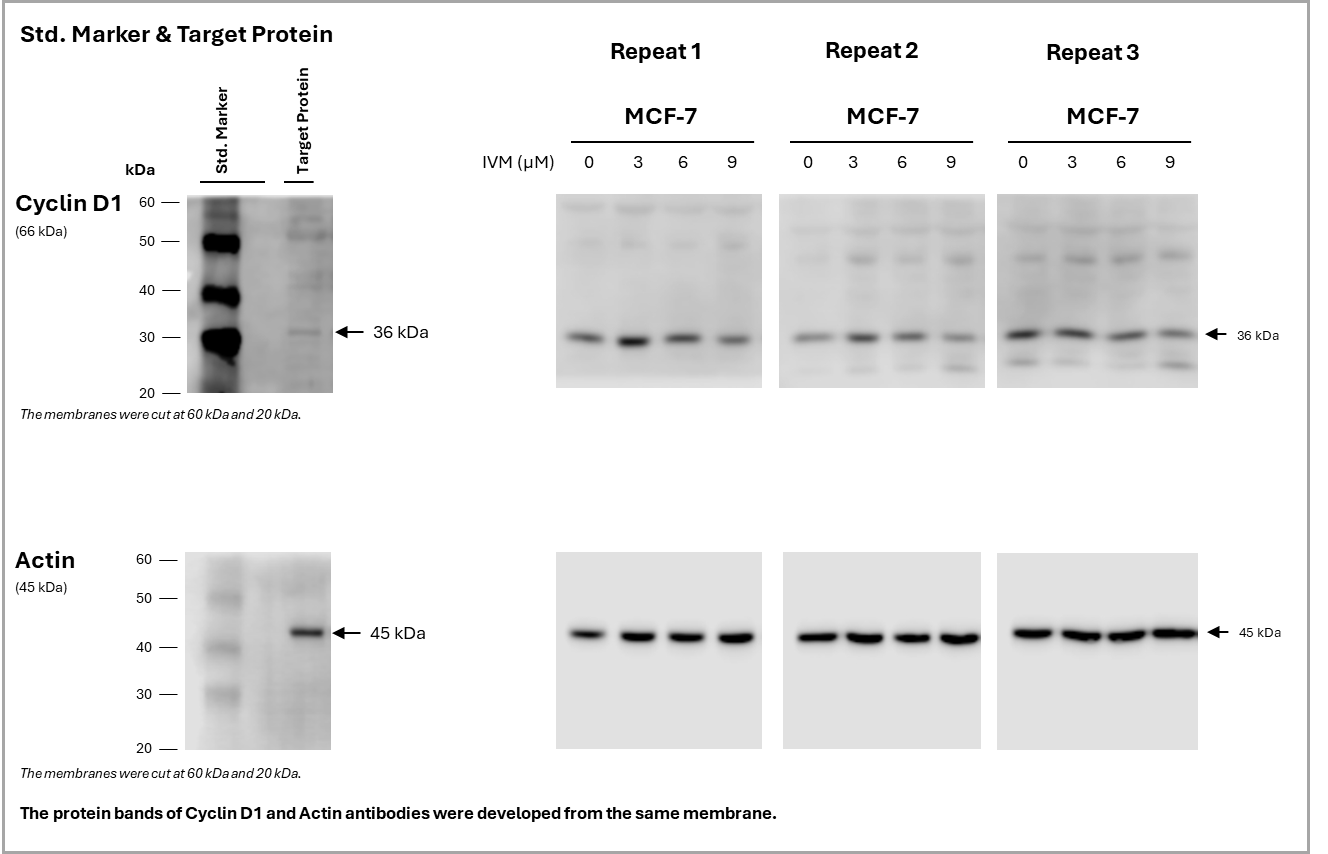
**

**Figure 1A, 1C, 1F, 1L**: MCF-7/LCC2

**
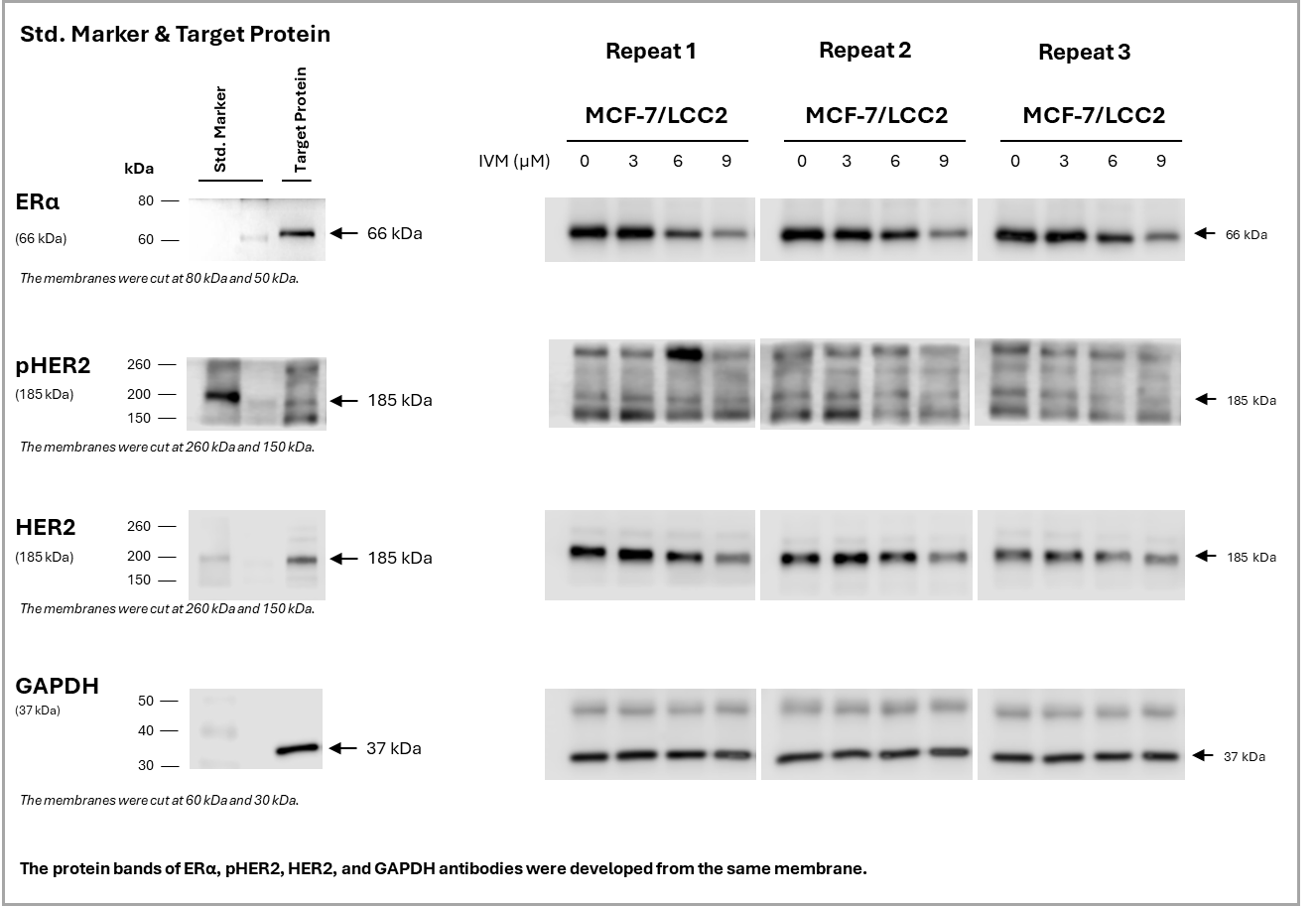
**

Note: Repeat 1, Repeat 2, and Repeat 3 used the same membrane as shown in the supplementary material for Figure S4A, S4I: MCF-7/LCC2

**Figure 1A, 1I**: MCF-7/LCC2


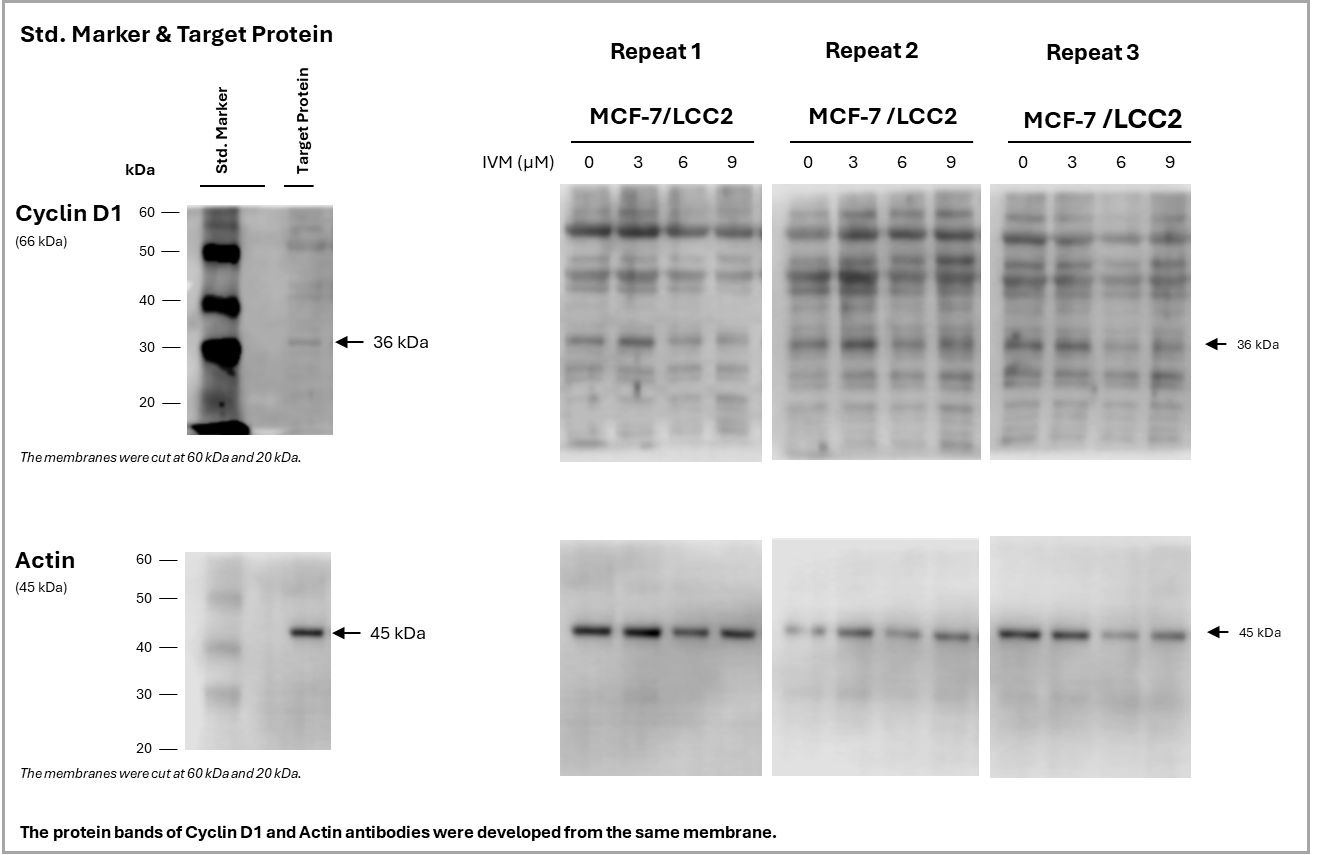


**Figure 1A, 1D, 1G**: MCF-7/LCC9

**
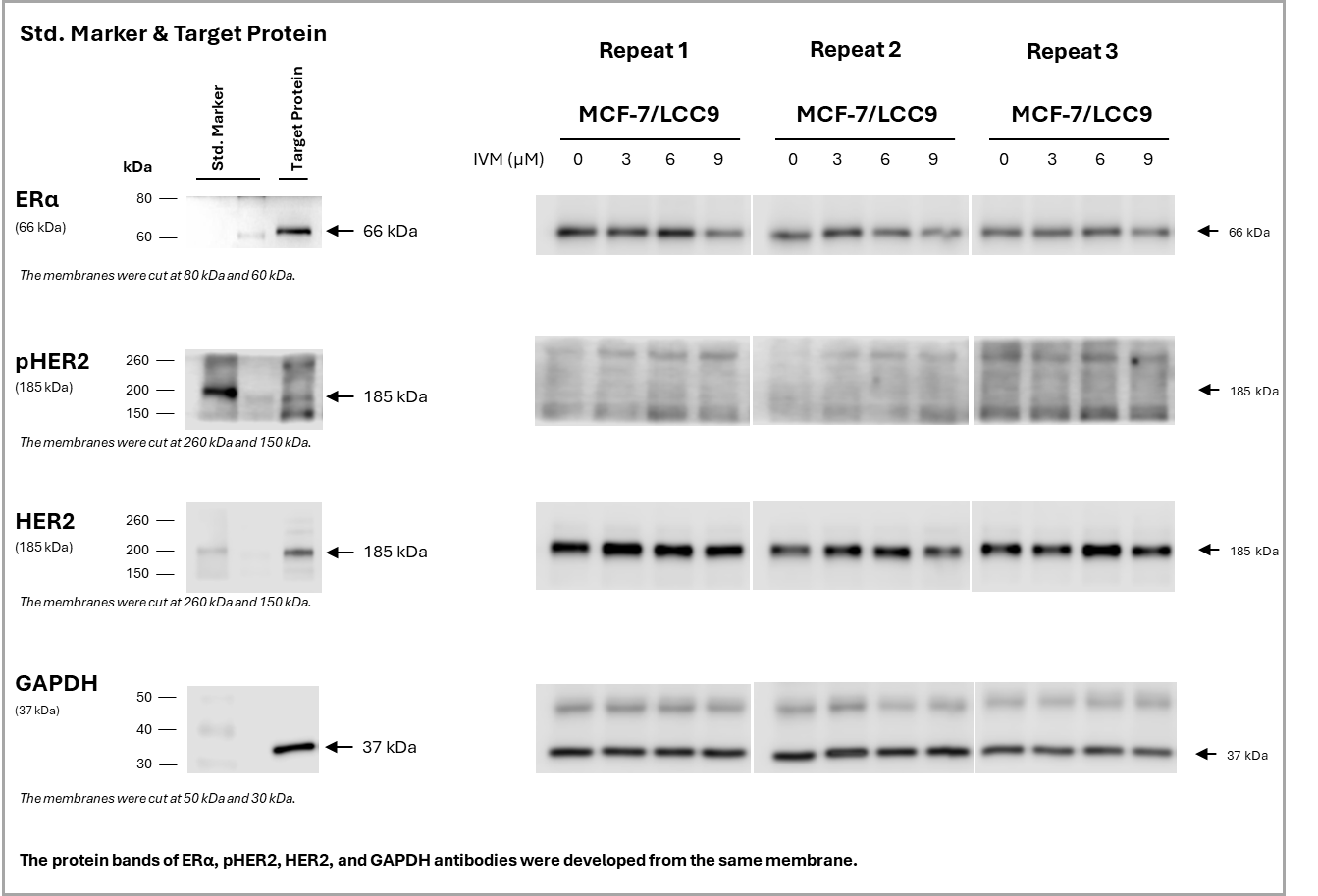
**

Note: Repeat 1, Repeat 2, and Repeat 3 used the same membrane as shown in the supplementary material for Figure S4A, S4J: MCF-7/LCC9

**Figure 1A, 1J**: MCF-7/LCC9


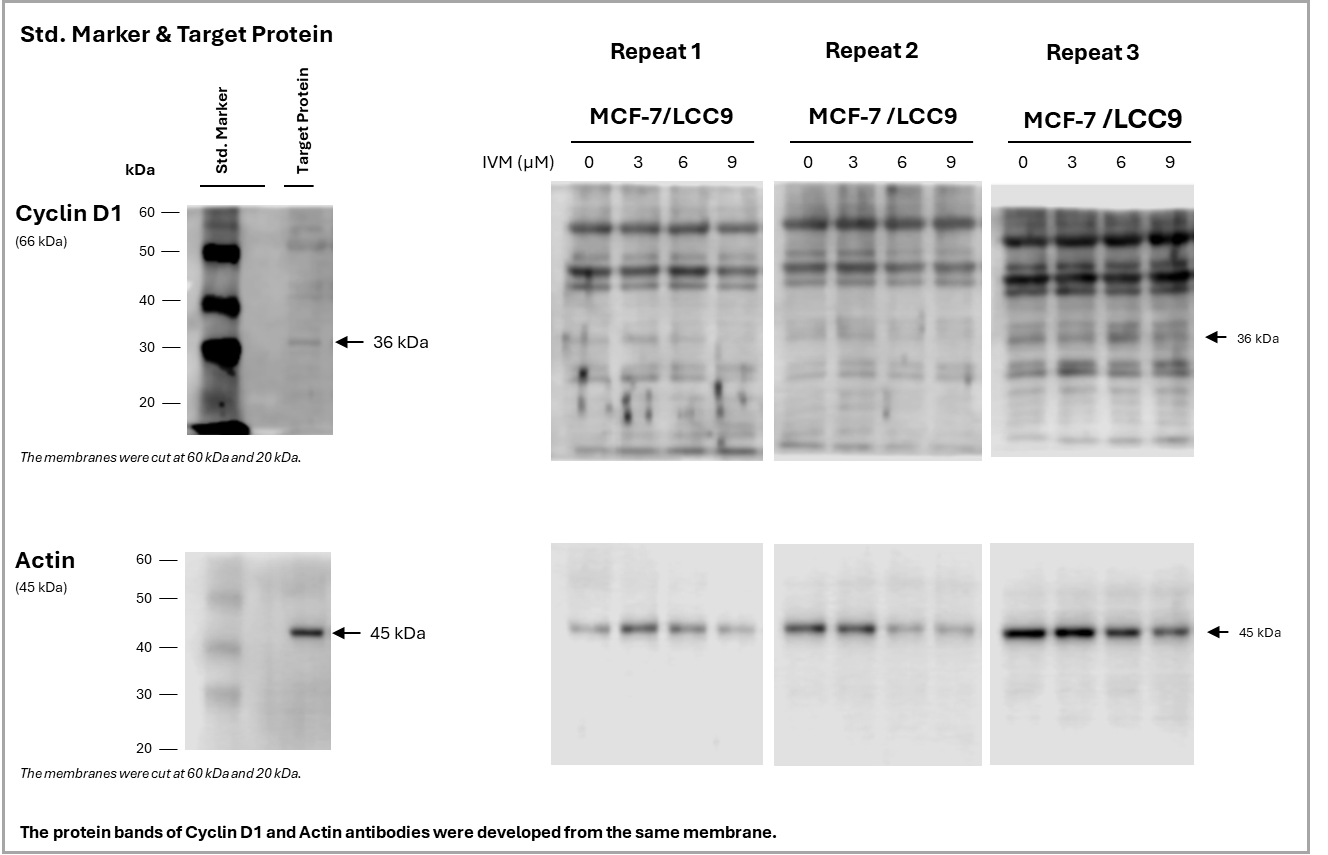


**Figure 2C, 2D, 2E**: MCF-7

**
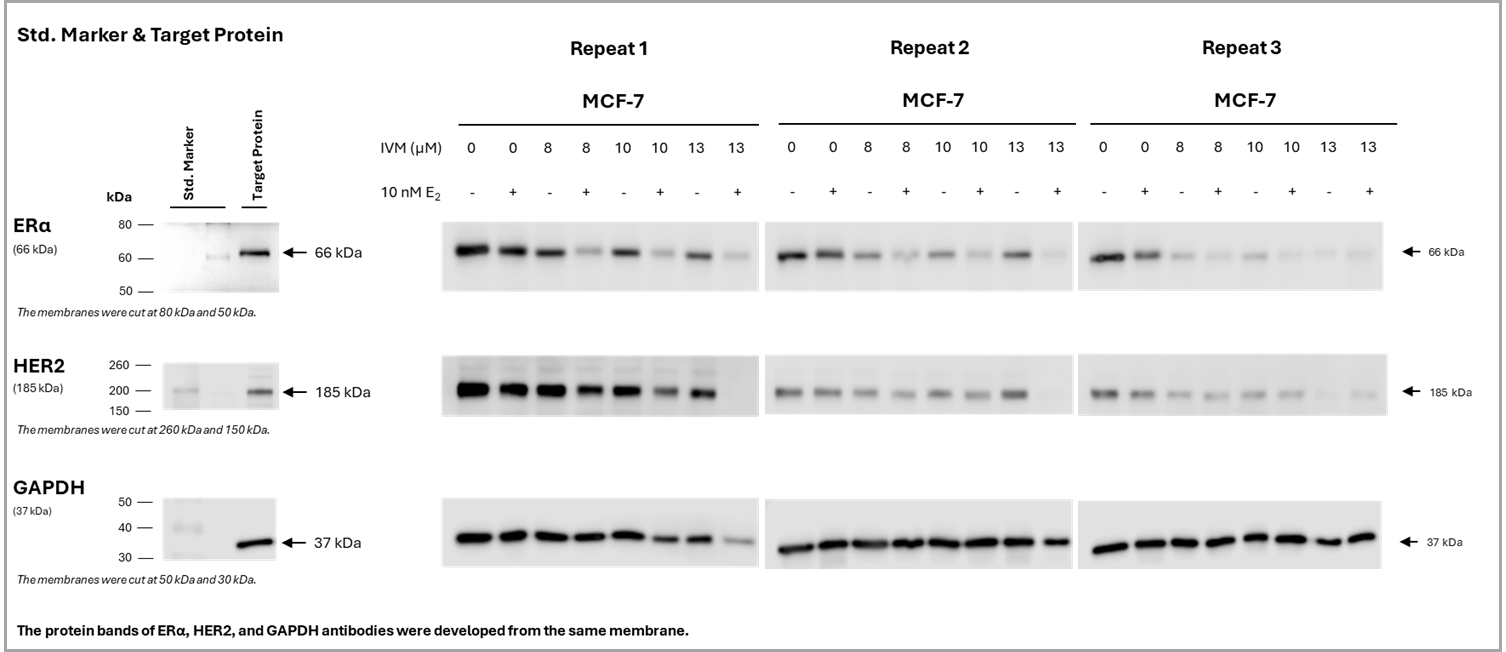
**

**Figure 3D, 3E, 3H**: MCF-7

**
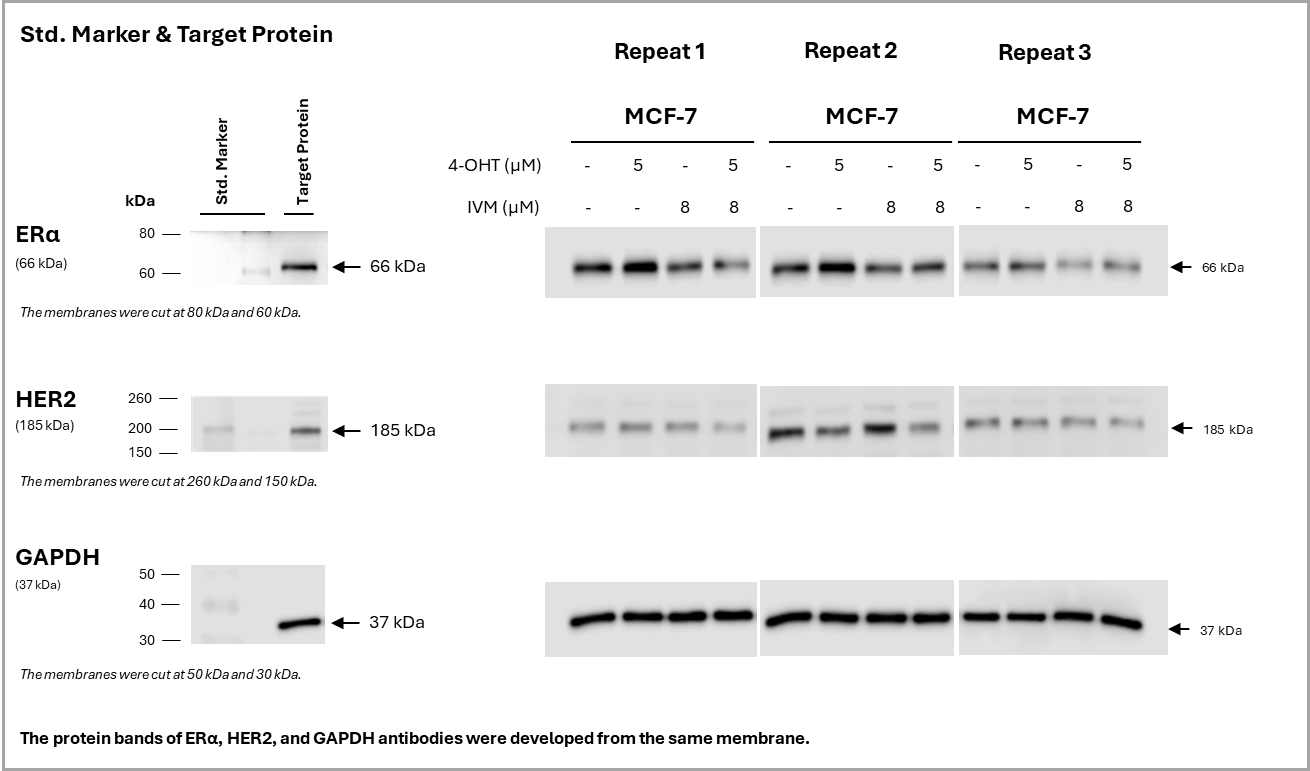
**

**Figure 3D, 3F, 3I**: MCF-7/LCC2


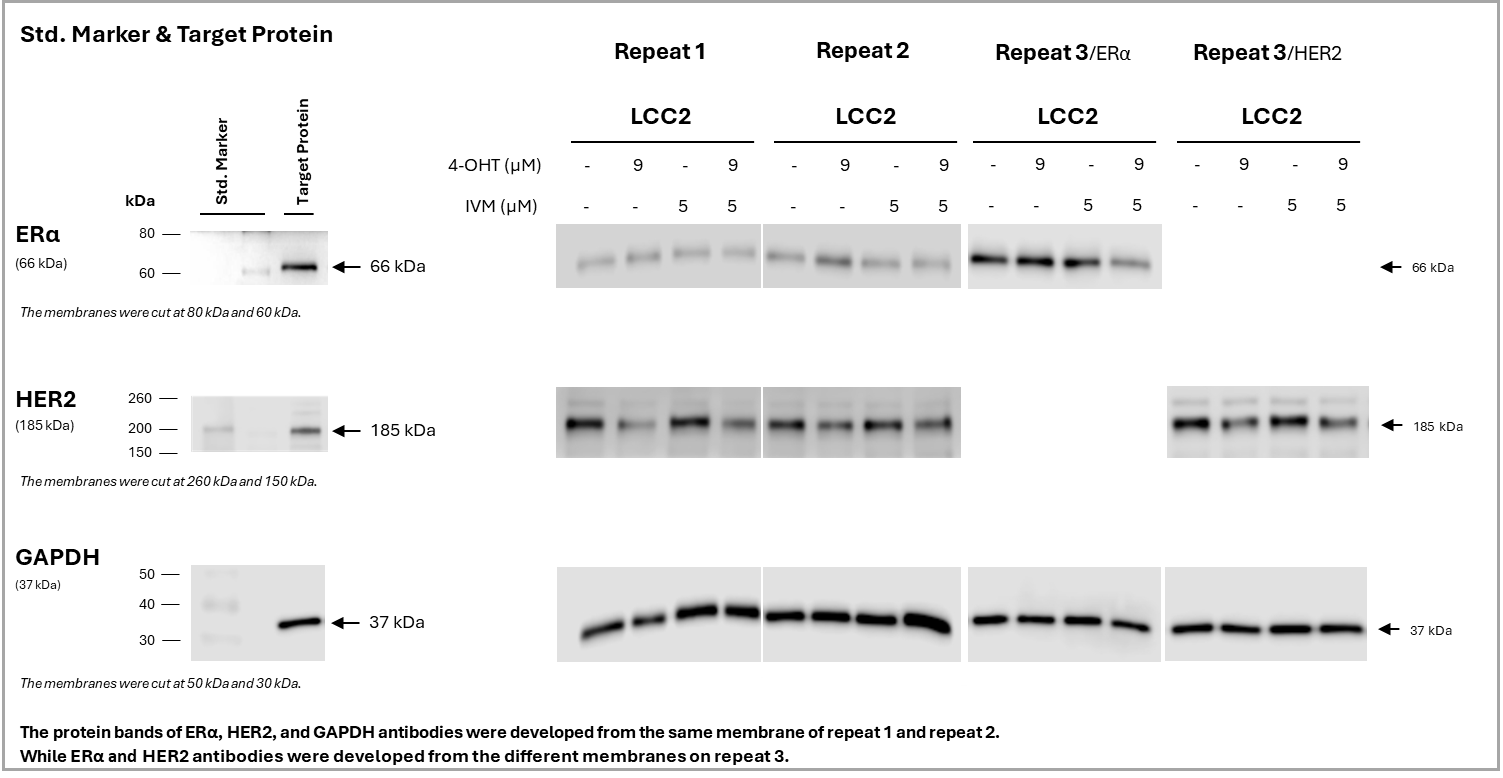


**Figure 3D, 3G, 3J**: MCF-7/LCC9


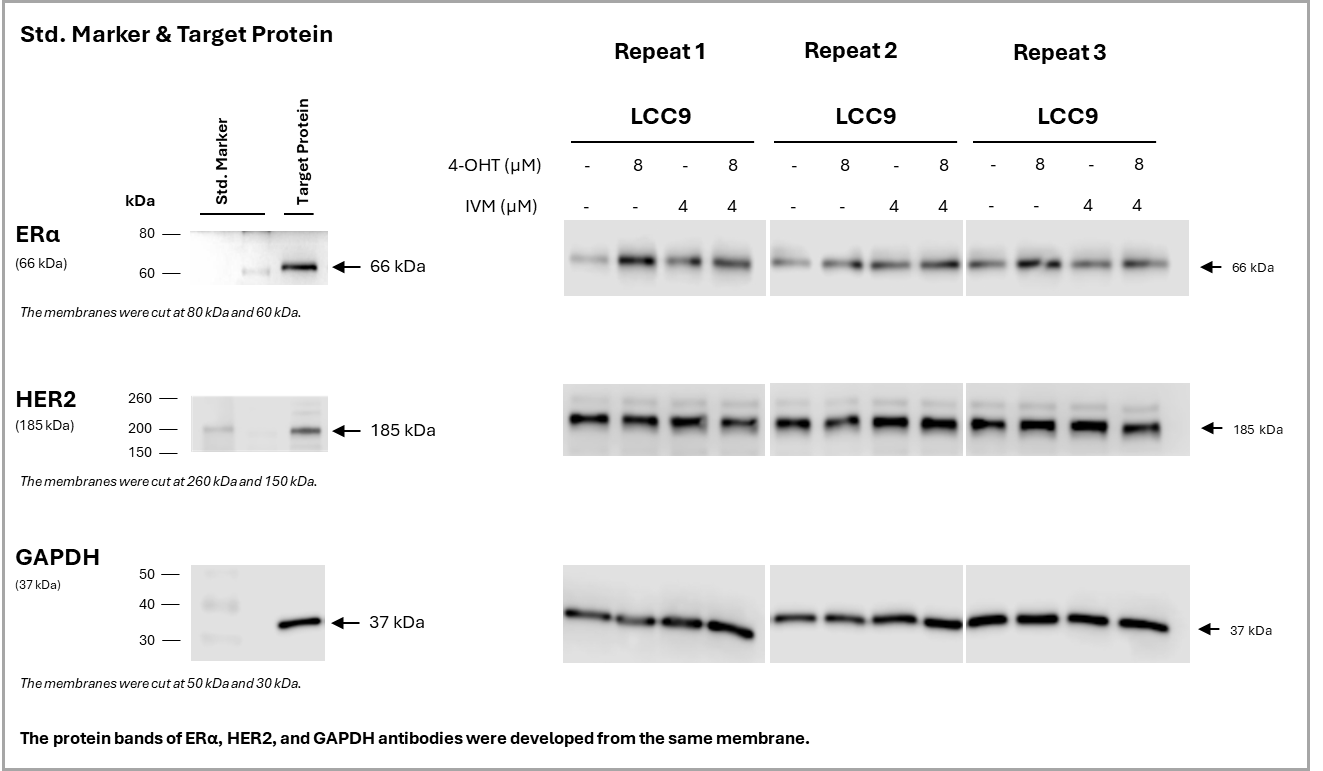


**Figure 4A, 4B, 4E**: MCF-7


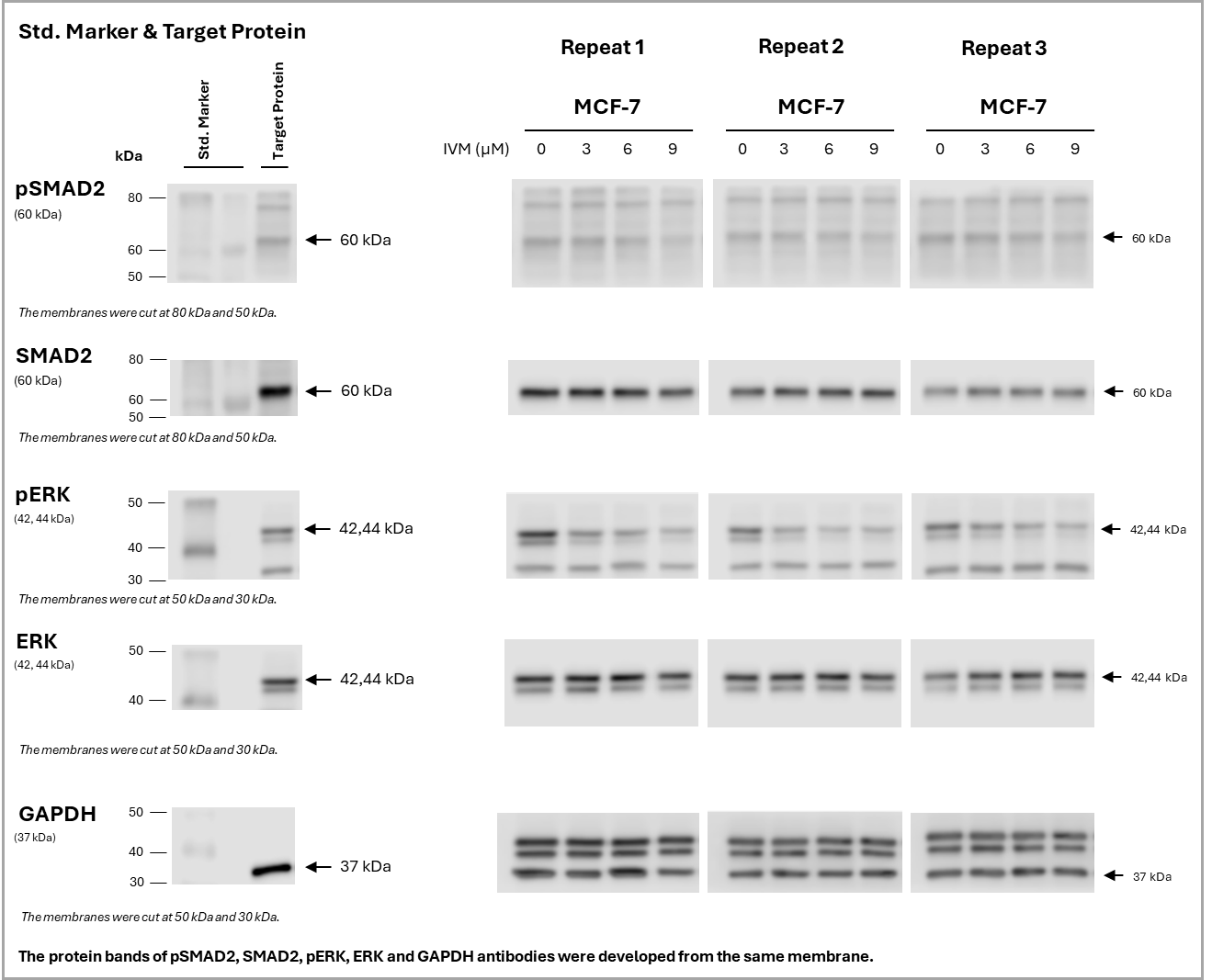


Note: Repeat 1, Repeat 2, and Repeat 3 used the same membrane as shown in the supplementary material for Figure S8A, S8B, S8H: MCF-7

**Figure 4A, 4C**: MCF-7/LCC2


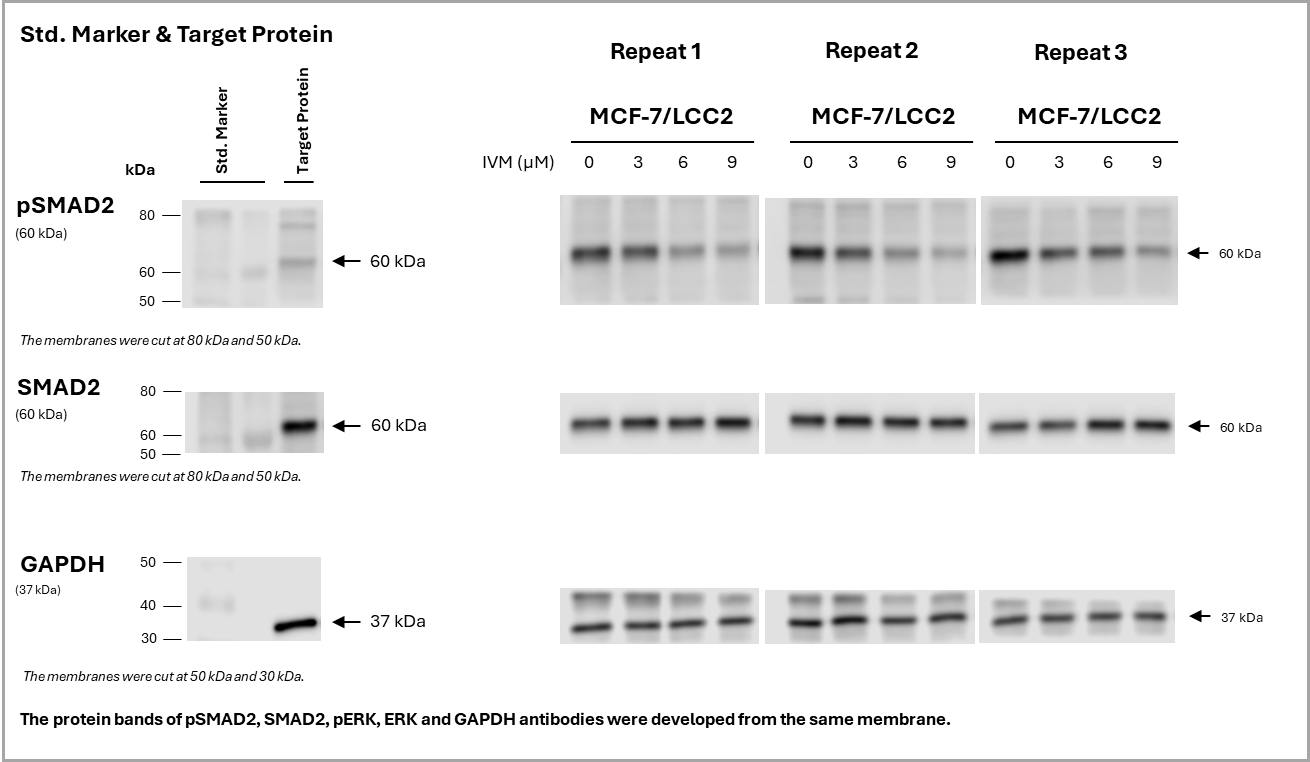


Note: Repeat 1, Repeat 2, and Repeat 3 used the same membrane as shown in the supplementary material for Figure S8A, S8C, S8I: MCF-7/LCC2

**Figure 4A, 4F**: MCF-7/LCC2


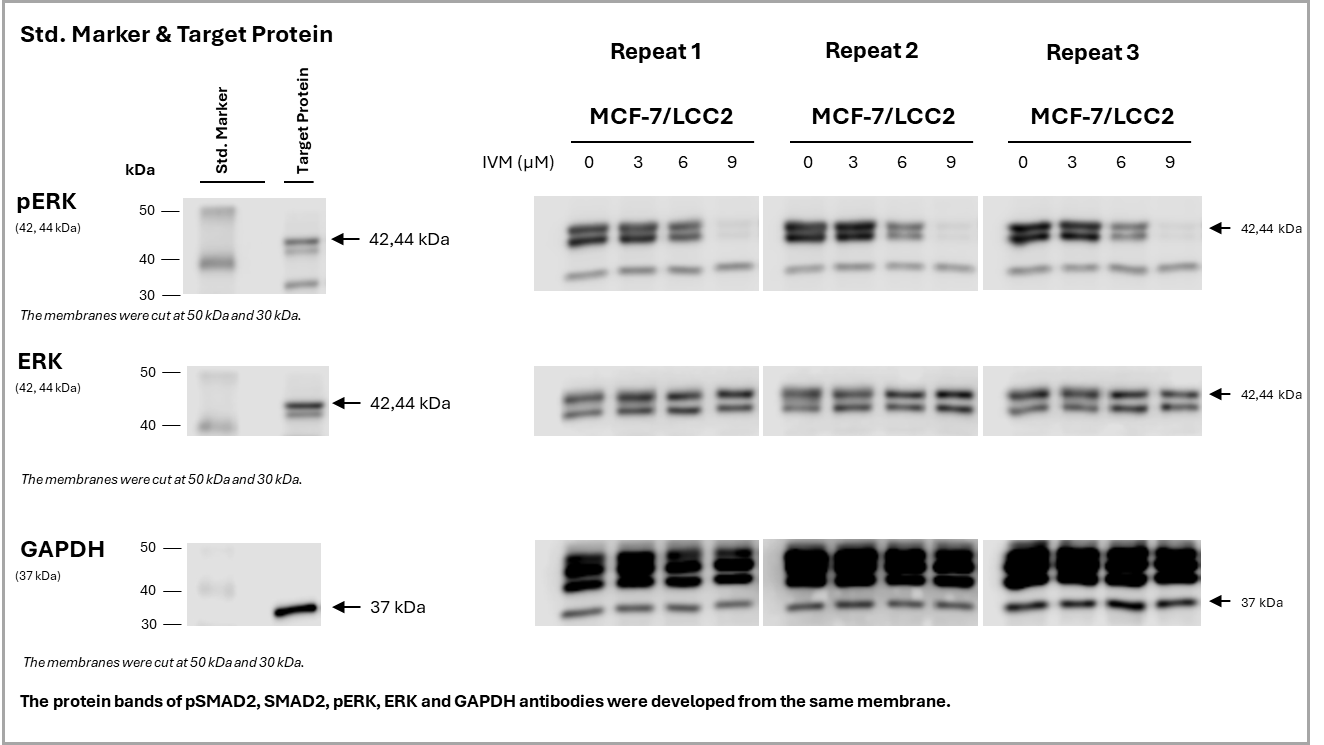


Note: Repeat 1, Repeat 2, and Repeat 3 used the same membrane as shown in the supplementary material for Figure S8A, S8F: MCF-7/LCC2

**Figure 4A, 4D**: MCF-7/LCC9


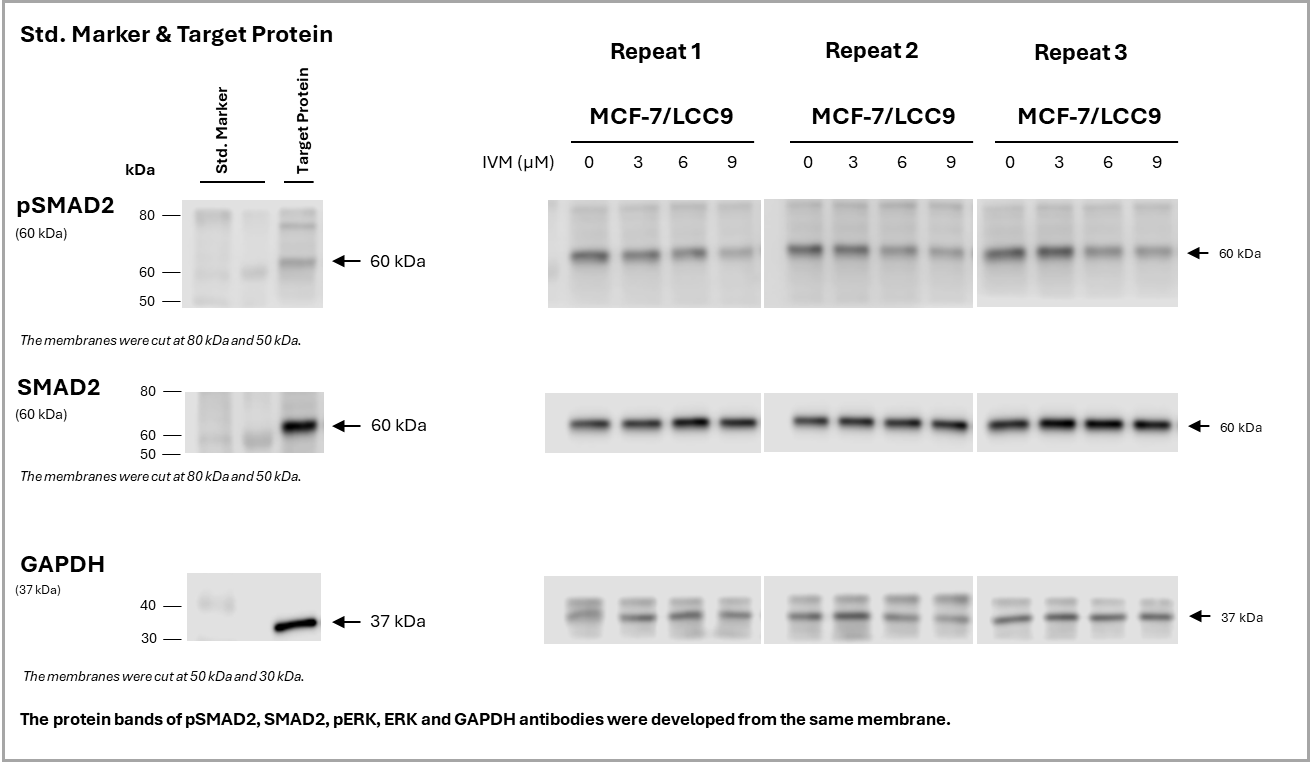


Note: Repeat 1, Repeat 2, and Repeat 3 used the same membrane as shown in the supplementary material for Figure S8A, S8D, S8J: MCF-7/LCC9

**Figure 4A, 4G**: MCF-7/LCC9
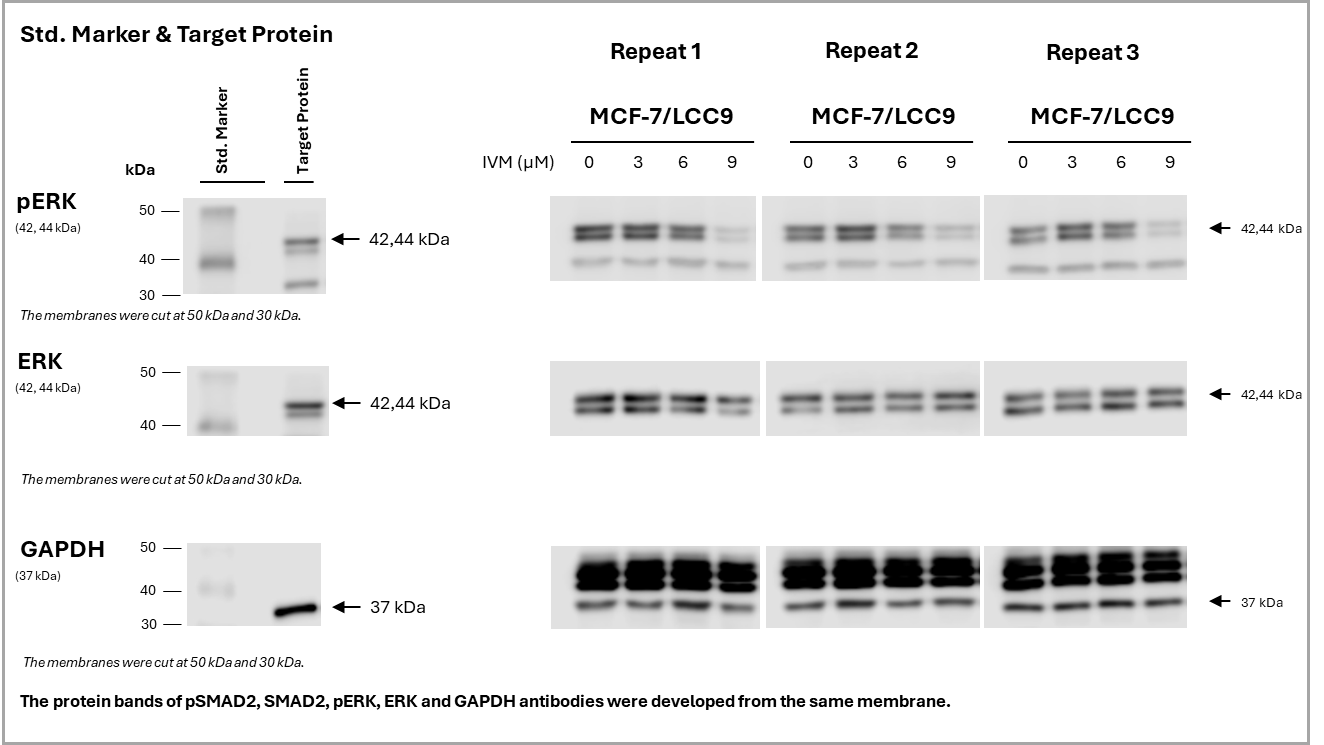


Note: Repeat 1, Repeat 2, and Repeat 3 used the same membrane as shown in the supplementary material for Figure S8A, S8G: MCF-7/LCC9

**Figure S4A, S4B, S4E**: MCF-7


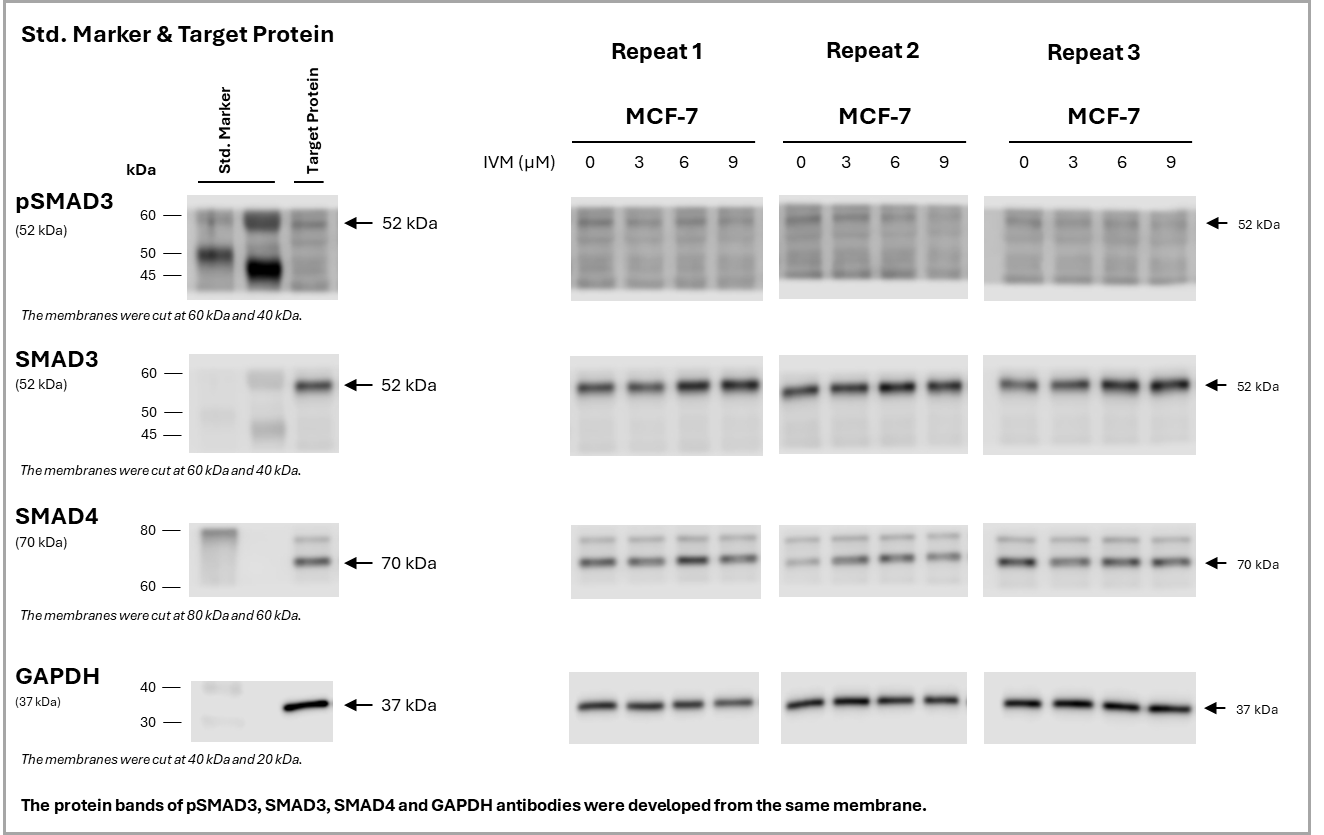


**Figure S4A, S4H**: MCF-7


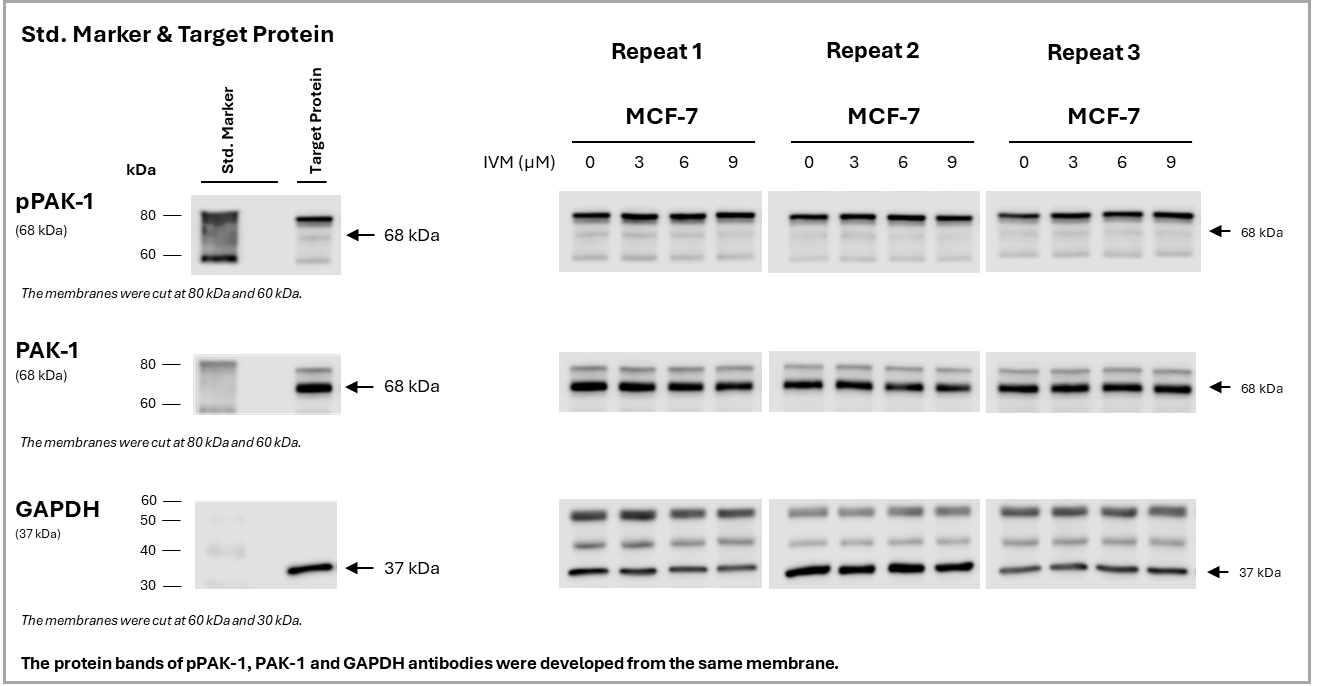


Note: Repeat 1, Repeat 2, and Repeat 3 used the same membrane as shown in the supplementary material for Figures 1A, 1B, 1E, and 1k: MCF-7.

**Figure S4A, S4C, S4F**: MCF-7/LCC2


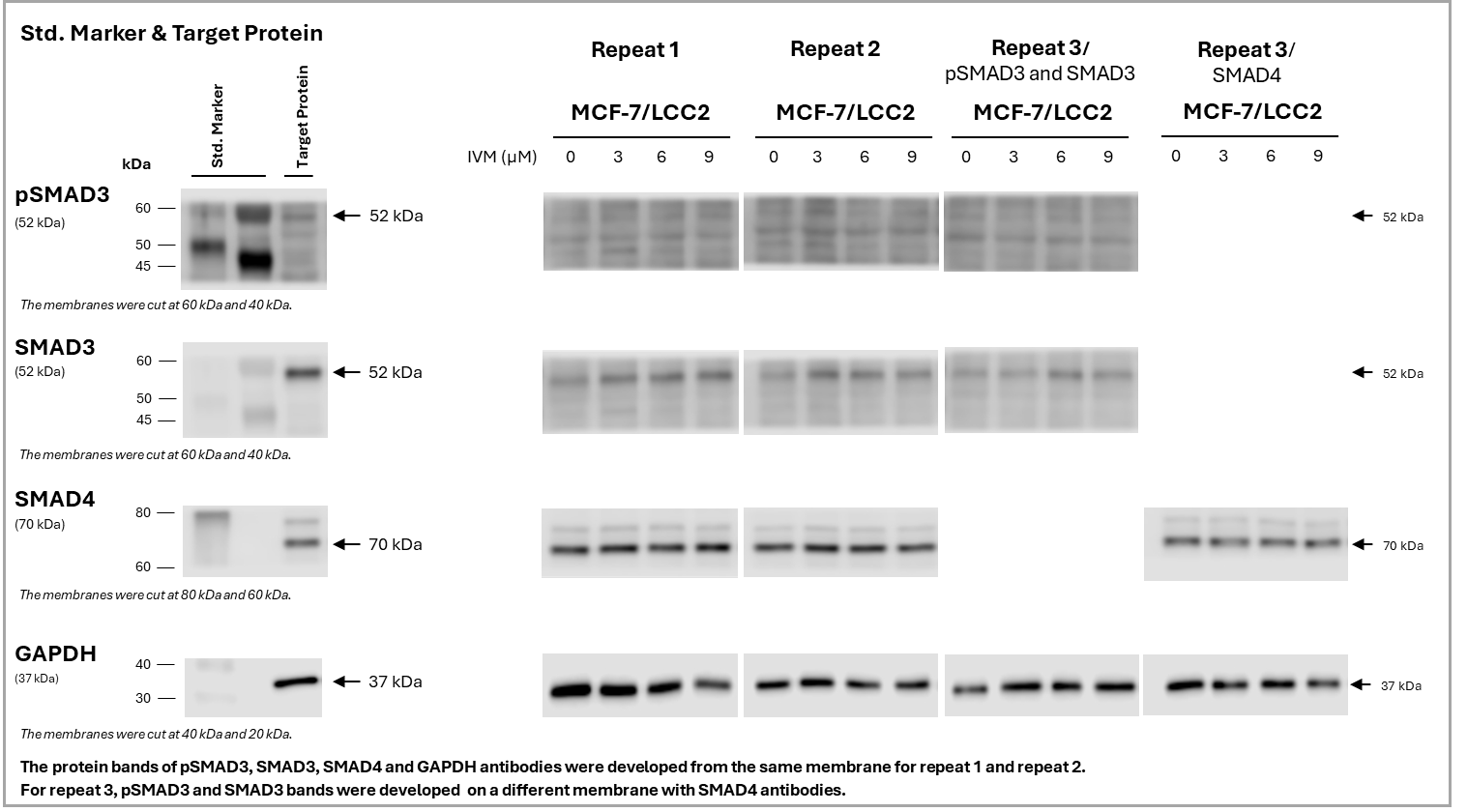


**Figure S4A, S4I**: MCF-7/LCC2


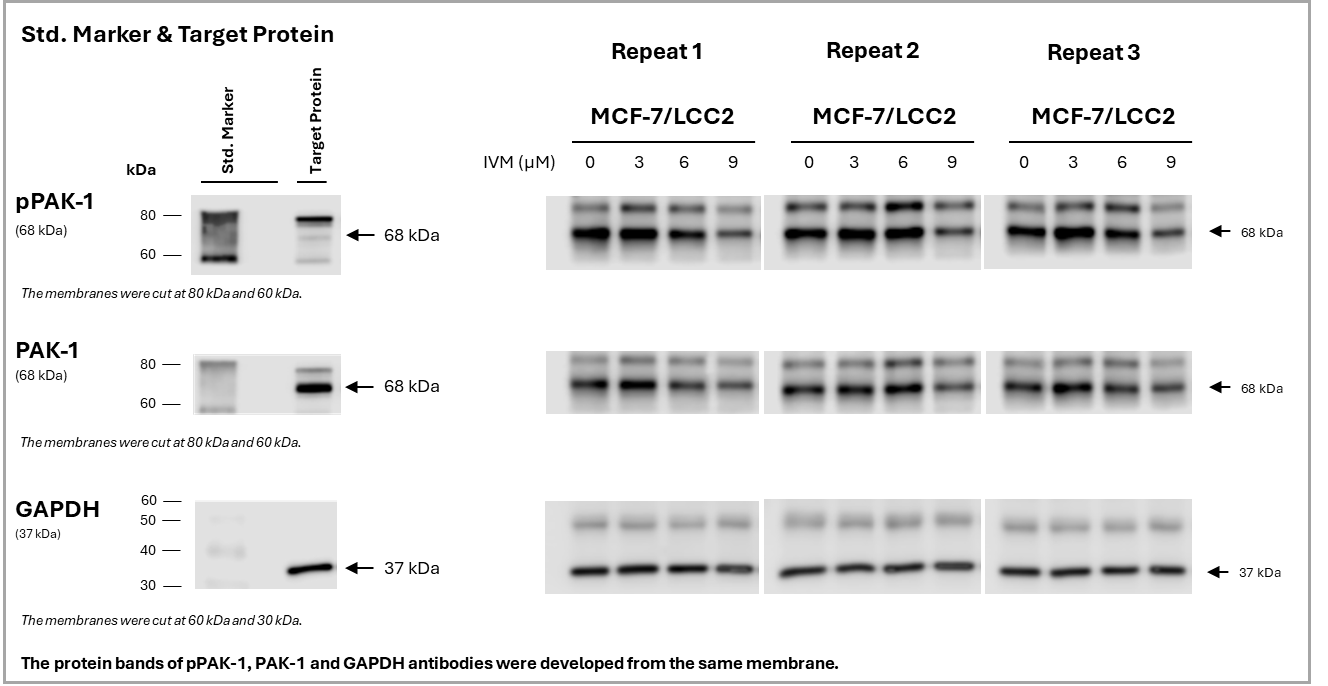


Note: Repeat 1, Repeat 2, and Repeat 3 used the same membrane as shown in the supplementary material for Figure 1A, 1C, 1F, and 1L MCF-7/LCC2

**Figure S4A, S4D, S4G**: MCF-7/LCC9


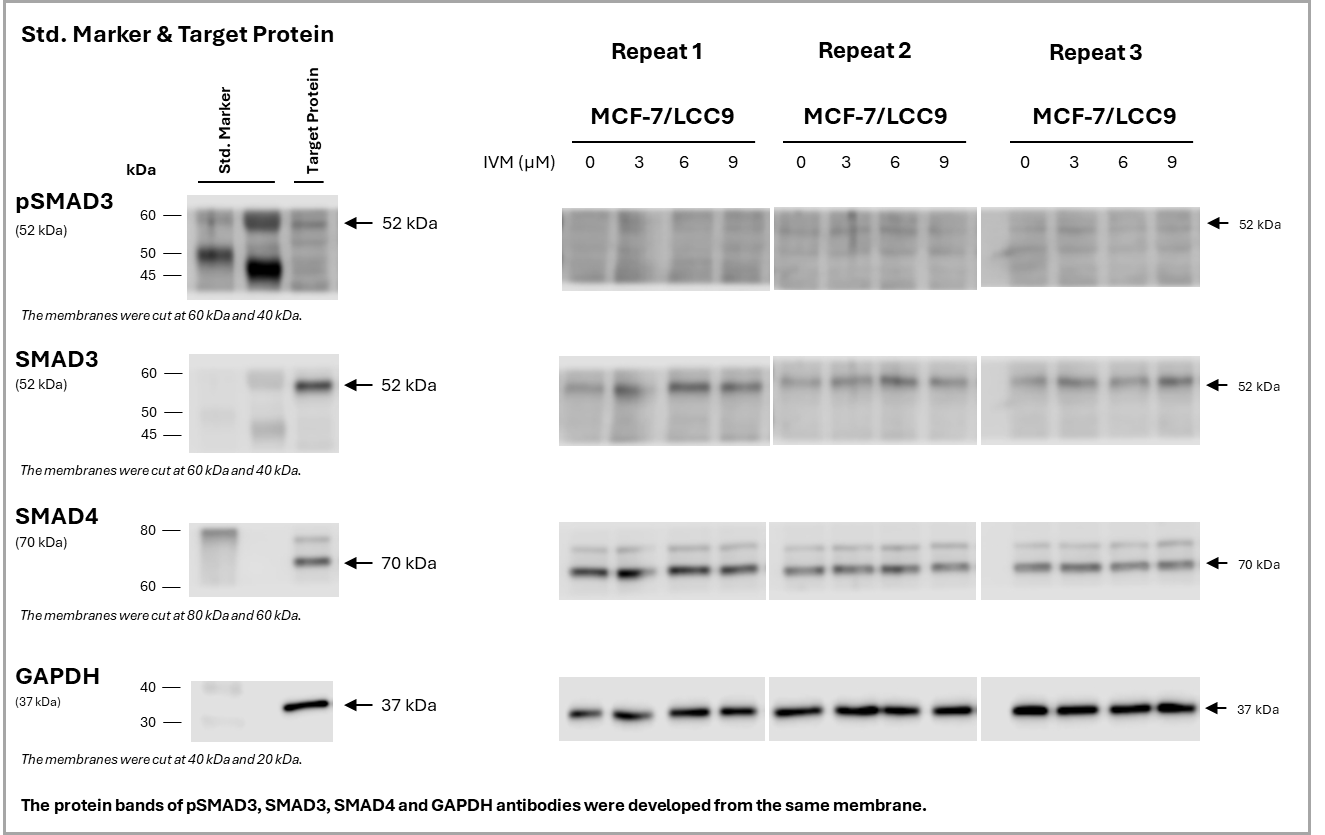


**Figure S4A, S4J**: MCF-7/LCC9


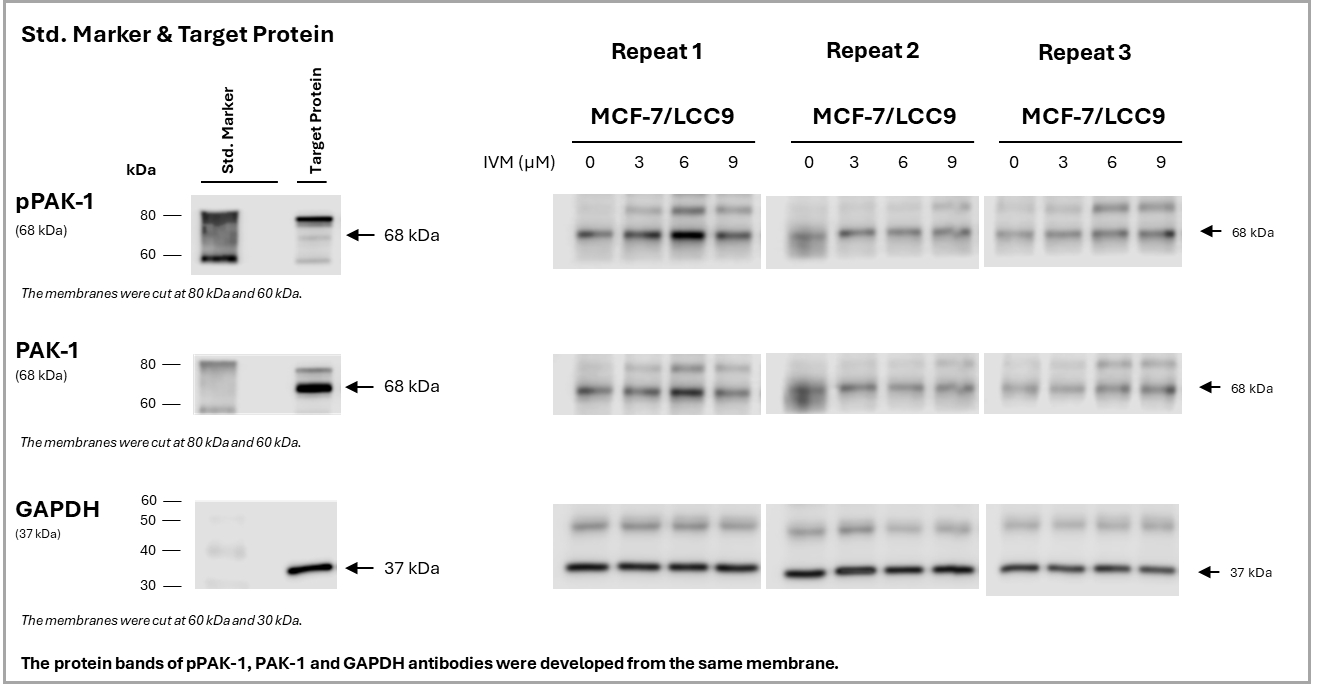


Note: Repeat 1, Repeat 2, and Repeat 3 used the same membrane as shown in the supplementary material for Figure 1A, 1D, and 1G: MCF-7/LCC9

**Figure S6A, S6B, S6D**: T-47D


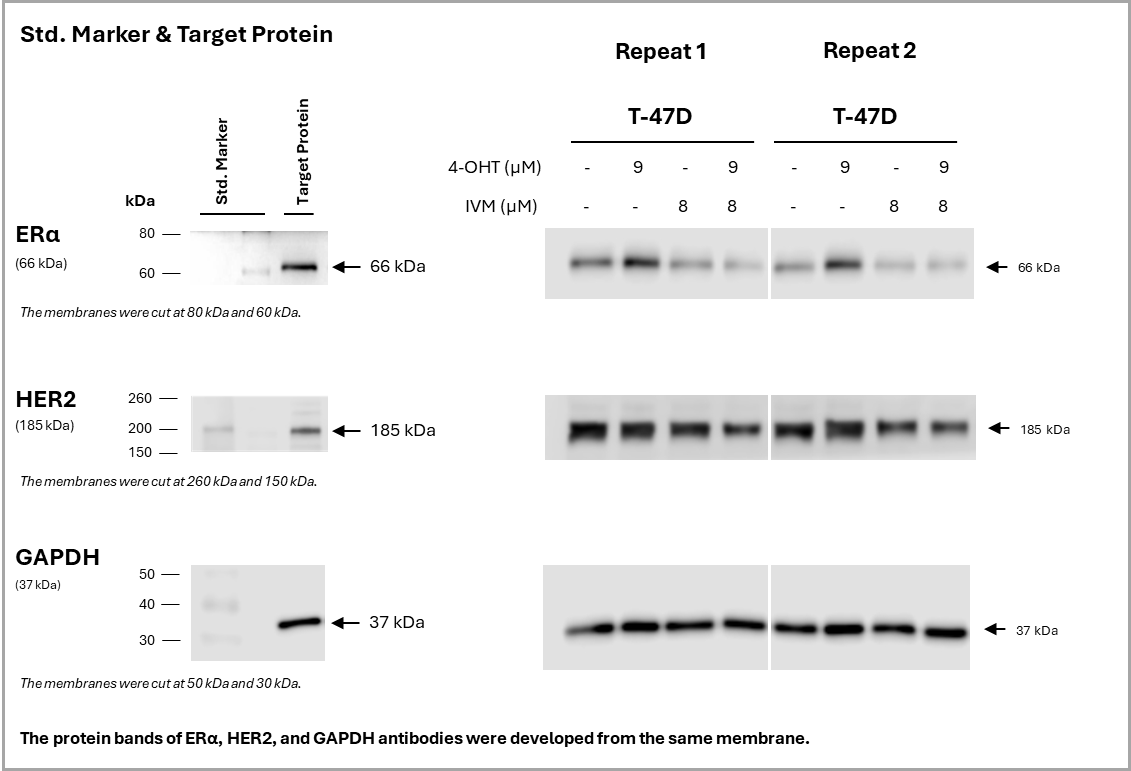


**Figure S6A, S6C, S6E**: T-47D Tam1


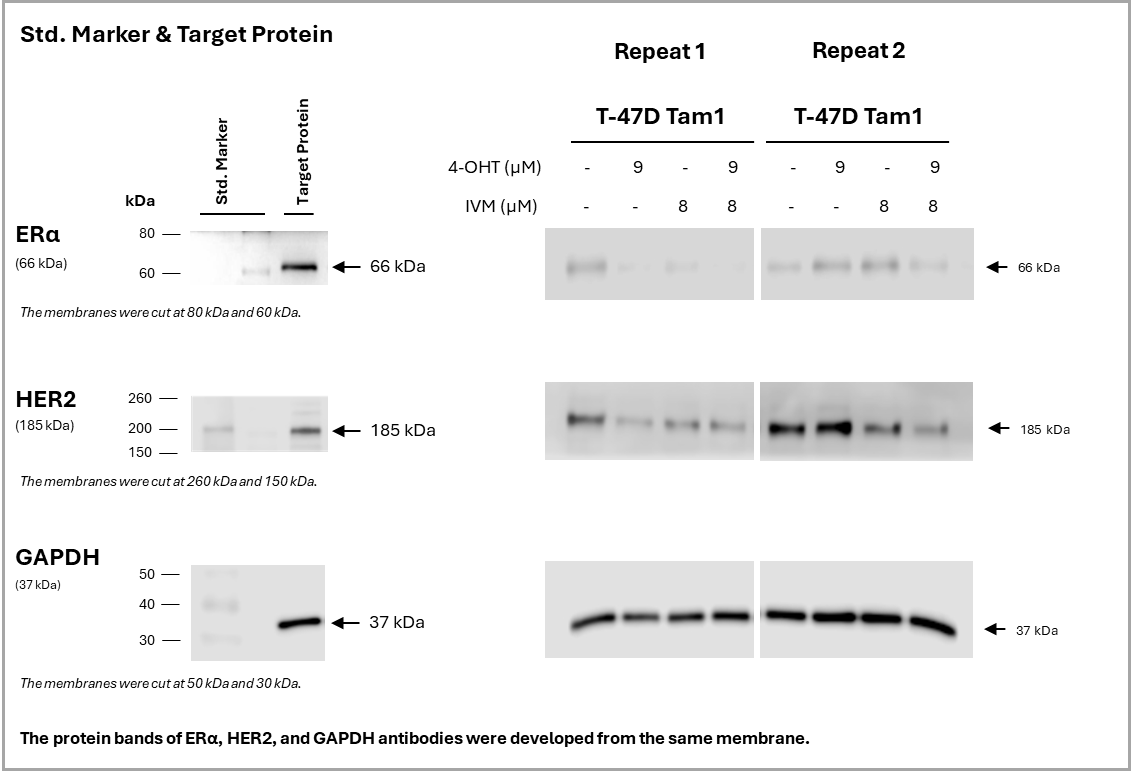


**Figure S6A, S6F**: T47D-182R1


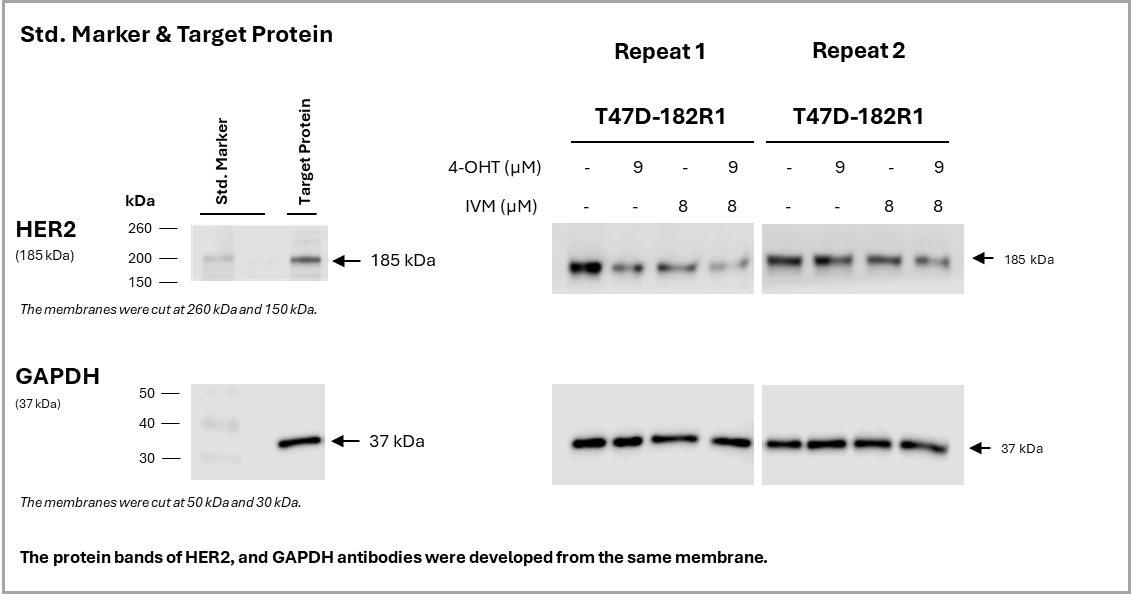


**Figure S8A, S8B, S8H**: MCF-7


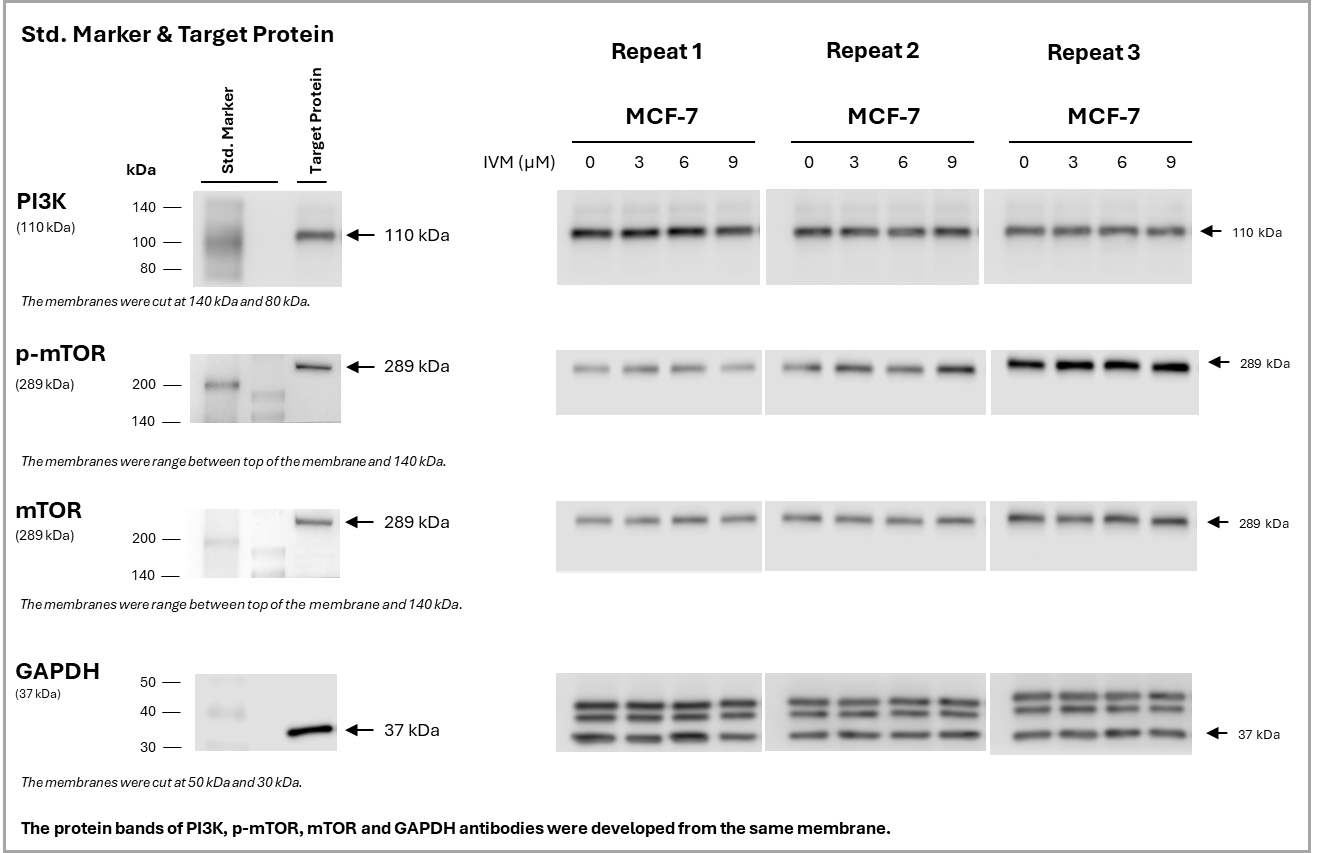


Note: Repeat 1, Repeat 2, and Repeat 3 used the same membrane as shown in the supplementary material for Figure 4A, 4B, 4E: MCF-7

**Figure S8A, S8E**: MCF-7


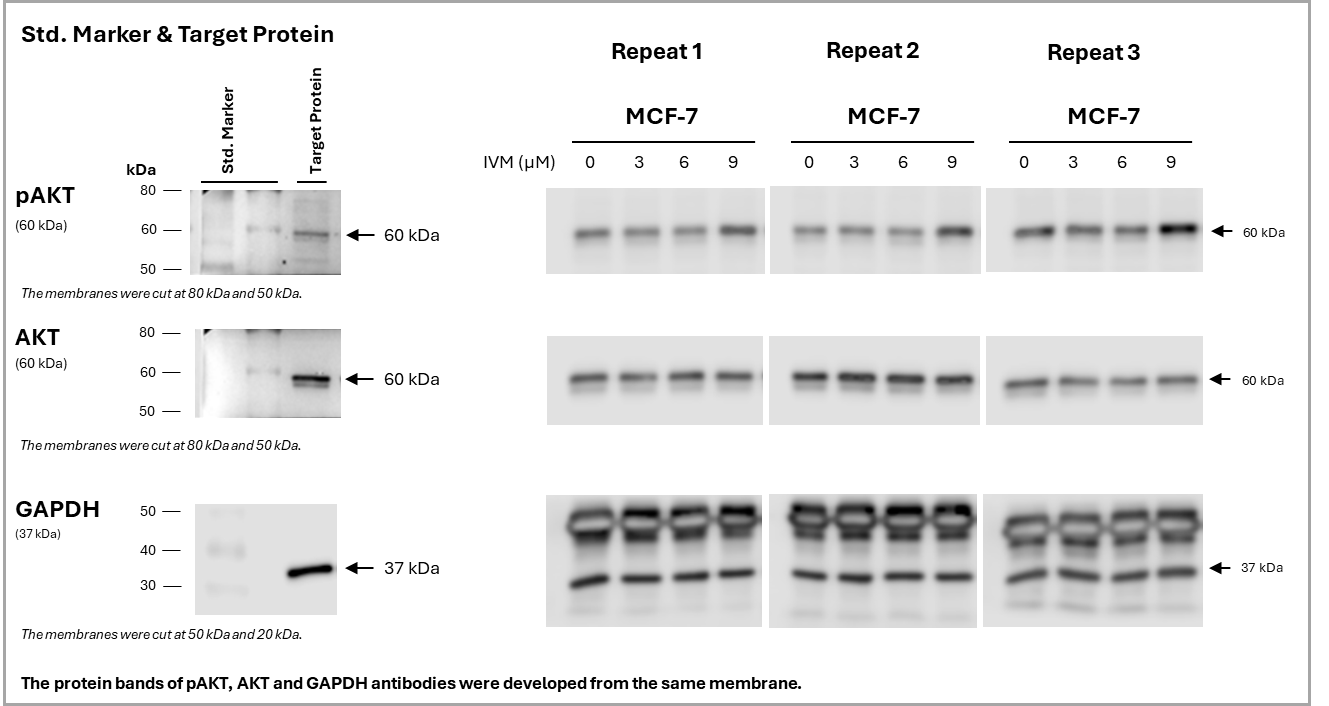


**Figure S8A, S8C, S8I**: MCF-7/LCC2


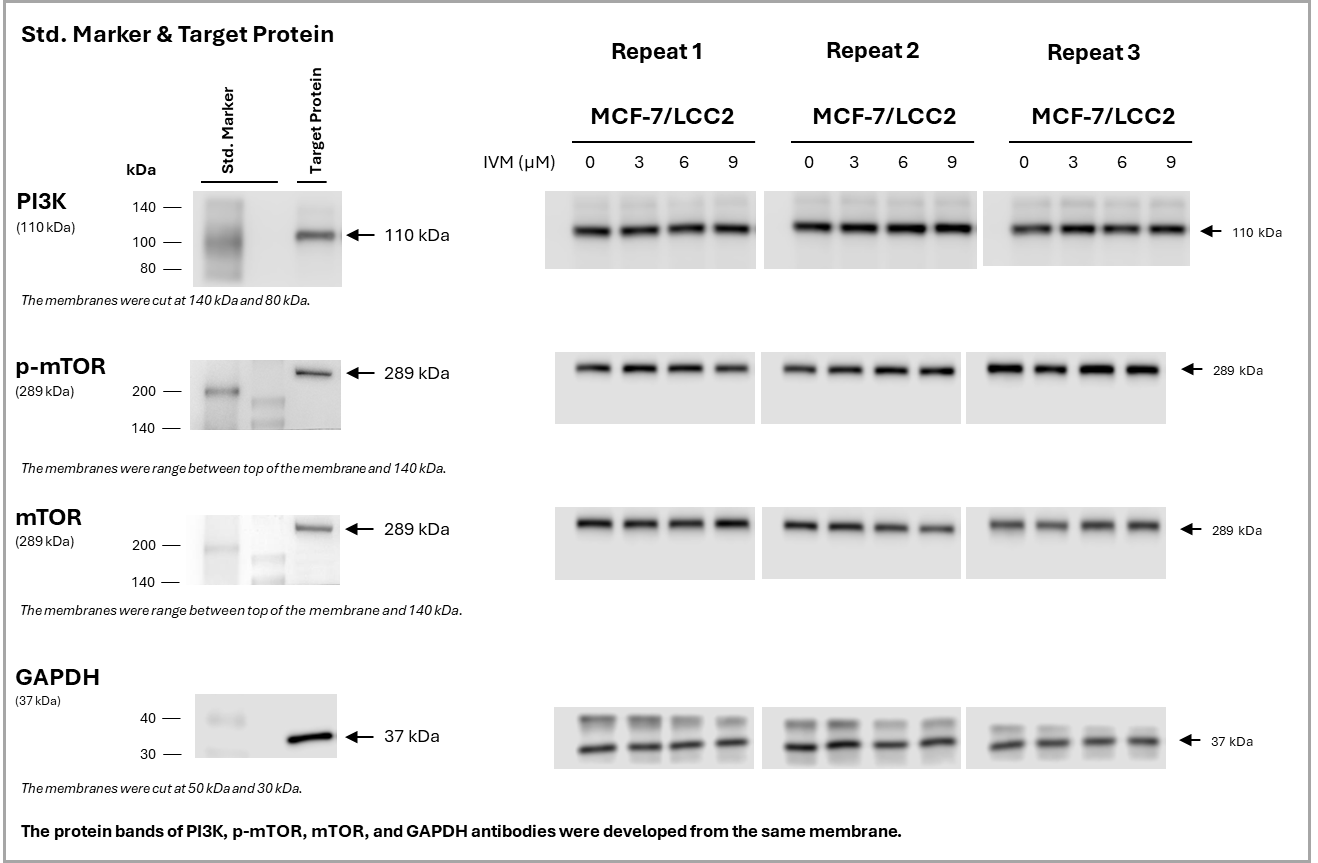


Note: Repeat 1, Repeat 2, and Repeat 3 used the same membrane as shown in the supplementary material for Figure 4A, 4C: MCF-7/LCC2

**Figure S8A, S8F**: MCF-7/LCC2


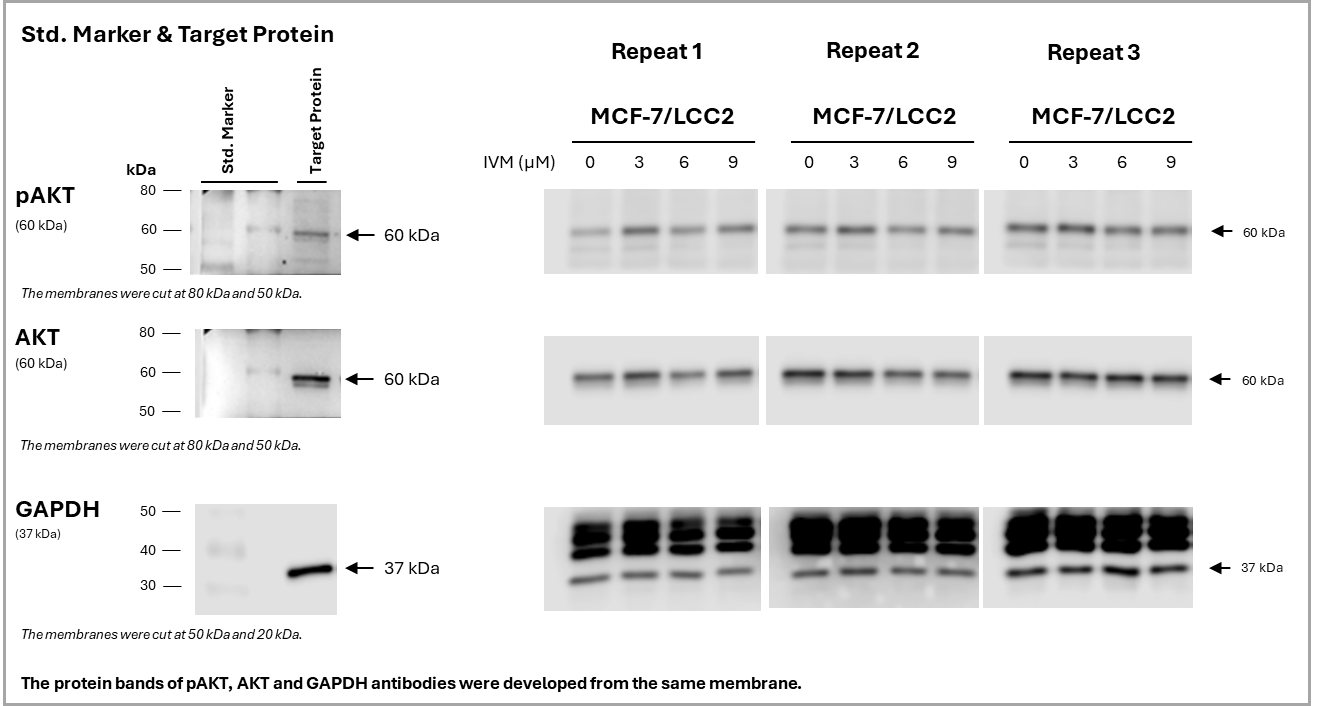


Note: Repeat 1, Repeat 2, and Repeat 3 used the same membrane as shown in the supplementary material for Figure 4A, 4F: MCF-7/LCC2

**Figure S8A, S8D, S8J**: MCF-7/LCC9


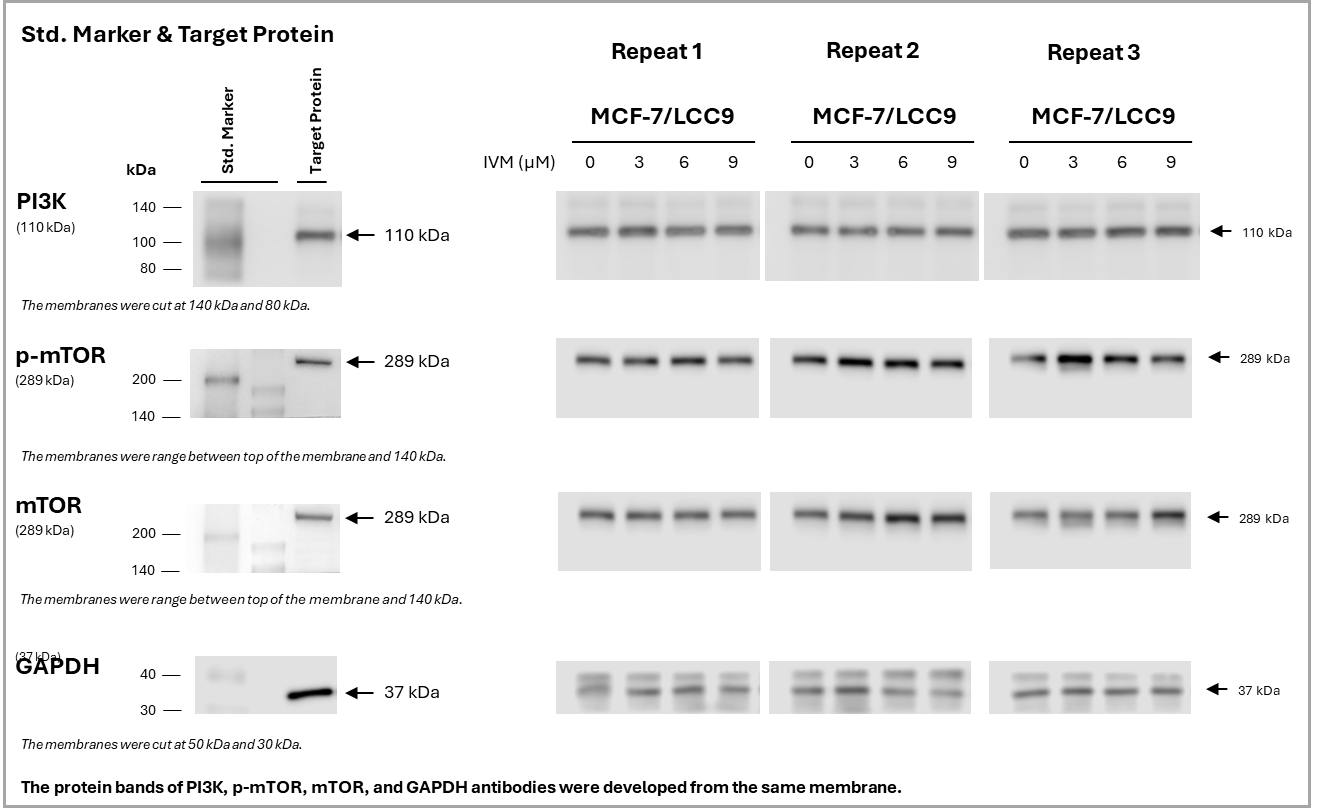


Note: Repeat 1, Repeat 2, and Repeat 3 used the same membrane as shown in the supplementary material for Figure 4A, 4D: MCF-7/LCC9

**Figure S8A, S8G**: MCF-7/LCC9


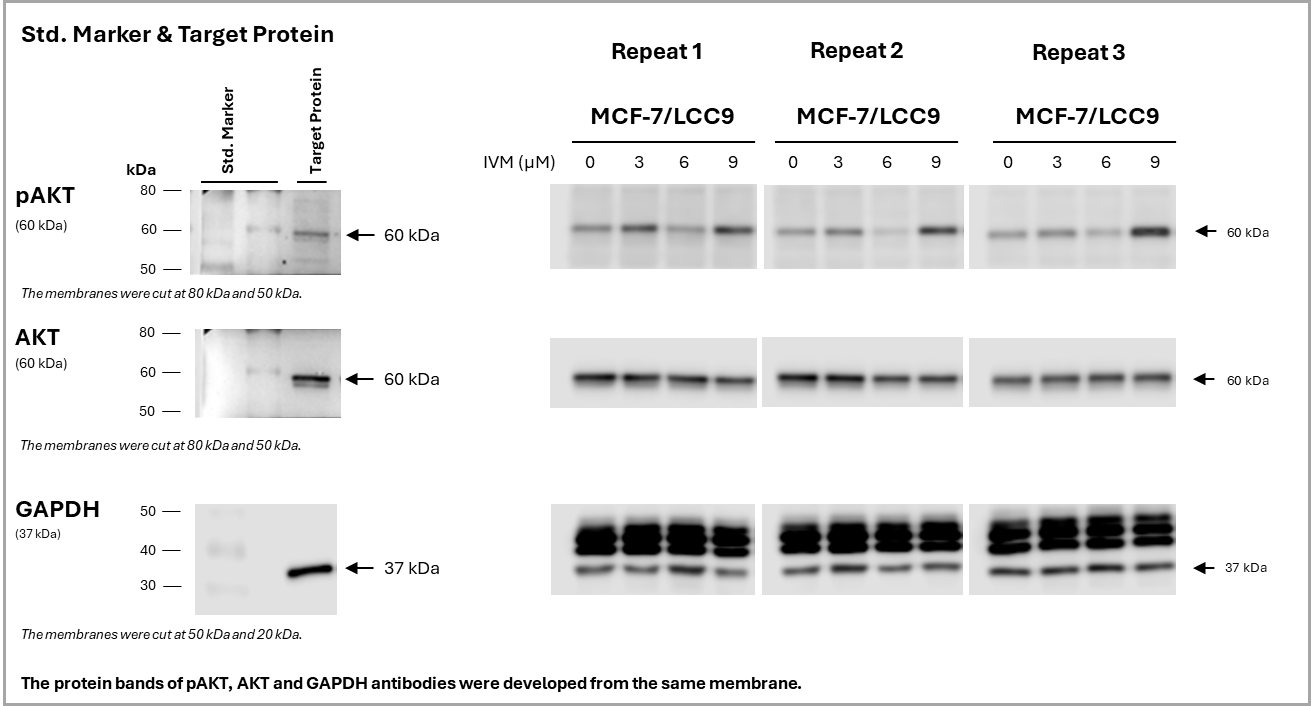


Note: Repeat 1, Repeat 2, and Repeat 3 used the same membrane as shown in the supplementary material for Figure 4A, 4G: MCF-7/LCC9
