# Supplementary material for: Ivermectin inhibits ER, HER2, and TGF-β pathways in ER-positive and endocrine-resistant breast cancer cells
Source: PLoS One. 2026 Apr 30;21(4):e0348260. doi: 10.1371/journal.pone.0348260 (PMC13132456; doi:10.1371/journal.pone.0348260)
Supplement: S2 File — (DOCX) [file pone.0348260.s014.docx]

**Supplementary Data and Statistical Analysis**

**Supplementary Table 1.** Raw data of %cell viability after IVM-treatment for 24 h

| **MCF-7** | **%Cell viability** | | |  | **LCC2** | **%Cell viability** | | |  | **LCC9** | **%Cell viability** | | |
| --- | --- | --- | --- | --- | --- | --- | --- | --- | --- | --- | --- | --- | --- |
| **Conc. (µM)** | **N1** | **N2** | **N3** |  | **Conc. (µM)** | **N1** | **N2** | **N3** |  | **Conc. (µM)** | **N1** | **N2** | **N3** |
| 0 | 100.00 | 100.00 | 100.00 |  | 0 | 100.00 | 100.00 | 100.00 |  | 0 | 100.00 | 100.00 | 100.00 |
| 3.125 | 87.67 | 94.46 | 96.52 |  | 1.5625 | 89.93 | 91.32 | 88.19 |  | 3.125 | 83.82 | 85.89 | 87.14 |
| 6.25 | 89.07 | 83.63 | 83.64 |  | 3.125 | 75.28 | 78.54 | 78.97 |  | 6.25 | 60.67 | 63.15 | 63.62 |
| 12.5 | 42.69 | 43.30 | 38.50 |  | 12.5 | 32.22 | 42.10 | 42.11 |  | 12.5 | 42.84 | 45.18 | 41.22 |
| 25 | 6.70 | 8.19 | 7.37 |  | 25 | 12.51 | 17.62 | 20.38 |  | 25 | 6.72 | 6.76 | 6.04 |
| 50 | 1.83 | 2.20 | 1.82 |  | 50 | 2.68 | 4.22 | 4.49 |  | 50 | 1.21 | 1.23 | 1.29 |

| **T-47D** | **%Cell viability** | | |  | **T-47D Tam1** | **%Cell viability** | | |  | **T47D-182R1** | **%Cell viability** | | |
| --- | --- | --- | --- | --- | --- | --- | --- | --- | --- | --- | --- | --- | --- |
| **Conc. (µM)** | **N1** | **N2** | **N3** |  | **Conc. (µM)** | **N1** | **N2** | **N3** |  | **Conc. (µM)** | **N1** | **N2** | **N3** |
| 0 | 100.00 | 100.00 | 100.00 |  | 0 | 100.00 | 100.00 | 100.00 |  | 0 | 100.00 | 100.00 | 100.00 |
| 1.5625 | 110.56 | 126.55 | 103.56 |  | 1.5625 | 112.69 | 128.06 | 108.94 |  | 1.5625 | 116.47 | 107.14 | 111.79 |
| 3.125 | 114.43 | 116.30 | 102.04 |  | 3.125 | 114.37 | 133.44 | 114.80 |  | 3.125 | 126.12 | 117.91 | 122.18 |
| 6.25 | 101.86 | 109.86 | 97.84 |  | 6.25 | 87.59 | 118.82 | 99.93 |  | 6.25 | 107.32 | 105.18 | 123.27 |
| 12.5 | 14.47 | 20.50 | 17.00 |  | 12.5 | 21.34 | 28.92 | 25.72 |  | 12.5 | 15.14 | 24.33 | 11.38 |
| 25 | 11.21 | 17.08 | 15.21 |  | 25 | 16.95 | 23.66 | 17.19 |  | 25 | 14.83 | 23.08 | 10.52 |
| 50 | 10.89 | 22.20 | 14.70 |  | 50 | 17.29 | 23.44 | 16.64 |  | 50 | 15.39 | 25.26 | 10.79 |

**Supplementary Table 2.** Raw data of protein expression in relative to non-treatment (Fold Change-Mean ± SEM) after IVM-treatment at 3, 6, and 9 µM, respectively, for 24 h

| **Cell line** | **Target protein** | **Protein expression in relative to non-treatment (Fold Change-Mean ± SEM)** | | | |  |
| --- | --- | --- | --- | --- | --- | --- |
|  |  | **IVM 0 µM** | **IVM 3 µM** | **IVM 6 µM** | **IVM 9 µM** |  |
| MCF-7 | ERα | 1.00 ± 0.00 | 0.96 ± 0.06 | 0.77 ± 0.1 | 0.52 ± 0.06 |  |
| MCF-7 | HER2 | 1.00 ± 0.00 | 1.10 ± 0.14 | 0.89 ± 0.07 | 0.66 ± 0.03 |  |
| MCF-7 | Cyclin D1 | 1.00 ± 0.00 | 0.96 ± 0.06 | 0.93 ± 0.11 | 0.58 ± 0.05 |  |
| MCF-7 | pHER2 | 1.00 ± 0.00 | 1.20 ± 0.07 | 1.10 ± 0.01 | 1.10 ± 0.10 |  |
| MCF-7/LCC2 | ERα | 1.00 ± 0.00 | 0.94 ± 0.02 | 0.68 ± 0.05 | 0.41 ± 0.01 |  |
| MCF-7/LCC2 | HER2 | 1.00 ± 0.00 | 1.20 ± 0.12 | 0.85 ± 0.08 | 0.58 ± 0.02 |  |
| MCF-7/LCC2 | Cyclin D1 | 1.00 ± 0.00 | 1.30 ± 0.09 | 1.20 ± 0.06 | 1.20 ± 0.14 |  |
| MCF-7/LCC2 | pHER2 | 1.00 ± 0.00 | 1.10 ± 0.19 | 0.90 ± 0.14 | 0.52 ± 0.08 |  |
| MCF-7/LCC9 | ERα | 1.00 ± 0.00 | 1.10 ± 0.05 | 1.10 ± 0.13 | 0.71 ± 0.06 |  |
| MCF-7/LCC9 | HER2 | 1.00 ± 0.00 | 1.30 ± 0.19 | 1.50 ± 0.04 | 1.10 ± 0.10 |  |
| MCF-7/LCC9 | Cyclin D1 | 1.00 ± 0.00 | 0.84 ± 0.23 | 1.1 ± 0.37 | 1.1 ± 0.41 |  |

| **Cell line** | **Target protein** | **Protein expression in relative to non-treatment (Fold Change-Mean ± SEM)** | | | |
| --- | --- | --- | --- | --- | --- |
|  |  | **IVM 0 µM** | **IVM 3 µM** | **IVM 6 µM** | **IVM 9 µM** |
| MCF-7 | pSMAD2/SMAD2 | 1.00 ± 0.00 | 0.75 ± 0.06 | 0.67 ± 0.05 | 0.65 ± 0.04 |
| MCF-7 | SMAD2 | 1.00 ± 0.00 | 1.20 ± 0.13 | 1.10 ± 0.07 | 1.20 ± 0.15 |
| MCF-7 | pERK/ERK | 1.00 ± 0.00 | 0.50 ± 0.05 | 0.36 ± 0.01 | 0.33 ± 0.04 |
| MCF-7 | ERK | 1.00 ± 0.00 | 1.30 ± 0.17 | 1.10 ± 0.09 | 1.20 ± 0.22 |
| MCF-7/LCC2 | pSMAD2/SMAD2 | 1.00 ± 0.00 | 0.63 ± 0.02 | 0.42 ± 0.03 | 0.27 ± 0.02 |
| MCF-7/LCC2 | SMAD2 | 1.00 ± 0.00 | 1.20 ± 0.18 | 1.20 ± 0.13 | 1.10 ± 0.13 |
| MCF-7/LCC2 | pERK/ERK | 1.00 ± 0.00 | 0.91 ± 0.06 | 0.51 ± 0.06 | 0.06 ± 0.01 |
| MCF-7/LCC2 | ERK | 1.00 ± 0.00 | 1.20 ± 0.13 | 1.20 ± 0.18 | 1.10 ± 0.14 |
| MCF-7/LCC9 | pSMAD2/SMAD2 | 1.00 ± 0.00 | 0.77 ± 0.03 | 0.52 ± 0.01 | 0.41 ± 0.03 |
| MCF-7/LCC9 | SMAD2 | 1.00 ± 0.00 | 1.00 ± 0.07 | 1.20 ± 0.02 | 1.30 ± 0.14 |
| MCF-7/LCC9 | pERK/ERK | 1.00 ± 0.00 | 1.20 ± 0.19 | 0.86 ± 0.07 | 0.31 ± 0.01 |
| MCF-7/LCC9 | ERK | 1.00 ± 0.00 | 1.10 ± 0.24 | 1.00 ± 0.13 | 0.96 ± 0.12 |

| **Cell line** | **Target protein** | **Protein expression in relative to non-treatment (Fold Change-Mean ± SEM)** | | | |
| --- | --- | --- | --- | --- | --- |
|  |  | **IVM 0 µM** | **IVM 3 µM** | **IVM 6 µM** | **IVM 9 µM** |
| MCF-7 | pSMAD3/SMAD3 | 1.00 ± 0.00 | 0.8 ± 0.03 | 0.69 ± 0.10 | 0.44 ± 0.06 |
| MCF-7 | SMAD3 | 1.00 ± 0.00 | 0.97 ± 0.03 | 1.36 ± 0.04 | 1.36 ± 0.11 |
| MCF-7 | SMAD4 | 1.00 ± 0.00 | 1.20 ± 0.39 | 1.50 ± 0.48 | 1.20 ± 0.33 |
| MCF-7 | pPAK-1/PAK-1 | 1.00 ± 0.00 | 1.20 ± 0.16 | 1.10 ± 0.07 | 0.97 ± 0.08 |
| MCF-7 | PAK-1 | 1.00 ± 0.00 | 1.10 ± 0.11 | 0.95 ± 0.07 | 0.97 ± 0.06 |
| MCF-7/LCC2 | pSMAD3/SMAD3 | 1.00 ± 0.00 | 0.79 ± 0.07 | 0.52 ± 0.19 | 0.44 ± 0.08 |
| MCF-7/LCC2 | SMAD3 | 1.00 ± 0.00 | 1.50 ± 0.34 | 2.30 ± 0.50 | 2.50 ± 1.20 |
| MCF-7/LCC2 | SMAD4 | 1.00 ± 0.00 | 1.20 ± 0.12 | 1.30 ± 0.13 | 1.60 ± 0.42 |
| MCF-7/LCC2 | pPAK-1/PAK-1 | 1.00 ± 0.00 | 0.99 ± 0.05 | 1.00 ± 0.06 | 0.72 ± 0.04 |
| MCF-7/LCC2 | PAK-1 | 1.00 ± 0.00 | 1.20 ± 0.07 | 0.95 ± 0.08 | 0.70 ± 0.06 |
| MCF-7/LCC9 | pSMAD3/SMAD3 | 1.00 ± 0.00 | 1.50 ± 0.91 | 0.66 ± 0.08 | 0.72 ± 0.17 |
| MCF-7/LCC9 | SMAD3 | 1.00 ± 0.00 | 1.80 ± 0.16 | 2.10 ± 0.43 | 1.60 ± 0.25 |
| MCF-7/LCC9 | SMAD4 | 1.00 ± 0.00 | 0.96 ± 0.13 | 0.99 ± 0.20 | 0.82 ± 0.11 |
| MCF-7/LCC9 | pPAK-1/PAK-1 | 1.00 ± 0.00 | 1.40 ± 0.23 | 1.30 ± 0.29 | 1.40 ± 0.28 |
| MCF-7/LCC9 | PAK-1 | 1.00 ± 0.00 | 0.87 ± 0.04 | 1.20 ± 0.21 | 1.10 ± 0.40 |

| **Cell line** | **Target protein** | **Protein expression in relative to non-treatment (Fold Change-Mean ± SEM)** | | | |
| --- | --- | --- | --- | --- | --- |
|  |  | **IVM 0 µM** | **IVM 3 µM** | **IVM 6 µM** | **IVM 9 µM** |
| MCF-7 | PI3K | 1.00 ± 0.00 | 1.10 ± 0.17 | 0.99 ± 0.05 | 1.10 ± 0.18 |
| MCF-7 | pAKT/AKT | 1.00 ± 0.00 | 1.10 ± 0.14 | 1.00 ± 0.10 | 1.50 ± 0.08 |
| MCF-7 | AKT | 1.00 ± 0.00 | 0.87 ± 0.15 | 0.95 ± 0.12 | 0.97 ± 0.11 |
| MCF-7 | p-mTOR/mTOR | 1.00 ± 0.00 | 1.40 ± 0.02 | 1.10 ± 0.05 | 1.20 ± 0.23 |
| MCF-7 | mTOR | 1.00 ± 0.00 | 1.10 ± 0.23 | 1.10 ± 0.15 | 1.40 ± 0.35 |
| MCF-7/LCC2 | PI3K | 1.00 ± 0.00 | 1.10 ± 0.21 | 1.10 ± 0.09 | 0.98 ± 0.05 |
| MCF-7/LCC2 | pAKT/AKT | 1.00 ± 0.00 | 1.50 ± 0.19 | 1.20 ± 0.28 | 1.30 ± 0.22 |
| MCF-7/LCC2 | AKT | 1.00 ± 0.00 | 1.00 ± 0.17 | 0.95 ± 0.04 | 1.00 ± 0.14 |
| MCF-7/LCC2 | p-mTOR/mTOR | 1.00 ± 0.00 | 1.20 ± 0.09 | 1.20 ± 0.12 | 1.20 ± 0.25 |
| MCF-7/LCC2 | mTOR | 1.00 ± 0.00 | 0.97 ± 0.15 | 1.10 ± 0.10 | 0.96 ± 0.14 |
| MCF-7/LCC9 | PI3K | 1.00 ± 0.00 | 0.97 ± 0.08 | 1.10 ± 0.05 | 1.20 ± 0.12 |
| MCF-7/LCC9 | pAKT/AKT | 1.00 ± 0.00 | 1.40 ± 0.16 | 0.73 ± 0.20 | 2.80 ± 0.04 |
| MCF-7/LCC9 | AKT | 1.00 ± 0.00 | 1.10 ± 0.18 | 0.90 ± 0.09 | 0.98 ± 0.09 |
| MCF-7/LCC9 | p-mTOR/mTOR | 1.00 ± 0.00 | 1.30 ± 0.22 | 1.10 ± 0.14 | 0.90 ± 0.11 |
| MCF-7/LCC9 | mTOR | 1.00 ± 0.00 | 1.10 ± 0.04 | 1.20 ± 0.13 | 1.40 ± 0.16 |

**Supplementary Table 3.** Raw data of %cell viability in relative to non-treatment after IVM-treatment with/without estradiol (E_2_) induced for 5 days

| **MCF-7** | **%Cell viability** | | | | | |
| --- | --- | --- | --- | --- | --- | --- |
|  | **-E_2_** | | | **+E_2_** | | |
| **Conc.** | **N1** | **N2** | **N3** | **N1** | **N2** | **N3** |
| IVM 0 µM | 100.00 | 100.00 | 100.00 | 244.51 | 217.88 | 243.36 |
| IVM 1 µM | 118.70 | 106.97 | 112.21 | 227.10 | 200.55 | 238.86 |
| IVM 3 µM | 105.83 | 96.79 | 101.74 | 188.62 | 158.26 | 179.13 |
| IVM 5 µM | 84.21 | 76.51 | 70.60 | 106.50 | 103.95 | 116.64 |
| 4-OHT 5 µM | 49.53 | 51.42 | 50.47 | 45.46 | 45.44 | 50.40 |
| 4-OHT 10 µM | 11.92 | 12.89 | 11.81 | 12.13 | 12.08 | 11.95 |

| **T-47D** | **%Cell viability** | | | | | |
| --- | --- | --- | --- | --- | --- | --- |
|  | **-E_2_** | | | **+E_2_** | | |
| **Conc.** | **N1** | **N2** | **N3** | **N1** | **N2** | **N3** |
| IVM 0 µM | 100.00 | 100.00 | 100.00 | 151.62 | 170.60 | 178.31 |
| IVM 1 µM | 93.29 | 99.46 | 98.23 | 139.79 | 166.23 | 163.54 |
| IVM 3 µM | 81.07 | 83.17 | 88.35 | 125.88 | 142.63 | 143.71 |
| IVM 5 µM | 57.07 | 57.25 | 68.27 | 60.21 | 66.71 | 75.44 |
| 4-OHT 5 µM | 42.83 | 41.32 | 44.14 | 29.89 | 30.30 | 30.89 |
| 4-OHT 10 µM | 9.95 | 11.50 | 17.22 | 9.32 | 11.62 | 16.88 |

| **MCF-7/LCC2** | **%Cell viability** | | | | | |
| --- | --- | --- | --- | --- | --- | --- |
|  | **-E_2_** | | | **+E_2_** | | |
| **Conc.** | **N1** | **N2** | **N3** | **N1** | **N2** | **N3** |
| IVM 0 µM | 100.00 | 100.00 | 100.00 | 86.27 | 92.30 | 88.12 |
| IVM 1 µM | 88.03 | 94.13 | 97.08 | 81.27 | 81.87 | 81.64 |
| IVM 3 µM | 49.71 | 63.15 | 63.40 | 38.07 | 57.40 | 65.49 |
| IVM 5 µM | 27.25 | 32.60 | 33.07 | 15.86 | 19.19 | 22.54 |
| 4-OHT 5 µM | 67.17 | 66.47 | 66.41 | 66.02 | 69.87 | 68.97 |
| 4-OHT 10 µM | 4.59 | 9.32 | 7.83 | 6.84 | 6.55 | 4.70 |
|  |  |  |  |  |  |  |
| **MCF-7/LCC9** | **%Cell viability** | | | | | |
|  | **-E_2_** | | | **+E_2_** | | |
| **Conc.** | **N1** | **N2** | **N3** | **N1** | **N2** | **N3** |
| IVM 0 µM | 100.00 | 100.00 | 100.00 | 93.98 | 99.45 | 95.75 |
| IVM 1 µM | 79.31 | 84.80 | 80.14 | 89.20 | 89.95 | 84.65 |
| IVM 3 µM | 70.94 | 75.29 | 70.23 | 67.77 | 65.32 | 64.87 |
| IVM 5 µM | 30.61 | 27.67 | 26.66 | 29.98 | 33.28 | 34.76 |
| 4-OHT 5 µM | 51.23 | 52.18 | 52.59 | 51.20 | 55.77 | 53.33 |
| 4-OHT 10 µM | 3.24 | 3.51 | 3.22 | 2.89 | 2.88 | 2.74 |

**Supplementary Table 4.** Raw data of protein expression in relative to non-treatment (Fold Change-Mean ± SEM) in E_2_ induced/non-induced with IVM-treatment at 8, 10, and 13 µM, respectively, for 24 h

| **MCF-7** | **ERα (Fold Change)** | |  | **MCF-7** | **HER2 (Fold Change)** | |
| --- | --- | --- | --- | --- | --- | --- |
| **Conc.** | **-E_2_** | **+E_2_** |  | **Conc.** | **-E_2_** | **+E_2_** |
| IVM 0 µM | 1.00 ± 0.00 | 0.82 ± 0.04 |  | IVM 0 µM | 1.00 ± 0.00 | 0.79 ± 0.04 |
| IVM 8 µM | 0.45 ± 0.14 | 0.20 ± 0.05 |  | IVM 8 µM | 0.60 ± 0.11 | 0.40 ± 0.02 |
| IVM 10 µM | 0.45 ± 0.12 | 0.21 ± 0.1 |  | IVM 10 µM | 0.61 ± 0.03 | 0.41 ± 0.05 |
| IVM 13 µM | 0.52 ± 0.23 | 0.3 ± 0.16 |  | IVM 13 µM | 0.52 ± 0.23 | 0.10 ± 0.07 |

**Supplementary Table 5.** Raw data of qPCR quantification of mRNA expression in relative to non-treatment (Fold Change-Mean ± SEM) in E_2_ induced/non-induced with IVM-treatment at 8, 10, and 13 µM, respectively, for 24 h

| **MCF-7** | ***ERα* (Fold Change)** | |
| --- | --- | --- |
| **Conc.** | **-E_2_** | **+E_2_** |
| IVM 0 µM | 1.00 ± 0.00 | 1.05 ± 0.43 |
| IVM 8 µM | 0.38 ± 0.14 | 2.11 ± 0.44 |
| IVM 10 µM | 0.22 ± 0.06 | 1.76 ± 0.83 |
| IVM 13 µM | 0.43 ± 0.21 | 1.54 ± 0.54 |

| **MCF-7** | ***pS2* (Fold Change)** |
| --- | --- |
| **Conc.** |  |
| IVM 0 µM | 1.00 ± 0.00 |
| IVM 8 µM | 0.38 ± 0.14 |
| IVM 10 µM | 0.22 ± 0.06 |
| IVM 13 µM | 0.43 ± 0.21 |

**Supplementary Table 6.** Raw data of %cell viability in relative to non-treatment after combination treatment between IVM and 4-OHT for 24 h

| **Cell line** | **Conc. (µM)** | **%Cell viability** | | | |
| --- | --- | --- | --- | --- | --- |
|  |  | **N1** | **N2** | **N3** | **Mean ± SEM** |
| MCF-7 | IVM 0 µM | 100.00 | 100.00 | 100.00 | 100.00 ± 0.00 |
|  | 4-OHT 5 µM | 90.53 | 91.93 | 95.80 | 92.75 ± 1.58 |
|  | IVM 8 µM | 69.98 | 65.89 | 75.40 | 70.42 ± 2.75 |
|  | 4-OHT 5 µM+IVM 8 µM | 52.66 | 42.71 | 44.20 | 46.52 ± 3.10 |
| MCF-7/LCC2 | IVM 0 µM | 100.00 | 100.00 | 100.00 | 100.00 ± 0.00 |
|  | 4-OHT 9 µM | 81.79 | 91.33 | 89.88 | 87.67 ± 2.97 |
|  | IVM 5 µM | 83.44 | 83.67 | 78.57 | 81.89 ± 1.66 |
|  | 4-OHT 9 µM+IVM 5 µM | 50.66 | 58.67 | 56.25 | 55.19 ± 2.37 |
| MCF-7/LCC9 | IVM 0 µM | 100.00 | 100.00 | 100.00 | 100.00 ± 0.00 |
|  | 4-OHT 8 µM | 89.19 | 92.75 | 86.37 | 89.44 ± 1.85 |
|  | IVM 4 µM | 73.28 | 78.82 | 77.52 | 76.54 ± 1.67 |
|  | 4-OHT 8 µM+IVM 4 µM | 51.08 | 64.89 | 63.36 | 59.78 ± 4.37 |

**Supplementary Table 7.** Raw data of target protein expression in relative to non-treatment (Fold Change-Mean ± SEM) after combination treatment between IVM and 4-OHT for 24 h

| **Cell line** | **Target protein** | **Protein expression in relative to non-treatment (Fold Change-Mean ± SEM)** | | | |
| --- | --- | --- | --- | --- | --- |
|  |  | **IVM 0 µM** | **4-OHT 5 µM** | **IVM 8 µM** | **4-OHT 5 µM + IVM 8 µM** |
| MCF-7 | ERα | 1.00 ± 0.00 | 1.41 ± 0.17 | 0.76 ± 0.13 | 0.78 ± 0.16 |
| MCF-7 | HER2 | 1.00 ± 0.00 | 0.95 ± 0.1 | 0.90 ± 0.13 | 0.50 ± 0.04 |
|  |  |  |  |  |  |
| **Cell line** | **Target protein** | **Protein expression in relative to non-treatment (Fold Change-Mean ± SEM)** | | | |
|  |  | **IVM 0 µM** | **4-OHT 9 µM** | **IVM 5 µM** | **4-OHT 9 µM + IVM 5 µM** |
| MCF-7/LCC2 | ERα | 1.00 ± 0.00 | 1.51 ± 0.12 | 0.83 ± 0.06 | 0.65 ± 0.10 |
| MCF-7/LCC2 | HER2 | 1.00 ± 0.00 | 0.60 ± 0.06 | 0.79 ± 0.04 | 0.51 ± 0.09 |
|  |  |  |  |  |  |
| **Cell line** | **Target protein** | **Protein expression in relative to non-treatment (Fold Change-Mean ± SEM)** | | | |
|  |  | **IVM 0 µM** | **4-OHT 8 µM** | **IVM 4 µM** | **4-OHT 8 µM + IVM 4 µM** |
| MCF-7/LCC9 | ERα | 1.00 ± 0.00 | 1.83 ± 0.23 | 1.14 ± 0.08 | 1.24 ± 0.11 |
| MCF-7/LCC9 | HER2 | 1.00 ± 0.00 | 1.09 ± 0.12 | 0.92 ± 0.09 | 0.79 ± 0.12 |
|  |  |  |  |  |  |
| **Cell line** | **Target protein** | **Protein expression in relative to non-treatment (Fold Change-Mean ± SEM)** | | | |
|  |  | **IVM 0 µM** | **4-OHT 9 µM** | **IVM 8 µM** | **4-OHT 9 µM + IVM 8 µM** |
| T-47D | ERα | 1.00 ± 0.00 | 1.30 ± 0.15 | 0.60 ± 0.01 | 0.41 ± 0.04 |
| T-47D | HER2 | 1.00 ± 0.00 | 0.77 ± 0.03 | 0.78 ± 0.05 | 0.54 ± 0.04 |
| T-47D Tam1 | ERα | 1.00 ± 0.00 | 0.49 ± 0.38 | 0.26 ± 0.05 | 0.02 ± 0.02 |
| T-47D Tam1 | HER2 | 1.00 ± 0.00 | 0.67 ± 0.29 | 0.57 ± 0.06 | 0.31 ± 0.09 |
| T47D 182R1 | HER2 | 1.00 ± 0.00 | 0.59 ± 0.15 | 0.64 ± 0.02 | 0.35 ± 0.07 |

**Supplementary Table 8.** Raw data of %cell viability after IVM treatment of breast cancer cells in a time-dependent manner.

| **MCF-7** | **%Cell viability** | | | | | | | | |
| --- | --- | --- | --- | --- | --- | --- | --- | --- | --- |
| **Time** | **IVM 3.12 (µM)** | | | **IVM 6.25 (µM)** | | | **IVM 12.5 (µM)** | | |
|  | **N1** | **N2** | **N3** | **N1** | **N2** | **N3** | **N1** | **N2** | **N3** |
| 24h | 103.42 | 105.64 | 98.64 | 82.24 | 82.64 | 75.43 | 30.69 | 41.77 | 35.43 |
| 48h | 77.85 | 82.52 | 80.31 | 46.25 | 51.97 | 43.59 | 10.61 | 13.80 | 10.02 |
| 72h | 75.82 | 69.32 | 71.35 | 36.84 | 31.75 | 33.73 | 5.59 | 5.27 | 4.51 |
|  |  |  |  |  |  |  |  |  |  |
| **MCF-7/LCC2** | **%Cell viability** | | | | | | | | |
| **Time** | **IVM 3.12 (µM)** | | | **IVM 6.25 (µM)** | | | **IVM 12.5 (µM)** | | |
|  | **N1** | **N2** | **N3** | **N1** | **N2** | **N3** | **N1** | **N2** | **N3** |
| 24h | 89.10 | 90.42 | 87.55 | 69.47 | 70.17 | 62.96 | 35.58 | 41.63 | 37.17 |
| 48h | 80.35 | 84.88 | 69.47 | 53.93 | 52.10 | 52.54 | 11.73 | 13.32 | 10.96 |
| 72h | 67.77 | 81.79 | 72.43 | 46.78 | 43.32 | 42.73 | 4.10 | 3.51 | 4.85 |
|  |  |  |  |  |  |  |  |  |  |
| **MCF-7/LCC9** | **%Cell viability** | | | | | | | | |
| **Time** | **IVM 3.12 (µM)** | | | **IVM 6.25 (µM)** | | | **IVM 12.5 (µM)** | | |
|  | **N1** | **N2** | **N3** | **N1** | **N2** | **N3** | **N1** | **N2** | **N3** |
| 24h | 88.98 | 75.51 | 81.61 | 70.61 | 60.39 | 59.78 | 39.74 | 39.66 | 31.45 |
| 48h | 81.42 | 58.98 | 72.73 | 58.09 | 49.81 | 45.04 | 8.15 | 10.07 | 7.77 |
| 72h | 72.74 | 73.79 | 83.92 | 45.68 | 41.80 | 30.66 | 5.30 | 5.16 | 5.03 |

All raw data were obtained from from Rujimongkon et al (2025), PLoS One 20(6): e0326742

**Supplementary Table 9.** Raw data of %cell viability of combined treatment of IVM and 4-OHT in human normal fibroblast (CRL-1474) and breast cancer cell lines (MCF-7)

| **4-OHT (µM)** | **%Cell viability of CRL-1474 cell line** | | | | | | | | |
| --- | --- | --- | --- | --- | --- | --- | --- | --- | --- |
|  | **IVM 0 µM** | | | **IVM 3 µM** | | | **IVM 9 µM** | | |
| 0 | 100.00 | 100.00 | 100.00 | 102.70 | 105.05 | 115.08 | 108.82 | 107.01 | 121.66 |
| 2.5 | 101.76 | 98.35 | 101.70 | 102.18 | 104.43 | 112.13 | 98.44 | 98.35 | 114.85 |
| 5 | 106.43 | 99.28 | 105.44 | 104.56 | 100.72 | 111.90 | 99.59 | 96.60 | 107.26 |
| 7.5 | 107.57 | 104.85 | 109.98 | 105.91 | 101.75 | 110.32 | 93.88 | 94.43 | 101.59 |
| 10 | 77.70 | 77.73 | 84.01 | 74.59 | 77.73 | 78.46 | 55.29 | 56.80 | 57.14 |
|  |  |  |  |  |  |  |  |  |  |
| **4-OHT (µM)** | **%Cell viability of MCF-7 cell line** | | | | | | | | |
|  | **IVM 0 µM** | | | **IVM 3 µM** | | | **IVM 9 µM** | | |
| 0 | 100.00 | 100.00 | 100.00 | 80.44 | 101.88 | 97.77 | 64.41 | 76.72 | 68.55 |
| 2.5 | 97.35 | 105.47 | 98.66 | 87.06 | 95.31 | 95.25 | 65.88 | 69.69 | 64.54 |
| 5 | 96.32 | 104.38 | 90.95 | 86.47 | 94.22 | 83.53 | 56.62 | 60.63 | 53.86 |
| 7.5 | 77.35 | 82.34 | 75.37 | 66.03 | 70.47 | 65.13 | 21.76 | 21.72 | 22.11 |
| 10 | 5.44 | 5.47 | 5.04 | 2.65 | 2.81 | 2.37 | 1.76 | 2.19 | 1.93 |

**Supplementary Table 10.** Combination Index of 4-OHT and IVM combination treatment for 72 h in breast cancer cells. The data of %cell viability were calculated by Compusyn software using raw data from Rujimongkon et al. (2025), which were analyzed under the Creative Commons Attribution 4.0 International (CC BY) license (Data for figure S7).

**Supplementary Table 11.** Statistical Analysis

| **Figure** | **Assay** | **Experimental detail/ Target Protein** | **Cell lines** | **Group comparisons** | **Mean Diff.** | **95% CI of diff.** | **P value** |
| --- | --- | --- | --- | --- | --- | --- | --- |
| Figure 1B | Western blot | ERα | MCF-7 | IVM 0 µM vs. IVM 3 µM | 0.0433 | -0.2239 to 0.3106 | 0.9353 |
|  |  |  |  | IVM 0 µM vs. IVM 6 µM | 0.2333 | -0.03389 to 0.5006 | 0.086 |
|  |  |  |  | IVM 0 µM vs. IVM 9 µM | 0.4800 | 0.2128 to 0.7472 | 0.0022 |
| Figure 1C | Western blot | ERα | MCF-7/LCC2 | IVM 0 µM vs. IVM 3 µM | 0.0566 | -0.05337 to 0.1666 | 0.3712 |
|  |  |  |  | IVM 0 µM vs. IVM 6 µM | 0.3250 | 0.2150 to 0.4350 | <0.0001 |
|  |  |  |  | IVM 0 µM vs. IVM 9 µM | 0.5925 | 0.4825 to 0.7025 | <0.0001 |
| Figure 1D | Western blot | ERα | MCF-7/LCC9 | IVM 0 µM vs. IVM 3 µM | -0.0406 | -0.04064 to 0.2227 | 0.1273 |
|  |  |  |  | IVM 0 µM vs. IVM 6 µM | 0.1427 | -0.2277 to 0.5130 | 0.3451 |
|  |  |  |  | IVM 0 µM vs. IVM 9 µM | -0.2901 | -0.4433 to -0.1370 | 0.0063 |
| Figure 1E | Western blot | HER2 | MCF-7 | IVM 0 µM vs. IVM 3 µM | -0.0900 | -0.4127 to 0.2327 | 0.7662 |
|  |  |  |  | IVM 0 µM vs. IVM 6 µM | 0.1100 | -0.2127 to 0.4327 | 0.6552 |
|  |  |  |  | IVM 0 µM vs. IVM 9 µM | 0.3367 | 0.01399 to 0.6593 | 0.0416 |
| Figure 1F | Western blot | HER2 | MCF-7/LCC2 | IVM 0 µM vs. IVM 3 µM | -0.1824 | -0.4843 to 0.1194 | 0.2634 |
|  |  |  |  | IVM 0 µM vs. IVM 6 µM | 0.1516 | -0.1503 to 0.4534 | 0.3886 |
|  |  |  |  | IVM 0 µM vs. IVM 9 µM | 0.4214 | 0.1196 to 0.7233 | 0.0098 |
| Figure 1G | Western blot | HER2 | MCF-7/LCC9 | IVM 0 µM vs. IVM 3 µM | -0.2833 | -1.024 to 0.4575 | 0.6513 |
|  |  |  |  | IVM 0 µM vs. IVM 6 µM | -0.5033 | -1.244 to 0.2375 | 0.217 |
|  |  |  |  | IVM 0 µM vs. IVM 9 µM | -0.0633 | -0.8042 to 0.6775 | 0.997 |

| **Figure** | **Assay** | **Experimental detail/ Target Protein** | **Cell lines** | **Group comparisons** | **Mean Diff.** | **95% CI of diff.** | **P value** |
| --- | --- | --- | --- | --- | --- | --- | --- |
| Figure 1H | Western blot | pHER2 | MCF-7 | IVM 0 µM vs. IVM 3 µM | -0.2000 | -0.4461 to 0.04614 | 0.1114 |
|  |  |  |  | IVM 0 µM vs. IVM 6 µM | -0.1400 | -0.3861 to 0.1061 | 0.3027 |
|  |  |  |  | IVM 0 µM vs. IVM 9 µM | -0.1167 | -0.3628 to 0.1295 | 0.4298 |
| Figure 1I | Western blot | pHER2 | MCF-7/LCC2 | IVM 0 µM vs. IVM 3 µM | -0.1000 | -0.6102 to 0.4102 | 0.8953 |
|  |  |  |  | IVM 0 µM vs. IVM 6 µM | 0.1033 | -0.4069 to 0.6135 | 0.8866 |
|  |  |  |  | IVM 0 µM vs. IVM 9 µM | 0.4800 | -0.03020 to 0.9902 | 0.0644 |
| Figure 1J | Western blot | Cyclin D1 | MCF-7 | IVM 0 µM vs. IVM 3 µM | 0.0433 | -0.2331 to 0.3198 | 0.9408 |
|  |  |  |  | IVM 0 µM vs. IVM 6 µM | 0.0733 | -0.2031 to 0.3498 | 0.7896 |
|  |  |  |  | IVM 0 µM vs. IVM 9 µM | 0.4233 | 0.1469 to 0.6998 | 0.0058 |
| Figure 1K | Western blot | Cyclin D1 | MCF-7/LCC2 | IVM 0 µM vs. IVM 3 µM | -0.2700 | -0.6243 to 0.08432 | 0.138 |
|  |  |  |  | IVM 0 µM vs. IVM 6 µM | -0.1900 | -0.5443 to 0.1643 | 0.3427 |
|  |  |  |  | IVM 0 µM vs. IVM 9 µM | -0.2267 | -0.5810 to 0.1277 | 0.2287 |
| Figure 1L | Western blot | Cyclin D1 | MCF-7/LCC9 | IVM 0 µM vs. IVM 3 µM | 0.1633 | -1.064 to 1.391 | 0.9619 |
|  |  |  |  | IVM 0 µM vs. IVM 6 µM | -0.1367 | -1.364 to 1.091 | 0.9767 |
|  |  |  |  | IVM 0 µM vs. IVM 9 µM | -0.0900 | -1.318 to 1.138 | 0.993 |

| **Figure** | **Assay** | **Experimental detail** | **Cell lines** | **Group comparisons** | **Mean Diff.** | **95% CI of diff.** | **P value** |
| --- | --- | --- | --- | --- | --- | --- | --- |
| Figure 2A | MTT | E_2_ induce cell proliferation | MCF-7 | -E2 |  |  |  |
|  |  |  |  | IVM 0 µM vs. IVM 1 µM | -12.6300 | -32.81 to 7.555 | 0.3346 |
|  |  |  |  | IVM 0 µM vs. IVM 3 µM | -1.4530 | -21.63 to 18.73 | 0.9997 |
|  |  |  |  | IVM 0 µM vs. IVM 5 µM | 22.8900 | 2.712 to 43.07 | 0.0224 |
|  |  |  |  | IVM 0 µM vs. 4-OHT 5 µM | 49.5300 | 29.35 to 69.71 | <0.0001 |
|  |  |  |  | IVM 0 µM vs. 4-OHT 10 µM | 87.7900 | 67.61 to 108.0 | <0.0001 |
|  |  |  |  |  |  |  |  |
|  |  |  |  | +E2 |  |  |  |
|  |  |  |  | IVM 0 µM vs. IVM 1 µM | 13.0800 | -7.101 to 33.26 | 0.3041 |
|  |  |  |  | IVM 0 µM vs. IVM 3 µM | 59.9100 | 39.73 to 80.09 | <0.0001 |
|  |  |  |  | IVM 0 µM vs. IVM 5 µM | 126.2000 | 106.0 to 146.4 | <0.0001 |
|  |  |  |  | IVM 0 µM vs. 4-OHT 5 µM | 188.2000 | 168.0 to 208.3 | <0.0001 |
|  |  |  |  | IVM 0 µM vs. 4-OHT 10 µM | 223.2000 | 203.0 to 243.4 | <0.0001 |
|  |  |  |  |  |  |  |  |
|  |  |  |  | -E2 vs. +E2 |  |  |  |
|  |  |  |  | IVM 0 µM | -135.3000 | -156.7 to -113.8 | <0.0001 |
|  |  |  |  | IVM 1 µM | -109.5000 | -131.0 to -88.08 | <0.0001 |
|  |  |  |  | IVM 3 µM | -73.8800 | -95.34 to -52.42 | <0.0001 |
|  |  |  |  | IVM 5 µM | -31.9200 | -53.38 to -10.46 | 0.0016 |
|  |  |  |  | 4-OHT 5 µM | 3.3730 | -18.09 to 24.83 | 0.9984 |
|  |  |  |  | 4-OHT 10 µM | 0.1533 | -21.31 to 21.61 | >0.9999 |

| **Figure** | **Assay** | **Experimental detail** | **Cell lines** | **Group comparisons** | **Mean Diff.** | **95% CI of diff.** | **P value** |
| --- | --- | --- | --- | --- | --- | --- | --- |
| Figure 2B | MTT | E_2_ induce cell proliferation | T-47D | -E2 |  |  |  |
|  |  |  |  | IVM 0 µM vs. IVM 1 µM | 3.0070 | -13.27 to 19.29 | 0.9821 |
|  |  |  |  | IVM 0 µM vs. IVM 3 µM | 15.8000 | -0.4753 to 32.08 | 0.0592 |
|  |  |  |  | IVM 0 µM vs. IVM 5 µM | 39.1400 | 22.86 to 55.42 | <0.0001 |
|  |  |  |  | IVM 0 µM vs. 4-OHT 5 µM | 57.2400 | 40.96 to 73.52 | <0.0001 |
|  |  |  |  | IVM 0 µM vs. 4-OHT 10 µM | 87.1100 | 70.83 to 103.4 | <0.0001 |
|  |  |  |  |  |  |  |  |
|  |  |  |  | +E2 |  |  |  |
|  |  |  |  | IVM 0 µM vs. IVM 1 µM | 10.3200 | -5.955 to 26.60 | 0.3228 |
|  |  |  |  | IVM 0 µM vs. IVM 3 µM | 29.4400 | 13.16 to 45.72 | 0.0003 |
|  |  |  |  | IVM 0 µM vs. IVM 5 µM | 99.3900 | 83.11 to 115.7 | <0.0001 |
|  |  |  |  | IVM 0 µM vs. 4-OHT 5 µM | 136.5000 | 120.2 to 152.8 | <0.0001 |
|  |  |  |  | IVM 0 µM vs. 4-OHT 10 µM | 154.2000 | 138.0 to 170.5 | <0.0001 |
|  |  |  |  |  |  |  |  |
|  |  |  |  | -E2 vs. +E2 |  |  |  |
|  |  |  |  | IVM 0 µM | -66.8400 | -84.15 to -49.53 | <0.0001 |
|  |  |  |  | IVM 1 µM | -59.5300 | -76.84 to -42.22 | <0.0001 |
|  |  |  |  | IVM 3 µM | -53.2100 | -70.52 to -35.90 | <0.0001 |
|  |  |  |  | IVM 5 µM | -6.5900 | -23.90 to 10.72 | 0.8675 |
|  |  |  |  | 4-OHT 5 µM | 12.4000 | -4.906 to 29.71 | 0.2698 |
|  |  |  |  | 4-OHT 10 µM | 0.2833 | -17.03 to 17.59 | >0.9999 |

| **Figure** | **Assay** | **Experimental detail/ Target Protein** | **Cell lines** | **Group comparisons** | **Mean Diff.** | **95% CI of diff.** | **P value** |
| --- | --- | --- | --- | --- | --- | --- | --- |
| Figure 2D | Western blot | E_2_ induce cell proliferation/ ERα | MCF-7 | -E2 |  |  |  |
|  |  |  |  | IVM 0 µM vs. IVM 8 µM | 0.5473 | 0.07525 to 1.019 | 0.021 |
|  |  |  |  | IVM 0 µM vs. IVM 10 µM | 0.5503 | 0.07825 to 1.022 | 0.0202 |
|  |  |  |  | IVM 0 µM vs. IVM 13 µM | 0.4847 | 0.01259 to 0.9567 | 0.0433 |
|  |  |  |  |  |  |  |  |
|  |  |  |  | +E2 |  |  |  |
|  |  |  |  | IVM 0 µM vs. IVM 8 µM | 0.6223 | 0.1503 to 1.094 | 0.0086 |
|  |  |  |  | IVM 0 µM vs. IVM 10 µM | 0.6107 | 0.1386 to 1.083 | 0.0099 |
|  |  |  |  | IVM 0 µM vs. IVM 13 µM | 0.5253 | 0.05325 to 0.9974 | 0.0271 |
|  |  |  |  |  |  |  |  |
|  |  |  |  | -E2 vs. +E2 |  |  |  |
|  |  |  |  | IVM 0 µM | 0.1790 | -0.3177 to 0.6757 | 0.7953 |
|  |  |  |  | IVM 8 µM | 0.2540 | -0.2427 to 0.7507 | 0.5275 |
|  |  |  |  | IVM 10 µM | 0.2393 | -0.2574 to 0.7361 | 0.5811 |
|  |  |  |  | IVM 13 µM | 0.2197 | -0.2771 to 0.7164 | 0.6537 |

| **Figure** | **Assay** | **Experimental detail/ Target Protein** | **Cell lines** | **Group comparisons** | **Mean Diff.** | **95% CI of diff.** | **P value** |
| --- | --- | --- | --- | --- | --- | --- | --- |
| Figure 2E | Western blot | E_2_ induce cell proliferation/ HER2 | MCF-7 | -E2 |  |  |  |
|  |  |  |  | IVM 0 µM vs. IVM 8 µM | 0.4010 | 0.04498 to 0.7570 | 0.0261 |
|  |  |  |  | IVM 0 µM vs. IVM 10 µM | 0.3903 | 0.03431 to 0.7464 | 0.0305 |
|  |  |  |  | IVM 0 µM vs. IVM 13 µM | 0.4850 | 0.1290 to 0.8410 | 0.0075 |
|  |  |  |  |  |  |  |  |
|  |  |  |  | +E2 |  |  |  |
|  |  |  |  | IVM 0 µM vs. IVM 8 µM | 0.3920 | 0.03598 to 0.7480 | 0.0298 |
|  |  |  |  | IVM 0 µM vs. IVM 10 µM | 0.3773 | 0.02131 to 0.7334 | 0.0369 |
|  |  |  |  | IVM 0 µM vs. IVM 13 µM | 0.6890 | 0.3330 to 1.045 | 0.0004 |
|  |  |  |  |  |  |  |  |
|  |  |  |  | -E2 vs. +E2 |  |  |  |
|  |  |  |  | IVM 0 µM | 0.2123 | -0.1727 to 0.5974 | 0.457 |
|  |  |  |  | IVM 8 µM | 0.2033 | -0.1817 to 0.5884 | 0.4976 |
|  |  |  |  | IVM 10 µM | 0.1993 | -0.1857 to 0.5844 | 0.5161 |
|  |  |  |  | IVM 13 µM | 0.4163 | 0.03131 to 0.8014 | 0.0314 |

| **Figure** | **Assay** | **Experimental detail/ Target Protein** | **Cell lines** | **Group comparisons** | **Mean Diff.** | **95% CI of diff.** | **P value** |
| --- | --- | --- | --- | --- | --- | --- | --- |
| Figure 2F | qPCR | E_2_ induce cell proliferation/ ERα | MCF-7 | -E2 |  |  |  |
|  |  |  |  | IVM 0 µM vs. IVM 8 µM | -0.8770 | -1.156 to -0.5982 | 0.0009 |
|  |  |  |  | IVM 0 µM vs. IVM 10 µM | -0.9910 | -1.016 to -0.9660 | <0.0001 |
|  |  |  |  | IVM 0 µM vs. IVM 13 µM | -0.9787 | -1.038 to -0.9194 | <0.0001 |
|  |  |  |  |  |  |  |  |
|  |  |  |  | +E2 |  |  |  |
|  |  |  |  | IVM 0 µM vs. IVM 8 µM | 0.0857 | -2.087 to 2.258 | 0.9181 |
|  |  |  |  | IVM 0 µM vs. IVM 10 µM | -0.8483 | -1.511 to -0.1853 | 0.0238 |
|  |  |  |  | IVM 0 µM vs. IVM 13 µM | -0.8693 | -1.514 to -0.2251 | 0.02 |
|  |  |  |  |  |  |  |  |
|  |  |  |  | -E2 vs. +E2 |  |  |  |
|  |  |  |  | IVM 0 µM | 0.0730 | -1.039 to 1.185 | 0.9996 |
|  |  |  |  | IVM 8 µM | -0.8897 | -2.002 to 0.2228 | 0.1488 |
|  |  |  |  | IVM 10 µM | -0.0697 | -1.182 to 1.043 | 0.9996 |
|  |  |  |  | IVM 13 µM | -0.0363 | -1.149 to 1.076 | >0.9999 |

| **Figure** | **Assay** | **Experimental detail** | **Cell lines** | **Group comparisons** | **Mean Diff.** | **95% CI of diff.** | **P value** |
| --- | --- | --- | --- | --- | --- | --- | --- |
| Figure 3A | MTT | Combine drugs/Cell Growth | MCF-7 | IVM 0 µM vs. 4-OHT 5 µM | 7.2470 | -1.784 to 16.28 | 0.1163 |
|  |  |  |  | IVM 0 µM vs. IVM 8 µM | 29.5800 | 20.55 to 38.61 | <0.0001 |
|  |  |  |  | IVM 0 µM vs.4-OHT 5 µM + IVM 8 µM | 53.4800 | 44.45 to 62.51 | <0.0001 |
|  |  |  |  | 4-OHT 5 µM vs.4-OHT 5 µM + IVM 8 µM | 46.2300 | 36.19 to 56.27 | <0.0001 |
|  |  |  |  | IVM 8 µM vs.4-OHT 5 µM + IVM 8 µM | 23.9000 | 13.86 to 33.94 | 0.0003 |
| Figure 3B | MTT | Combine drugs/Cell Growth | MCF-7/LCC2 | IVM 0 µM vs. 4-OHT 9 µM | 12.3300 | 3.888 to 20.78 | 0.0076 |
|  |  |  |  | IVM 0 µM vs. IVM 5 µM | 18.1100 | 9.662 to 26.55 | 0.0007 |
|  |  |  |  | IVM 0 µM vs.4-OHT 9 µM + IVM 5 µM | 44.8100 | 36.36 to 53.25 | <0.0001 |
|  |  |  |  | 4-OHT 9 µM vs.4-OHT 9 µM + IVM 5 µM | 32.4700 | 23.08 to 41.86 | <0.0001 |
|  |  |  |  | IVM 5 µM vs.4-OHT 9 µM + IVM 5 µM | 26.7000 | 17.31 to 36.09 | <0.0001 |
| Figure 3C | MTT | Combine drugs/Cell Growth | MCF-7/LCC9 | IVM 0 µM vs. 4-OHT 8 µM | 10.5600 | 0.3194 to 20.81 | 0.0438 |
|  |  |  |  | IVM 0 µM vs. IVM 4 µM | 23.4600 | 13.22 to 33.70 | 0.0005 |
|  |  |  |  | IVM 0 µM vs.4-OHT 8 µM + IVM 4 µM | 40.2200 | 29.98 to 50.47 | <0.0001 |
|  |  |  |  | 4-OHT 8 µM vs.4-OHT 8 µM + IVM 4 µM | 29.6600 | 18.27 to 41.05 | 0.0001 |
|  |  |  |  | IVM 4 µM vs.4-OHT 8 µM + IVM 4 µM | 16.7600 | 5.372 to 28.15 | 0.0066 |

| **Figure** | **Assay** | | **Experimental detail/  Target Protein** | | **Cell lines** | | **Group comparisons** | | | **Mean Diff.** | | **95% CI of diff.** | | **P value** | |
| --- | --- | --- | --- | --- | --- | --- | --- | --- | --- | --- | --- | --- | --- | --- | --- |
| Figure 3E | Western blot | | Combine drugs/ ERα | | MCF-7 | | IVM 0 µM vs. 4-OHT 5 µM | | | -0.4100 | | -1.011 to 0.1911 | | 0.2071 | |
|  |  | |  | |  | | IVM 0 µM vs. IVM 8 µM | | | 0.2367 | | -0.3644 to 0.8377 | | 0.6095 | |
|  |  | |  | |  | | IVM 0 µM vs.4-OHT 5 µM + IVM 8 µM | | | 0.2167 | | -0.3844 to 0.8177 | | 0.6692 | |
|  |  | |  | |  | | 4-OHT 5 µM vs.4-OHT 5 µM + IVM 8 µM | | | 0.6267 | | 0.02562 to 1.228 | | 0.0413 | |
|  |  | |  | |  | | IVM 8 µM vs.4-OHT 5 µM + IVM 8 µM | | | -0.0200 | | -0.6211 to 0.5811 | | 0.9995 | |
| Figure 3F | Western blot | | Combine drugs/ ERα | | MCF-7/LCC2 | | IVM 0 µM vs. 4-OHT 9 µM | | | -0.5067 | | -0.8895 to -0.1238 | | 0.0121 | |
|  |  | |  | |  | | IVM 0 µM vs. IVM 5 µM | | | 0.1700 | | -0.2129 to 0.5529 | | 0.5211 | |
|  |  | |  | |  | | IVM 0 µM vs.4-OHT 9 µM + IVM 5 µM | | | 0.3533 | | -0.02954 to 0.7362 | | 0.0709 | |
|  |  | |  | |  | | 4-OHT 9 µM vs.4-OHT 9 µM + IVM 5 µM | | | 0.8600 | | 0.4771 to 1.243 | | 0.0004 | |
|  |  | |  | |  | | IVM 5 µM vs.4-OHT 9 µM + IVM 5 µM | | | 0.1833 | | -0.1995 to 0.5662 | | 0.463 | |
| Figure 3G | Western blot | | Combine drugs/ ERα | | MCF-7/LCC9 | | IVM 0 µM vs. 4-OHT 8 µM | | | -0.8267 | | -1.422 to -0.2316 | | 0.0092 | |
|  |  | |  | |  | | IVM 0 µM vs. IVM 4 µM | | | -0.1367 | | -0.7318 to 0.4584 | | 0.8802 | |
|  |  | |  | |  | | IVM 0 µM vs.4-OHT 8 µM + IVM 4 µM | | | -0.2367 | | -0.8318 to 0.3584 | | 0.6025 | |
|  |  | |  | |  | | 4-OHT 8 µM vs.4-OHT 8 µM + IVM 4 µM | | | 0.5900 | | -0.005098 to 1.185 | | 0.052 | |
|  |  | |  | |  | | IVM 4 µM vs.4-OHT 8 µM + IVM 4 µM | | | -0.1000 | | -0.6951 to 0.4951 | | 0.9472 | |
| Figure 3H | Western blot | | Combine drugs/ HER2 | | MCF-7 | | IVM 0 µM vs. 4-OHT 5 µM | | | 0.0500 | | -0.3238 to 0.4238 | | 0.9719 | |
|  |  | |  | |  | | IVM 0 µM vs. IVM 8 µM | | | 0.1033 | | -0.2705 to 0.4772 | | 0.8127 | |
|  |  | |  | |  | | IVM 0 µM vs.4-OHT 5 µM + IVM 8 µM | | | 0.4967 | | 0.1228 to 0.8705 | | 0.0119 | |
|  |  | |  | |  | | 4-OHT 5 µM vs.4-OHT 5 µM + IVM 8 µM | | | 0.4467 | | 0.07283 to 0.8205 | | 0.0211 | |
|  |  | |  | |  | | IVM 8 µM vs.4-OHT 5 µM + IVM 8 µM | | | 0.3933 | | 0.01950 to 0.7672 | | 0.0396 | |
| Figure 3I | Western blot | | Combine drugs/ HER2 | | MCF-7/LCC2 | | IVM 0 µM vs. 4-OHT 9 µM | | | 0.3967 | | 0.1398 to 0.6535 | | 0.0049 | |
|  |  | |  | |  | | IVM 0 µM vs. IVM 5 µM | | | 0.2133 | | -0.04352 to 0.4702 | | 0.1075 | |
|  |  | |  | |  | | IVM 0 µM vs.4-OHT 9 µM + IVM 5 µM | | | 0.4867 | | 0.2298 to 0.7435 | | 0.0014 | |
|  |  | |  | |  | | 4-OHT 9 µM vs.4-OHT 9 µM + IVM 5 µM | | | 0.0900 | | -0.1669 to 0.3469 | | 0.6872 | |
|  |  | |  | |  | | IVM 5 µM vs.4-OHT 9 µM + IVM 5 µM | | | 0.2733 | | 0.01648 to 0.5302 | | 0.0375 | |
| Figure 3J | Western blot | | Combine drugs/ HER2 | | MCF-7/LCC9 | | IVM 0 µM vs. 4-OHT 8 µM | | | -0.0933 | | -0.5215 to 0.3348 | | 0.895 | |
|  |  | |  | |  | | IVM 0 µM vs. IVM 4 µM | | | 0.0800 | | -0.3482 to 0.5082 | | 0.9298 | |
|  |  | |  | |  | | IVM 0 µM vs.4-OHT 8 µM + IVM 4 µM | | | 0.2067 | | -0.2215 to 0.6348 | | 0.4568 | |
|  |  | |  | |  | | 4-OHT 8 µM vs.4-OHT 8 µM + IVM 4 µM | | | 0.3000 | | -0.1282 to 0.7282 | | 0.1912 | |
|  |  | |  | |  | | IVM 4 µM vs.4-OHT 8 µM + IVM 4 µM | | | 0.1267 | | -0.3015 to 0.5548 | | 0.7814 | |
| **Figure** | | **Assay** | | **Experimental detail/ Target Protein** | | **Cell lines** | | **Group comparisons** | **Mean Diff.** | | **95% CI of diff.** | | **P value** | |  |
| Figure 4B | | Western blot | | pSMAD2/SMAD2 | | MCF-7 | | IVM 0 µM vs. IVM 3 µM | 0.2510 | | 0.07648 to 0.4255 | | 0.0083 | |  |
|  | |  | |  | |  | | IVM 0 µM vs. IVM 6 µM | 0.3273 | | 0.1528 to 0.5019 | | 0.0017 | |  |
|  | |  | |  | |  | | IVM 0 µM vs. IVM 9 µM | 0.3530 | | 0.1785 to 0.5275 | | 0.001 | |  |
| Figure 4C | | Western blot | | pSMAD2/SMAD2 | | MCF-7/LCC2 | | IVM 0 µM vs. IVM 3 µM | 0.3670 | | 0.2866 to 0.4474 | | <0.0001 | |  |
|  | |  | |  | |  | | IVM 0 µM vs. IVM 6 µM | 0.5840 | | 0.5036 to 0.6644 | | <0.0001 | |  |
|  | |  | |  | |  | | IVM 0 µM vs. IVM 9 µM | 0.7350 | | 0.6546 to 0.8154 | | <0.0001 | |  |
| Figure 4D | | Western blot | | pSMAD2/SMAD2 | | MCF-7/LCC9 | | IVM 0 µM vs. IVM 3 µM | 0.2297 | | 0.1390 to 0.3204 | | 0.0002 | |  |
|  | |  | |  | |  | | IVM 0 µM vs. IVM 6 µM | 0.4787 | | 0.3880 to 0.5694 | | <0.0001 | |  |
|  | |  | |  | |  | | IVM 0 µM vs. IVM 9 µM | 0.5950 | | 0.5043 to 0.6857 | | <0.0001 | |  |
| Figure 4E | | Western blot | | pERK/ERK | | MCF-7 | | IVM 0 µM vs. IVM 3 µM | 0.5037 | | 0.3823 to 0.6250 | | <0.0001 | |  |
|  | |  | |  | |  | | IVM 0 µM vs. IVM 6 µM | 0.6407 | | 0.5193 to 0.7620 | | <0.0001 | |  |
|  | |  | |  | |  | | IVM 0 µM vs. IVM 9 µM | 0.6697 | | 0.5483 to 0.7910 | | <0.0001 | |  |
| Figure 4F | | Western blot | | pERK/ERK | | MCF-7/LCC2 | | IVM 0 µM vs. IVM 3 µM | 0.0886 | | -0.08373 to 0.2610 | | 0.3719 | |  |
|  | |  | |  | |  | | IVM 0 µM vs. IVM 6 µM | 0.4922 | | 0.3198 to 0.6646 | | 0.0001 | |  |
|  | |  | |  | |  | | IVM 0 µM vs. IVM 9 µM | 0.9360 | | 0.7636 to 1.108 | | <0.0001 | |  |
| Figure 4G | | Western blot | | pERK/ERK | | MCF-7/LCC9 | | IVM 0 µM vs. IVM 3 µM | -0.1860 | | -0.5912 to 0.2192 | | 0.4526 | |  |
|  | |  | |  | |  | | IVM 0 µM vs. IVM 6 µM | 0.1353 | | -0.2698 to 0.5405 | | 0.6677 | |  |
|  | |  | |  | |  | | IVM 0 µM vs. IVM 9 µM | 0.6901 | | 0.2849 to 1.095 | | 0.0031 | |  |

| **Figure** | **Assay** | **Experimental detail/ Target Protein** | | **Cell lines** | | **Group comparisons** | | **Mean Diff.** | | **95% CI of diff.** | | **P value** | |  |
| --- | --- | --- | --- | --- | --- | --- | --- | --- | --- | --- | --- | --- | --- | --- |
| Figure S1 | MTT | Time-dependent | | MCF-7 | | IVM 3.12 μM | |  | |  | |  | |  |
|  |  |  | |  | | 24h vs. 48h | | 22.34 | | 15.62 to 29.06 | | <0.0001 | |  |
|  |  |  | |  | | 24h vs. 72h | | 30.40 | | 23.68 to 37.12 | | <0.0001 | |  |
|  |  |  | |  | | IVM 6.25 μM | |  | |  | |  | |  |
|  |  |  | |  | | 24h vs. 48h | | 32.83 | | 26.11 to 39.55 | | <0.0001 | |  |
|  |  |  | |  | | 24h vs. 72h | | 46.00 | | 39.28 to 52.72 | | <0.0001 | |  |
|  |  |  | |  | | IVM 12.5 μM | |  | |  | |  | |  |
|  |  |  | |  | | 24h vs. 48h | | 24.49 | | 17.77 to 31.21 | | <0.0001 | |  |
|  |  |  | |  | | 24h vs. 72h | | 30.84 | | 24.12 to 37.56 | | <0.0001 | |  |
|  | MTT | Time-dependent | | MCF-7/LCC2 | | IVM 3.12 μM | |  | |  | |  | |  |
|  |  |  | |  | | 24h vs. 48h | | 10.79 | | 2.821 to 18.76 | | 0.0084 | |  |
|  |  |  | |  | | 24h vs. 72h | | 15.03 | | 7.057 to 23.00 | | 0.0005 | |  |
|  |  |  | |  | | IVM 6.25 μM | |  | |  | |  | |  |
|  |  |  | |  | | 24h vs. 48h | | 14.68 | | 6.707 to 22.65 | | 0.0007 | |  |
|  |  |  | |  | | 24h vs. 72h | | 23.26 | | 15.29 to 31.23 | | <0.0001 | |  |
|  |  |  | |  | | IVM 12.5 μM | |  | |  | |  | |  |
|  |  |  | |  | | 24h vs. 48h | | 26.12 | | 18.15 to 34.09 | | <0.0001 | |  |
|  |  |  | |  | | 24h vs. 72h | | 33.97 | | 26.00 to 41.94 | | <0.0001 | |  |
|  | MTT | Time-dependent | | MCF-7/LCC9 | | IVM 3.12 μM | |  | |  | |  | |  |
|  |  |  | |  | | 24h vs. 48h | | 10.99 | | -1.691 to 23.67 | | 0.093 | |  |
|  |  |  | |  | | 24h vs. 72h | | 5.22 | | -7.464 to 17.90 | | 0.5233 | |  |
|  |  |  | |  | | IVM 6.25 μM | |  | |  | |  | |  |
|  |  |  | |  | | 24h vs. 48h | | 12.61 | | -0.06719 to 25.29 | | 0.0513 | |  |
|  |  |  | |  | | 24h vs. 72h | | 24.21 | | 11.53 to 36.89 | | 0.0005 | |  |
|  |  |  | |  | | IVM 12.5 μM | |  | |  | |  | |  |
|  |  |  | |  | | 24h vs. 48h | | 28.29 | | 15.61 to 40.97 | | <0.0001 | |  |
|  |  |  | |  | | 24h vs. 72h | | 31.79 | | 19.11 to 44.47 | | <0.0001 | |  |
| **Figure** | **Assay** | | **Experimental detail/ Target Protein** | | **Cell lines** | | **Group comparisons** | | **Mean Diff.** | | **95% CI of diff.** | | **P value** | |
| Figure S2 | MTT | | Combined IVM & 4-OHT | | IVM 0 μM | | CRL-1474 vs. MCF-7 | |  | |  | |  | |
|  |  | |  | |  | | 4-OHT 0 μM | | 0.00 | | -7.896 to 7.896 | | >0.9999 | |
|  |  | |  | |  | | 4-OHT 2.5 μM | | 0.11 | | -7.786 to 8.006 | | >0.9999 | |
|  |  | |  | |  | | 4-OHT 5 μM | | 6.50 | | -1.396 to 14.40 | | 0.1417 | |
|  |  | |  | |  | | 4-OHT 7.5 μM | | 29.11 | | 21.22 to 37.01 | | <0.0001 | |
|  |  | |  | |  | | 4-OHT 10 μM | | 74.50 | | 66.60 to 82.39 | | <0.0001 | |
|  |  | |  | |  | |  | |  | |  | |  | |
|  |  | |  | | IVM 3 μM | | CRL-1474 vs. MCF-7 | |  | |  | |  | |
|  |  | |  | |  | | 4-OHT 0 μM | | 14.25 | | 1.228 to 27.27 | | 0.0277 | |
|  |  | |  | |  | | 4-OHT 2.5 μM | | 13.71 | | 0.6882 to 26.73 | | 0.036 | |
|  |  | |  | |  | | 4-OHT 5 μM | | 17.65 | | 4.635 to 30.67 | | 0.005 | |
|  |  | |  | |  | | 4-OHT 7.5 μM | | 38.78 | | 25.76 to 51.80 | | <0.0001 | |
|  |  | |  | |  | | 4-OHT 10 μM | | 74.32 | | 61.30 to 87.34 | | <0.0001 | |
|  |  | |  | |  | |  | |  | |  | |  | |
|  |  | |  | | IVM 9 μM | | CRL-1474 vs. MCF-7 | |  | |  | |  | |
|  |  | |  | |  | | 4-OHT 0 μM | | 42.60 | | 30.75 to 54.45 | | <0.0001 | |
|  |  | |  | |  | | 4-OHT 2.5 μM | | 37.18 | | 25.33 to 49.03 | | <0.0001 | |
|  |  | |  | |  | | 4-OHT 5 μM | | 44.11 | | 32.26 to 55.96 | | <0.0001 | |
|  |  | |  | |  | | 4-OHT 7.5 μM | | 74.77 | | 62.92 to 86.62 | | <0.0001 | |
|  |  | |  | |  | | 4-OHT 10 μM | | 54.45 | | 42.60 to 66.30 | | <0.0001 | |
| Figure S3 | qPCR | | mono-treatment/ pS2 | | MCF-7 | | IVM 0 µM vs. IVM 8 µM | | 0.6243 | | 0.09890 to 1.150 | | 0.0226 | |
|  |  | |  | |  | | IVM 0 µM vs. IVM 10 µM | | 0.7753 | | 0.2499 to 1.301 | | 0.0072 | |
|  |  | |  | |  | | IVM 0 µM vs. IVM 13 µM | | 0.5683 | | 0.04290 to 1.094 | | 0.0354 | |

| **Figure** | **Assay** | **Experimental detail/ Target Protein** | **Cell lines** | **Group comparisons** | **Mean Diff.** | **95% CI of diff.** | **P value** |
| --- | --- | --- | --- | --- | --- | --- | --- |
| Figure S4B | Western blot | SMAD4 | MCF-7 | IVM 0 µM vs. IVM 3 µM | -0.1533 | -1.571 to 1.264 | 0.9785 |
|  |  |  |  | IVM 0 µM vs. IVM 6 µM | -0.5100 | -1.928 to 0.9076 | 0.6212 |
|  |  |  |  | IVM 0 µM vs. IVM 9 µM | -0.2033 | -1.621 to 1.214 | 0.9532 |
| Figure S4C | Western blot | SMAD4 | MCF-7/LCC2 | IVM 0 µM vs. IVM 3 µM | -0.1633 | -1.096 to 0.7697 | 0.9212 |
|  |  |  |  | IVM 0 µM vs. IVM 6 µM | -0.2767 | -1.210 to 0.6564 | 0.7353 |
|  |  |  |  | IVM 0 µM vs. IVM 9 µM | -0.6067 | -1.540 to 0.3264 | 0.2193 |
| Figure S4D | Western blot | SMAD4 | MCF-7/LCC9 | IVM 0 µM vs. IVM 3 µM | 0.0400 | -0.4899 to 0.5699 | 0.9924 |
|  |  |  |  | IVM 0 µM vs. IVM 6 µM | 0.0067 | -0.5232 to 0.5366 | >0.9999 |
|  |  |  |  | IVM 0 µM vs. IVM 9 µM | 0.1767 | -0.3532 to 0.7066 | 0.6689 |
| Figure S4E | Western blot | pSMAD3/SMAD3 | MCF-7 | IVM 0 µM vs. IVM 3 µM | 0.2000 | -0.05424 to 0.4542 | 0.1243 |
|  |  |  |  | IVM 0 µM vs. IVM 6 µM | 0.3133 | 0.05909 to 0.5676 | 0.0189 |
|  |  |  |  | IVM 0 µM vs. IVM 9 µM | 0.5567 | 0.3024 to 0.8109 | 0.0006 |
|  |  | SMAD3 | MCF-7 | IVM 0 µM vs. IVM 3 µM | 0.0267 | -0.2163 to 0.2696 | 0.9777 |
|  |  |  |  | IVM 0 µM vs. IVM 6 µM | -0.3633 | -0.6063 to -0.1204 | 0.0066 |
|  |  |  |  | IVM 0 µM vs. IVM 9 µM | -0.3633 | -0.6063 to -0.1204 | 0.0066 |
| Figure S4F | Western blot | pSMAD3/SMAD3 | MCF-7/LCC2 | IVM 0 µM vs. IVM 3 µM | 0.2133 | -0.2277 to 0.6544 | 0.4153 |
|  |  |  |  | IVM 0 µM vs. IVM 6 µM | 0.4800 | 0.03896 to 0.9210 | 0.0344 |
|  |  |  |  | IVM 0 µM vs. IVM 9 µM | 0.5600 | 0.1190 to 1.001 | 0.0162 |
|  |  | SMAD3 | MCF-7/LCC2 | IVM 0 µM vs. IVM 3 µM | -0.5067 | -3.205 to 2.192 | 0.9059 |
|  |  |  |  | IVM 0 µM vs. IVM 6 µM | -1.2970 | -3.995 to 1.402 | 0.4199 |
|  |  |  |  | IVM 0 µM vs. IVM 9 µM | -1.5000 | -4.199 to 1.199 | 0.3182 |
| Figure S4G | Western blot | pSMAD3/SMAD3 | MCF-7/LCC9 | IVM 0 µM vs. IVM 3 µM | -0.5400 | -2.423 to 1.343 | 0.7525 |
|  |  |  |  | IVM 0 µM vs. IVM 6 µM | 0.3367 | -1.547 to 2.220 | 0.9168 |
|  |  |  |  | IVM 0 µM vs. IVM 9 µM | 0.2800 | -1.603 to 2.163 | 0.9485 |
|  |  | SMAD3 | MCF-7/LCC9 | IVM 0 µM vs. IVM 3 µM | -0.8133 | -1.882 to 0.2552 | 0.1384 |
|  |  |  |  | IVM 0 µM vs. IVM 6 µM | -1.0500 | -2.119 to 0.01850 | 0.0538 |
|  |  |  |  | IVM 0 µM vs. IVM 9 µM | -0.5500 | -1.619 to 0.5185 | 0.3711 |

| **Figure** | **Assay** | **Experimental detail/ Target Protein** | **Cell lines** | **Group comparisons** | **Mean Diff.** | **95% CI of diff.** | **P value** |
| --- | --- | --- | --- | --- | --- | --- | --- |
| Figure S4H | Western blot | pPAK-1/PAK-1 | MCF-7 | IVM 0 µM vs. IVM 3 µM | -0.2343 | -0.6179 to 0.1492 | 0.2566 |
|  |  |  |  | IVM 0 µM vs. IVM 6 µM | -0.1220 | -0.5055 to 0.2615 | 0.6965 |
|  |  |  |  | IVM 0 µM vs. IVM 9 µM | 0.0277 | -0.3559 to 0.4112 | 0.9933 |
|  |  | PAK-1 | MCF-7 | IVM 0 µM vs. IVM 3 µM | -0.0933 | -0.3967 to 0.2100 | 0.7152 |
|  |  |  |  | IVM 0 µM vs. IVM 6 µM | 0.0533 | -0.2500 to 0.3567 | 0.9203 |
|  |  |  |  | IVM 0 µM vs. IVM 9 µM | 0.0300 | -0.2733 to 0.3333 | 0.9833 |
| Figure S4I | Western blot | pPAK-1/PAK-1 | MCF-7/LCC2 | IVM 0 µM vs. IVM 3 µM | 0.0098 | -0.1763 to 0.1958 | 0.9974 |
|  |  |  |  | IVM 0 µM vs. IVM 6 µM | -0.0024 | -0.1885 to 0.1837 | >0.9999 |
|  |  |  |  | IVM 0 µM vs. IVM 9 µM | 0.2761 | 0.09000 to 0.4621 | 0.0069 |
|  |  | PAK-1 | MCF-7/LCC2 | IVM 0 µM vs. IVM 3 µM | -0.1529 | -0.3967 to 0.09094 | 0.2408 |
|  |  |  |  | IVM 0 µM vs. IVM 6 µM | 0.0504 | -0.1934 to 0.2942 | 0.8807 |
|  |  |  |  | IVM 0 µM vs. IVM 9 µM | 0.2973 | 0.05346 to 0.5411 | 0.0199 |
| Figure S4J | Western blot | pPAK-1/PAK-1 | MCF-7/LCC9 | IVM 0 µM vs. IVM 3 µM | -0.3904 | -1.339 to 0.5584 | 0.5305 |
|  |  |  |  | IVM 0 µM vs. IVM 6 µM | -0.2948 | -1.244 to 0.6540 | 0.7098 |
|  |  |  |  | IVM 0 µM vs. IVM 9 µM | -0.4353 | -1.384 to 0.5135 | 0.4531 |
|  |  | PAK-1 | MCF-7/LCC9 | IVM 0 µM vs. IVM 3 µM | 0.1270 | -0.7935 to 1.047 | 0.9579 |
|  |  |  |  | IVM 0 µM vs. IVM 6 µM | -0.2070 | -1.127 to 0.7135 | 0.8545 |
|  |  |  |  | IVM 0 µM vs. IVM 9 µM | -0.0763 | -0.9968 to 0.8442 | 0.9900 |
| Figure S5A | Western blot | E2 induce cell proliferation/ ERα | MCF-7 | -E2 vs. +E2 |  |  |  |
|  |  |  |  | IVM 0 µM | -135.3000 | -160.8 to -109.7 | <0.0001 |
|  |  |  |  | IVM 1 µM | -109.5000 | -135.1 to -83.95 | <0.0001 |
|  |  |  |  | IVM 3 µM | -73.8800 | -99.47 to -48.29 | <0.0001 |
|  |  |  |  | IVM 5 µM | -31.9200 | -57.51 to -6.333 | 0.0119 |
| Figure S5B | Western blot | E2 induce cell proliferation/ ERα | T-47D | -E2 vs. +E2 |  |  |  |
|  |  |  |  | IVM 0 µM | -66.8400 | -87.07 to -46.62 | <0.0001 |
|  |  |  |  | IVM 1 µM | -59.5300 | -79.75 to -39.30 | <0.0001 |
|  |  |  |  | IVM 3 µM | -53.2100 | -73.44 to -32.98 | <0.0001 |
|  |  |  |  | IVM 5 µM | -6.5900 | -26.82 to 13.64 | 0.847 |

| **Figure** | **Assay** | **Experimental detail** | **Cell lines** | **Group comparisons** | **Mean Diff.** | **95% CI of diff.** | **P value** |
| --- | --- | --- | --- | --- | --- | --- | --- |
| Figure S5C | MTT | E_2_ induce cell proliferation | MCF-7/LCC2 | -E2 |  |  |  |
|  |  |  |  | IVM 0 µM vs. IVM 1 µM | 6.9200 | -4.513 to 18.35 | 0.364 |
|  |  |  |  | IVM 0 µM vs. IVM 3 µM | 41.2500 | 29.81 to 52.68 | <0.0001 |
|  |  |  |  | IVM 0 µM vs. IVM 5 µM | 69.0300 | 57.59 to 80.46 | <0.0001 |
|  |  |  |  | IVM 0 µM vs. 4-OHT 5 µM | 33.3200 | 21.88 to 44.75 | <0.0001 |
|  |  |  |  | IVM 0 µM vs. 4-OHT 10 µM | 92.7500 | 81.32 to 104.2 | <0.0001 |
|  |  |  |  |  |  |  |  |
|  |  |  |  | +E2 |  |  |  |
|  |  |  |  | IVM 0 µM vs. IVM 1 µM | 7.3030 | -4.129 to 18.74 | 0.3164 |
|  |  |  |  | IVM 0 µM vs. IVM 3 µM | 35.2400 | 23.81 to 46.68 | <0.0001 |
|  |  |  |  | IVM 0 µM vs. IVM 5 µM | 69.7000 | 58.27 to 81.13 | <0.0001 |
|  |  |  |  | IVM 0 µM vs. 4-OHT 5 µM | 20.6100 | 9.177 to 32.04 | 0.0003 |
|  |  |  |  | IVM 0 µM vs. 4-OHT 10 µM | 82.8700 | 71.43 to 94.30 | <0.0001 |
|  |  |  |  |  |  |  |  |
|  |  |  |  | -E2 vs. +E2 |  |  |  |
|  |  |  |  | IVM 0 µM | 11.1000 | -1.053 to 23.26 | 0.0872 |
|  |  |  |  | IVM 1 µM | 11.4900 | -0.6694 to 23.64 | 0.0714 |
|  |  |  |  | IVM 3 µM | 5.1000 | -7.056 to 17.26 | 0.8087 |
|  |  |  |  | IVM 5 µM | 11.7800 | -0.3794 to 23.93 | 0.0613 |
|  |  |  |  | 4-OHT 5 µM | -1.6030 | -13.76 to 10.55 | 0.9994 |
|  |  |  |  | 4-OHT 10 µM | 1.2170 | -10.94 to 13.37 | 0.9999 |

| **Figure** | **Assay** | **Experimental detail** | **Cell lines** | **Group comparisons** | **Mean Diff.** | **95% CI of diff.** | **P value** |
| --- | --- | --- | --- | --- | --- | --- | --- |
| Figure S5D | MTT | E_2_ induce cell proliferation | MCF-7/LCC9 | -E2 |  |  |  |
|  |  |  |  | IVM 0 µM vs. IVM 1 µM | 18.5800 | 14.06 to 23.11 | <0.0001 |
|  |  |  |  | IVM 0 µM vs. IVM 3 µM | 27.8500 | 23.32 to 32.37 | <0.0001 |
|  |  |  |  | IVM 0 µM vs. IVM 5 µM | 71.6900 | 67.16 to 76.21 | <0.0001 |
|  |  |  |  | IVM 0 µM vs. 4-OHT 5 µM | 48.0000 | 43.48 to 52.52 | <0.0001 |
|  |  |  |  | IVM 0 µM vs. 4-OHT 10 µM | 96.6800 | 92.15 to 101.2 | <0.0001 |
|  |  |  |  |  |  |  |  |
|  |  |  |  | +E2 |  |  |  |
|  |  |  |  | IVM 0 µM vs. IVM 1 µM | 8.4600 | 3.938 to 12.98 | 0.0002 |
|  |  |  |  | IVM 0 µM vs. IVM 3 µM | 30.4100 | 25.88 to 34.93 | <0.0001 |
|  |  |  |  | IVM 0 µM vs. IVM 5 µM | 63.7200 | 59.20 to 68.24 | <0.0001 |
|  |  |  |  | IVM 0 µM vs. 4-OHT 5 µM | 42.9600 | 38.44 to 47.48 | <0.0001 |
|  |  |  |  | IVM 0 µM vs. 4-OHT 10 µM | 93.5600 | 89.03 to 98.08 | <0.0001 |
|  |  |  |  |  |  |  |  |
|  |  |  |  | -E2 vs. +E2 |  |  |  |
|  |  |  |  | IVM 0 µM | 3.6070 | -1.202 to 8.415 | 0.2263 |
|  |  |  |  | IVM 1 µM | -6.5170 | -11.32 to -1.708 | 0.0042 |
|  |  |  |  | IVM 3 µM | 6.1670 | 1.358 to 10.97 | 0.0071 |
|  |  |  |  | IVM 5 µM | -4.3600 | -9.168 to 0.4483 | 0.0908 |
|  |  |  |  | 4-OHT 5 µM | -1.4330 | -6.242 to 3.375 | 0.954 |
|  |  |  |  | 4-OHT 10 µM | 0.4867 | -4.322 to 5.295 | 0.9999 |

| **Figure** | **Assay** | **Experimental detail/ Target Protein** | **Cell lines** | **Group comparisons** | **Mean Diff.** | **95% CI of diff.** | **P value** |
| --- | --- | --- | --- | --- | --- | --- | --- |
| Figure S6B | Western blot | Combine drugs/ ERα | T-47D | IVM 0 µM vs. 4-OHT 9 µM | -0.30 | -0.6980 to 0.09796 | 0.1142 |
|  |  |  |  | IVM 0 µM vs. IVM 8 µM | 0.40 | 0.002044 to 0.7980 | 0.0492 |
|  |  |  |  | IVM 0 µM vs.4-OHT 9 µM + IVM 8 µM | 0.59 | 0.1920 to 0.9880 | 0.0133 |
|  |  |  |  | 4-OHT 9 µM vs.4-OHT 9 µM + IVM 8 µM | 0.89 | 0.4422 to 1.338 | 0.0044 |
|  |  |  |  | IVM 8 µM vs.4-OHT 9 µM + IVM 8 µM | 0.19 | -0.2578 to 0.6378 | 0.4172 |
| Figure S6C | Western blot | Combine drugs/ ERα | T-47D Tam1 | IVM 0 µM vs. 4-OHT 9 µM | 0.515 | -0.4520 to 1.482 | 0.2605 |
|  |  |  |  | IVM 0 µM vs. IVM 8 µM | 0.745 | -0.2220 to 1.712 | 0.1077 |
|  |  |  |  | IVM 0 µM vs.4-OHT 9 µM + IVM 8 µM | 0.985 | 0.01805 to 1.952 | 0.0472 |
|  |  |  |  | 4-OHT 9 µM vs.4-OHT 9 µM + IVM 8 µM | 0.47 | -0.6180 to 1.558 | 0.4052 |
|  |  |  |  | IVM 8 µM vs.4-OHT 9 µM + IVM 8 µM | 0.24 | -0.8480 to 1.328 | 0.8077 |
| Figure S6D | Western blot | Combine drugs/ HER2 | T-47D | IVM 0 µM vs. 4-OHT 9 µM | 0.23 | 0.06520 to 0.3948 | 0.0165 |
|  |  |  |  | IVM 0 µM vs. IVM 8 µM | 0.225 | 0.06020 to 0.3898 | 0.0178 |
|  |  |  |  | IVM 0 µM vs.4-OHT 9 µM + IVM 8 µM | 0.465 | 0.3002 to 0.6298 | 0.0012 |
|  |  |  |  | 4-OHT 9 µM vs.4-OHT 9 µM + IVM 8 µM | 0.235 | 0.04956 to 0.4204 | 0.0226 |
|  |  |  |  | IVM 8 µM vs.4-OHT 9 µM + IVM 8 µM | 0.24 | 0.05456 to 0.4254 | 0.021 |
| Figure S6E | Western blot | Combine drugs/ HER2 | T-47D Tam1 | IVM 0 µM vs. 4-OHT 9 µM | 0.33 | -0.4582 to 1.118 | 0.4016 |
|  |  |  |  | IVM 0 µM vs. IVM 8 µM | 0.43 | -0.3582 to 1.218 | 0.248 |
|  |  |  |  | IVM 0 µM vs.4-OHT 9 µM + IVM 8 µM | 0.695 | -0.09317 to 1.483 | 0.0733 |
|  |  |  |  | 4-OHT 9 µM vs.4-OHT 9 µM + IVM 8 µM | 0.365 | -0.5219 to 1.252 | 0.4378 |
|  |  |  |  | IVM 8 µM vs.4-OHT 9 µM + IVM 8 µM | 0.265 | -0.6219 to 1.152 | 0.6499 |
| Figure S6F | Western blot | Combine drugs/ HER2 | T47D 182R1 | IVM 0 µM vs. 4-OHT 9 µM | 0.41 | -0.06995 to 0.8899 | 0.0815 |
|  |  |  |  | IVM 0 µM vs. IVM 8 µM | 0.36 | -0.1199 to 0.8399 | 0.1185 |
|  |  |  |  | IVM 0 µM vs.4-OHT 9 µM + IVM 8 µM | 0.65 | 0.1701 to 1.130 | 0.0179 |
|  |  |  |  | 4-OHT 9 µM vs.4-OHT 9 µM + IVM 8 µM | 0.24 | -0.2399 to 0.7199 | 0.311 |
|  |  |  |  | IVM 8 µM vs.4-OHT 9 µM + IVM 8 µM | 0.29 | -0.1899 to 0.7699 | 0.2066 |

| **Figure** | **Assay** | **Experimental detail/ Target Protein** | **Cell lines** | **Group comparisons** | **Mean Diff.** | **95% CI of diff.** | **P value** |
| --- | --- | --- | --- | --- | --- | --- | --- |
| Figure S8B | Western blot | PI3K | MCF-7 | IVM 0 µM vs. IVM 3 µM | -0.1417 | -0.6550 to 0.3717 | 0.7713 |
|  |  |  |  | IVM 0 µM vs. IVM 6 µM | 0.0100 | -0.5034 to 0.5234 | >0.9999 |
|  |  |  |  | IVM 0 µM vs. IVM 9 µM | -0.1363 | -0.6497 to 0.3770 | 0.7891 |
| Figure S8C | Western blot | PI3K | MCF-7/LCC2 | IVM 0 µM vs. IVM 3 µM | -0.06767 | -0.5475 to 0.4121 | 0.9553 |
|  |  |  |  | IVM 0 µM vs. IVM 6 µM | -0.0920 | -0.5718 to 0.3878 | 0.9009 |
|  |  |  |  | IVM 0 µM vs. IVM 9 µM | 0.01833 | -0.4615 to 0.4981 | 0.999 |
| Figure S8D | Western blot | PI3K | MCF-7/LCC9 | IVM 0 µM vs. IVM 3 µM | 0.03467 | -0.2792 to 0.3486 | 0.9773 |
|  |  |  |  | IVM 0 µM vs. IVM 6 µM | -0.05133 | -0.3652 to 0.2626 | 0.9339 |
|  |  |  |  | IVM 0 µM vs. IVM 9 µM | -0.2080 | -0.5219 to 0.1059 | 0.2084 |
| Figure S8E | Western blot | pAKT/AKT | MCF-7 | IVM 0 µM vs. IVM 3 µM | -0.1014 | -0.4795 to 0.2766 | 0.7844 |
|  |  |  |  | IVM 0 µM vs. IVM 6 µM | -0.01783 | -0.3959 to 0.3602 | 0.9981 |
|  |  |  |  | IVM 0 µM vs. IVM 9 µM | -0.5223 | -0.9003 to -0.1442 | 0.0103 |
|  |  | AKT | MCF-7 | IVM 0 µM vs. IVM 3 µM | 0.1267 | -0.3357 to 0.5891 | 0.7748 |
|  |  |  |  | IVM 0 µM vs. IVM 6 µM | 0.05333 | -0.4091 to 0.5157 | 0.9743 |
|  |  |  |  | IVM 0 µM vs. IVM 9 µM | 0.03333 | -0.4291 to 0.4957 | 0.9933 |
| Figure S8F | Western blot | pAKT/AKT | MCF-7/LCC2 | IVM 0 µM vs. IVM 3 µM | -0.5295 | -1.344 to 0.2850 | 0.2193 |
|  |  |  |  | IVM 0 µM vs. IVM 6 µM | -0.1993 | -1.014 to 0.6152 | 0.8237 |
|  |  |  |  | IVM 0 µM vs. IVM 9 µM | -0.3014 | -1.116 to 0.5131 | 0.6029 |
|  |  | AKT | MCF-7/LCC2 | IVM 0 µM vs. IVM 3 µM | -0.02193 | -0.4772 to 0.4333 | 0.9979 |
|  |  |  |  | IVM 0 µM vs. IVM 6 µM | 0.04727 | -0.4080 to 0.5025 | 0.9808 |
|  |  |  |  | IVM 0 µM vs. IVM 9 µM | -0.03073 | -0.4860 to 0.4245 | 0.9944 |
| Figure S8G | Western blot | pAKT/AKT | MCF-7/LCC9 | IVM 0 µM vs. IVM 3 µM | -0.3841 | -0.9094 to 0.1412 | 0.1569 |
|  |  |  |  | IVM 0 µM vs. IVM 6 µM | 0.2706 | -0.2547 to 0.7959 | 0.3705 |
|  |  |  |  | IVM 0 µM vs. IVM 9 µM | -1.8000 | -2.325 to -1.275 | <0.0001 |
|  |  | AKT | MCF-7/LCC9 | IVM 0 µM vs. IVM 3 µM | -0.0907 | -0.5439 to 0.3625 | 0.8899 |
|  |  |  |  | IVM 0 µM vs. IVM 6 µM | 0.1026 | -0.3506 to 0.5558 | 0.8523 |
|  |  |  |  | IVM 0 µM vs. IVM 9 µM | 0.0212 | -0.4320 to 0.4744 | 0.9981 |

| **Figure** | **Assay** | **Experimental detail/ Target Protein** | **Cell lines** | **Group comparisons** | **Mean Diff.** | **95% CI of diff.** | **P value** |
| --- | --- | --- | --- | --- | --- | --- | --- |
| Figure S8H | Western blot | p-mTOR/mTOR | MCF-7 | IVM 0 µM vs. IVM 3 µM | -0.4300 | -0.9189 to 0.05885 | 0.0836 |
|  |  |  |  | IVM 0 µM vs. IVM 6 µM | -0.1403 | -0.6292 to 0.3485 | 0.7519 |
|  |  |  |  | IVM 0 µM vs. IVM 9 µM | -0.2147 | -0.7035 to 0.2742 | 0.4845 |
|  |  | mTOR | MCF-7 | IVM 0 µM vs. IVM 3 µM | -0.1097 | -1.011 to 0.7913 | 0.9702 |
|  |  |  |  | IVM 0 µM vs. IVM 6 µM | -0.1270 | -1.028 to 0.7739 | 0.9554 |
|  |  |  |  | IVM 0 µM vs. IVM 9 µM | -0.3840 | -1.285 to 0.5169 | 0.5056 |
| Figure S8I | Western blot | p-mTOR/mTOR | MCF-7/LCC2 | IVM 0 µM vs. IVM 3 µM | -0.1640 | -0.7584 to 0.4304 | 0.7713 |
|  |  |  |  | IVM 0 µM vs. IVM 6 µM | -0.2420 | -0.8364 to 0.3524 | 0.5378 |
|  |  |  |  | IVM 0 µM vs. IVM 9 µM | -0.2140 | -0.8084 to 0.3804 | 0.6207 |
|  |  | mTOR | MCF-7/LCC2 | IVM 0 µM vs. IVM 3 µM | 0.0350 | -0.4247 to 0.4947 | 0.9922 |
|  |  |  |  | IVM 0 µM vs. IVM 6 µM | -0.05567 | -0.5154 to 0.4040 | 0.9706 |
|  |  |  |  | IVM 0 µM vs. IVM 9 µM | 0.04433 | -0.4154 to 0.5040 | 0.9845 |
| Figure S8J | Western blot | p-mTOR/mTOR | MCF-7/LCC9 | IVM 0 µM vs. IVM 3 µM | -0.2897 | -0.8597 to 0.2803 | 0.3802 |
|  |  |  |  | IVM 0 µM vs. IVM 6 µM | -0.1207 | -0.6907 to 0.4493 | 0.8738 |
|  |  |  |  | IVM 0 µM vs. IVM 9 µM | 0.1020 | -0.4680 to 0.6720 | 0.9167 |
|  |  | mTOR | MCF-7/LCC9 | IVM 0 µM vs. IVM 3 µM | -0.0500 | -0.4747 to 0.3747 | 0.9728 |
|  |  |  |  | IVM 0 µM vs. IVM 6 µM | -0.2007 | -0.6254 to 0.2241 | 0.4321 |
|  |  |  |  | IVM 0 µM vs. IVM 9 µM | -0.4343 | -0.8591 to -0.009616 | 0.0454 |
